# Supplementary figures and images for: GSR-DB: a manually curated and optimized taxonomical database for 16S rRNA amplicon analysis
Source: mSystems. 2024 Jan 8;9(2):e00950-23. doi: 10.1128/msystems.00950-23 (PMC10946287; doi:10.1128/msystems.00950-23)

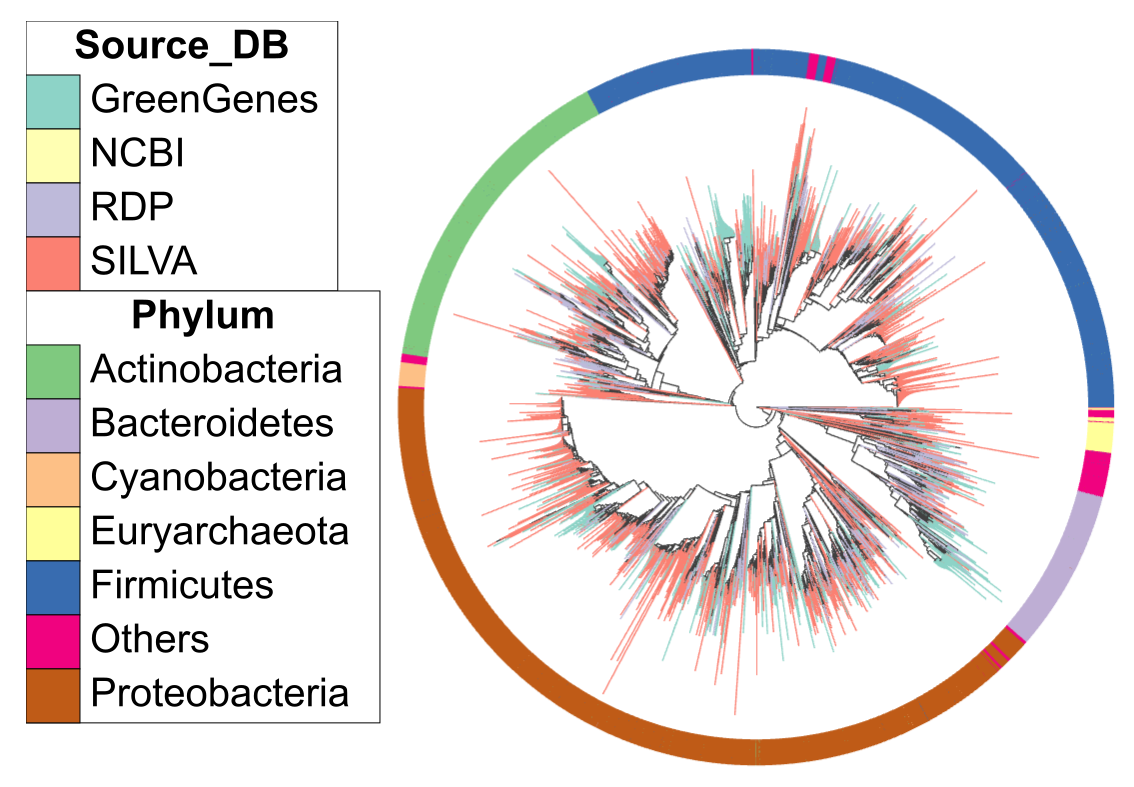

Supplement: Fig. S1 — Visualization of the phylogenetic tree for GSR-DB, rendered using Empress. [file msystems.00950-23-s0001.tiff]

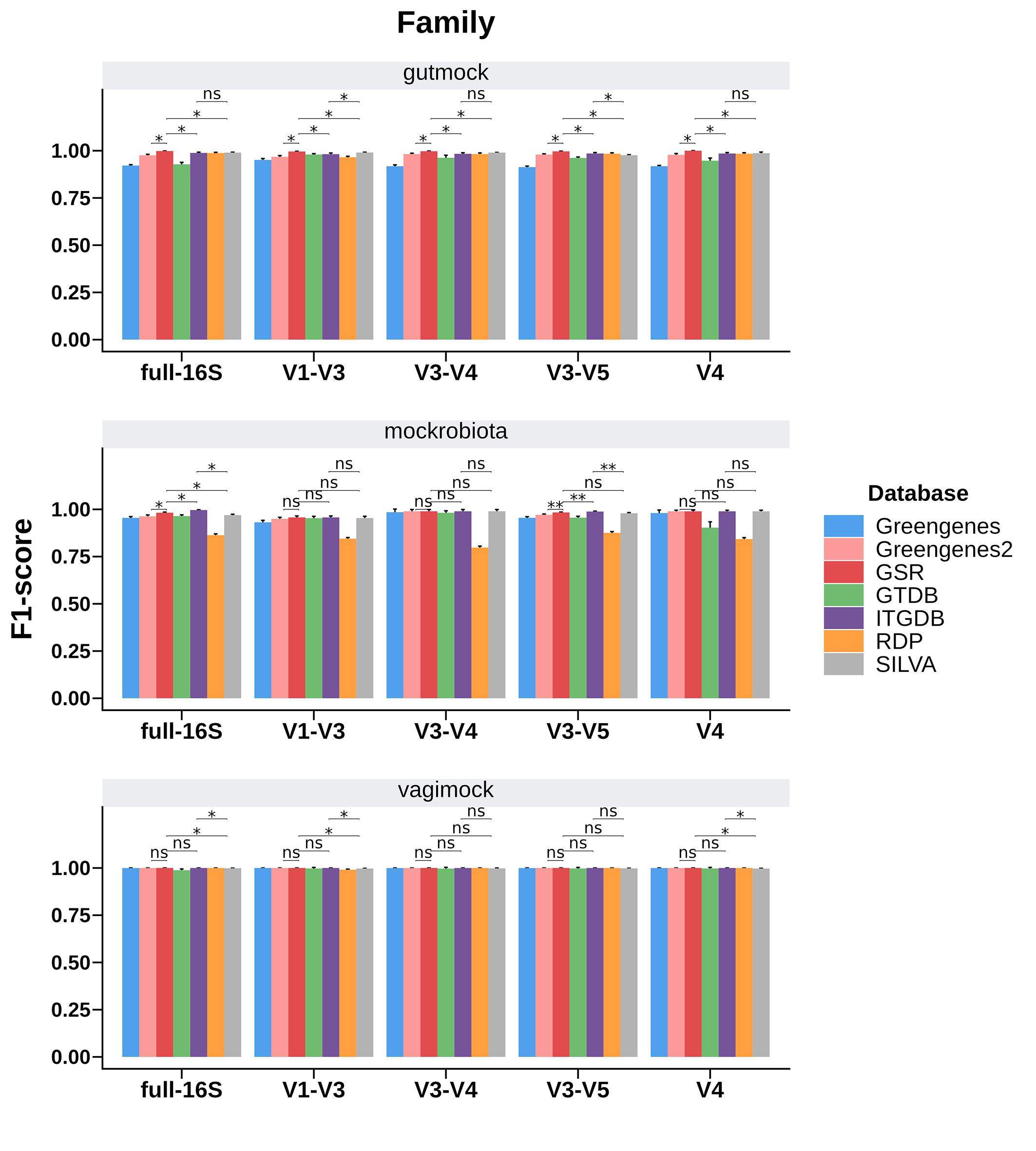

Supplement: Fig. S2 — Database benchmarking at the family level using validation metrics. [file msystems.00950-23-s0002.tiff]

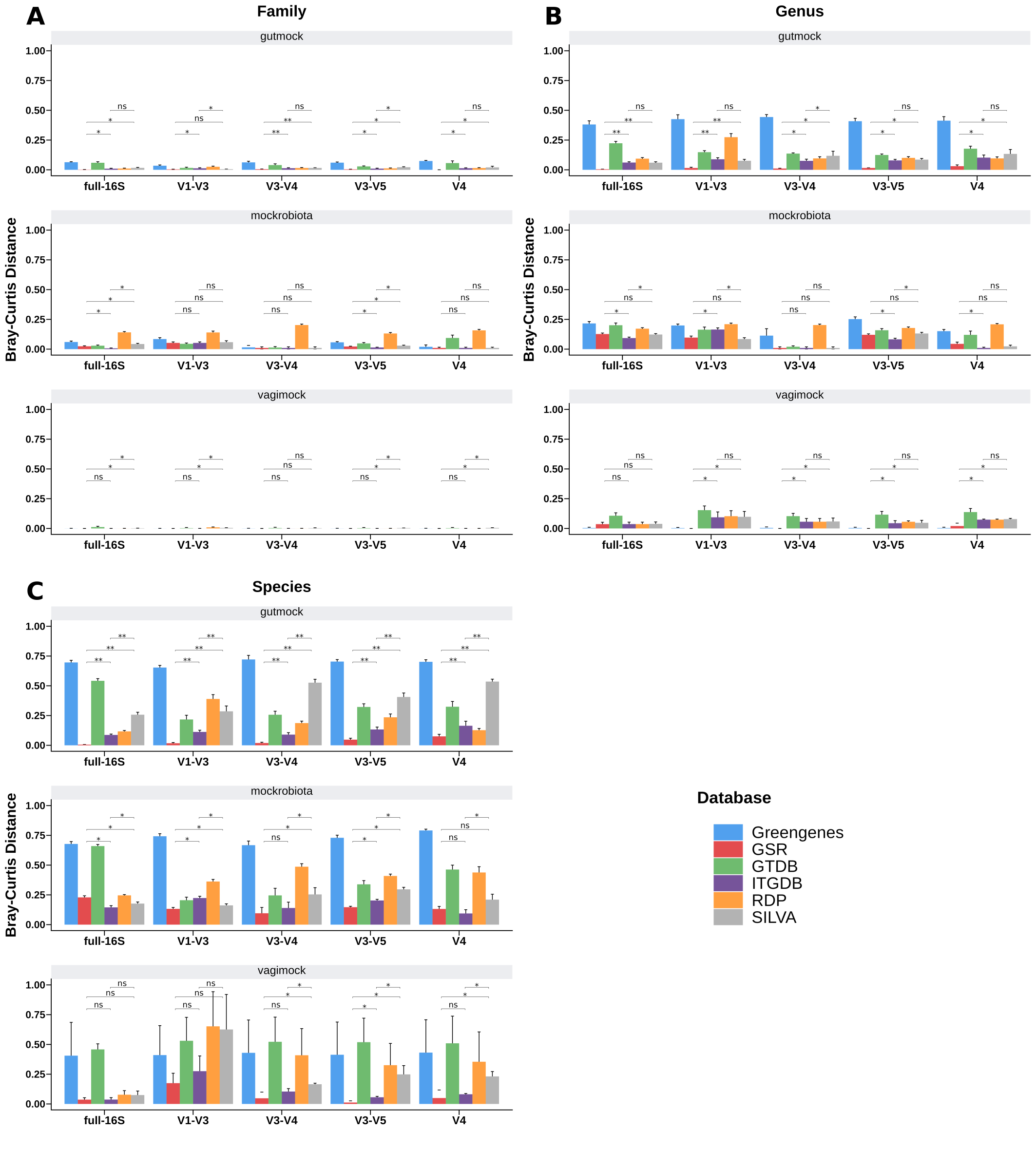

Supplement: Fig. S3 — Database benchmarking using Bray-Curtis distances between expected and observed composition at family, genus, and species levels. [file msystems.00950-23-s0003.tiff]

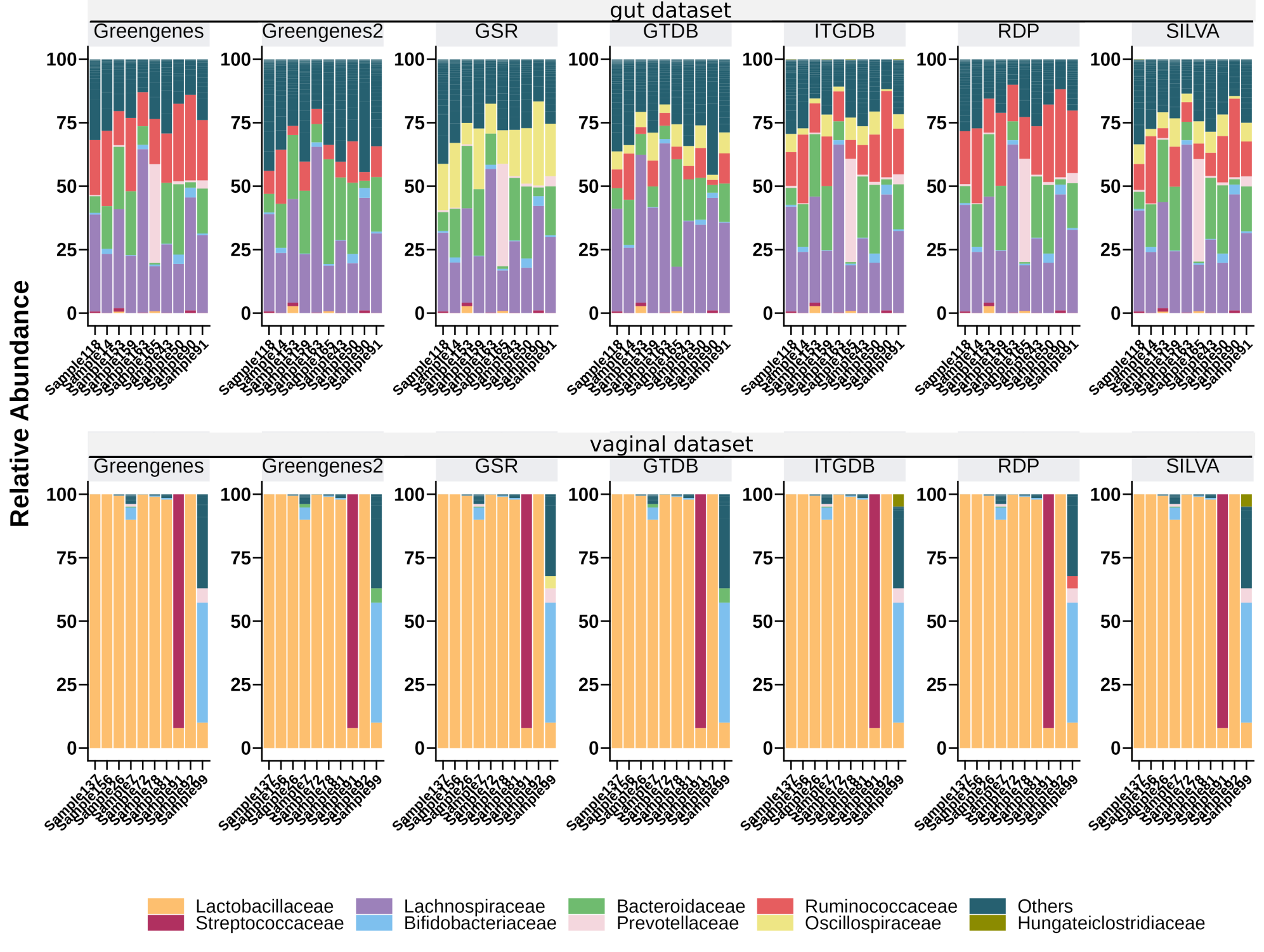

Supplement: Fig. S4 — Relative abundance of gut and vaginal samples at the family level. [file msystems.00950-23-s0004.tiff]
