# Supplementary material for: GSR-DB: a manually curated and optimized taxonomical database for 16S rRNA amplicon analysis
Source: mSystems. 2024 Jan 8;9(2):e00950-23. doi: 10.1128/msystems.00950-23 (PMC10946287; doi:10.1128/msystems.00950-23)
Supplement: Supplemental Material — Databases. [file msystems.00950-23-s0005.docx]

# Supplementary materials

**Current 16S databases**

- **The SILVA** database encompasses both Archaea and Bacteria for both SSU and LSU, for 16S and 23S. It also includes Eukaryota and Virus. This resource was established using sequences from the EMBL-Bank, with rRNA sequences predicted using Hidden Markov Models (SSU and LSU). Taxonomic assignments were derived through guide trees and supplemented by LPSN (List of Prokaryotic Names with Standing in Nomenclature). The construction of the phylogenetic tree involved employing SINA for multiple sequence alignment (MSA). In the case of SSU, Non-redundant versions (NR) are available at both 99% and 98% identity thresholds using UCLUST. The longest sequence within each cluster was retained.
- **The Greengenes** database encompasses Archaea and Bacteria 16S SSU rRNA genes and was constructed using sequences from NCBI. The phylogenetic tree was constructed using the NAST aligner, a MSA tool. Taxonomy nomenclature was compiled from different sources, where available, including the NCBI taxonomy.
- **The RDP** database includes Archaea and Bacteria SSU rrRNA, as well as fungal LSU rRNA (ITS and 28S rRNA). All subdatabases within RDP are included in the RDP classifier. This database was primarily constructed using sequences from the International Nucleotide Sequence Database Collaboration (INSDC). Taxonomy nomenclature updates were integrated using a combination of LPSN and tree based methods. The MSA was facilitated using the Infernal 1.1. aligner.
- **The GTDB** database includes metagenome-assembled genomes (MAGs) of Archaea and Bacteria sourced from the NCBI Assembly Database, which is also part of the INSDC. Taxonomy nomenclature used NCBI taxonomy, complemented by LSPN and a tree-based methodology. This database features a version specific to 16S rRNA. The construction of the phylogenetic tree involves utilizing a MSA through the FastTree tool (WAG Model). Identification of 16S sequences within genomes is achieved through nhmmer, utilizing the 16S rRNA model (RF00177) derived from the RFAM database. Sequences with a length greater than 200 bp and an E-value less than 1e-06 are reported.
- **The ITGDB** database is an integrated compilation that draws from SILVA, RDP, and Greengenes as its primary sources. Integration of these databases is achieved through a Taxonomy-based ITGDB integration method. Taxonomy nomenclature serves as an amalgamation of the three aforementioned databases.
- **The Greengenes2** database includes sequences from both Archaea and Bacteria comprising genomes and 16S sequences. Sequences are drawn from an extensive array of resources, including Web of Life 2 (WoL2), Earth Microbiome Project 500 (EMP500), Living Tree Project (LTP), GTDB, Qiita, and SILVA. Taxonomy nomenclature is established based on GTDB (tax2tree) and complemented by LTP. The construction of the phylogenetic tree involves amalgamating data from WoL2, EMP500, LTP and GTDB to form the foundational structure, facilitated by uDance. Further expansion of the tree involves the incorporation of Qiita and SILVA sequences using DEPP method for placement.
- **Metasquare** database is an integrated compilation that combines data from SILVA, Greengenes, RDP, EzBioCloud, HOMD, and the work by Pasolli, E. et al. This database encompasses 16S rRNA sequences from both Archaea and Bacteria. The integration process is based on sequence comparison, where entries that are identical or substrings of existing entries are excluded. Taxonomy nomenclature from all databases is harmonized to align with Greengenes format.
