# Supplementary material for: GSR-DB: a manually curated and optimized taxonomical database for 16S rRNA amplicon analysis
Source: mSystems. 2024 Jan 8;9(2):e00950-23. doi: 10.1128/msystems.00950-23 (PMC10946287; doi:10.1128/msystems.00950-23)
Supplement: Supplemental Legends — Legends for supplemental tables and figures. [file msystems.00950-23-s0006.docx]

TABLES

Supplementary Table 1. **Abundance and Relative abundance of the artificial mock communities at the species level.**

Supplementary Table 2. **N-gram-range and confidence threshold benchmarking at family level.** F1-score, precision and recall are shown for each database, evaluated region and validation datasets. The values are the average of the five metagenomic samples. Bold represents the highest value.

Supplementary Table 3. **N-gram-range and confidence threshold benchmarking at genus level.** F1-score, precision and recall are shown for each database, evaluated region and validation datasets. The values are the average of the five metagenomic samples. Bold represents the highest value.

Supplementary Table 4. **N-gram-range and confidence threshold benchmarking at species level.** F1-score, precision and recall are shown for each database, evaluated region and validation datasets. The values are the average of the five metagenomic samples. Bold represents the highest value.

Supplementary Table 5**.** **Database benchmarking at family levels using validation metrics.** F1-score, precision and recall are shown for each database in all the evaluated regions and datasets. Values are the average of the five metagenomic samples. Bold values are the highest and underlined values are the second highest.

Supplementary Table 6**.** **Database benchmarking at genus levels using validation metrics.** F1-score, precision and recall are shown for each database in all the evaluated regions and datasets. Values are the average of the five metagenomic samples. Bold values are the highest and underlined values are the second highest.

Supplementary Table 7**.** **Database benchmarking at species levels using validation metrics.** F1-score, precision and recall are shown for each database in all the evaluated regions and datasets. Values are the average of the five metagenomic samples. Bold values are the highest and underlined values are the second highest.

Supplementary Table 8. **Database benchmarking using Bray-Curtis distances between the expected and observed composition at family, genus and species level.** F1-score, precision and recall are shown for each database in all the evaluated regions and datasets at the family, genus and species levels. Values are the average of the five metagenomic samples. Bold values are the highest and underlined values are the second highest.

FIGURES

Supplementary Figure S1. **Visualization of the phylogenetic tree for GSR-DB, rendered using Empress.** Tip color indicates the source database of the sequences. The outer bar represents the top 6 phyla.

Supplementary Figure S2. **Database benchmarking at the family level using validation metrics.** The mean F1-score across the five metagenomic samples is shown for each evaluated region and dataset. Error bars are the standard deviation. Database benchmarking results at genus and species level are available in Figure 2C and 2D. Precision and recall metrics are available in Supplementary Tables 5, 6, and 7 for family, genus, and species levels, respectively. Wilcoxon test was conducted between F1-scores of Greengenes2, GSR, ITGDB, and SILVA databases. ns=not significant; * = padj < 0.05; **= padj < 0.01; ***= padj < 0.001.

Supplementary Figure S3. **Database benchmarking using Bray-Curtis distances between expected and observed composition at family (A), genus (B), and species (C) levels.** The mean Bray-Curtis distance across the five metagenomic samples is shown for each evaluated region and dataset. Error bars are the standard deviations. Data is available in Supplementary Table 8. Wilcoxon test was conducted between F1-scores of Greengenes2, GSR, ITGDB, and SILVA databases. ns=not significant; * = padj < 0.05; **= padj < 0.01; ***= padj < 0.001.

Supplementary Figure S4. **Relative abundance of gut and vaginal samples at the family level.** Only relevant taxa are displayed. The remaining taxa are included in the label ‘Others.’ Phylum and order levels are available in Figure 4.
