# Supplementary material for: GSR-DB: a manually curated and optimized taxonomical database for 16S rRNA amplicon analysis
Source: mSystems. 2024 Jan 8;9(2):e00950-23. doi: 10.1128/msystems.00950-23 (PMC10946287; doi:10.1128/msystems.00950-23)
Supplement: Supplemental Tables — Tables S1 to S8. [file msystems.00950-23-s0007.pdf]

Supplementary Table 1: Abundance and Relative abundance of the artificial mock communities at the species level.

Species	mockname	**Abundance**
Acidaminococcus_fermentans	gutmock	57.0 (0.03%)
Acidaminococcus_fermentans-intestini	gutmock	330.0 (0.15%)
Acidaminococcus_intestini	gutmock	930.0 (0.41%)
Acutalibacter_muris	gutmock	9.0 (0.00%)
Aestuariispira_insulae	gutmock	172.0 (0.08%)
Akkermansia_muciniphila	gutmock	2812.0 (1.25%)
Alistipes_communis	gutmock	780.0 (0.35%)
Alistipes_finegoldii	gutmock	1005.0 (0.45%)
Alistipes_ihumii	gutmock	480.0 (0.21%)
Alistipes_indistinctus	gutmock	117.0 (0.05%)
Alistipes_inops	gutmock	630.0 (0.28%)
Alistipes_onderdonkii	gutmock	4060.0 (1.81%)
Alistipes_putredinis	gutmock	630.0 (0.28%)
Alistipes_shahii	gutmock	685.0 (0.30%)
Allisonella_histaminiformans	gutmock	99.0 (0.04%)
Alloprevotella_rava	gutmock	30.0 (0.01%)
Alloscardovia_omnicolens	gutmock	54.0 (0.02%)
Alteracholeplasma_parvum	gutmock	52.0 (0.02%)
Alterileibacterium_massiliense	gutmock	175.0 (0.08%)
Anaerobutyricum_soehngenii	gutmock	87.0 (0.04%)
Anaerobutyricum_soehngenii:[Eubacterium]_hallii	gutmock	681.0 (0.30%)
Anaerococcus_obesiensis	gutmock	8.0 (0.00%)
Anaerococcus_obesiensis-vaginalis	gutmock	113.0 (0.05%)
Anaerococcus_prevotii	gutmock	87.0 (0.04%)
Anaerococcus_provencensis	gutmock	16.0 (0.01%)
Anaerococcus_vaginalis	gutmock	708.0 (0.32%)
Anaeroglobus_geminatus	gutmock	37.0 (0.02%)
Anaeromassilibacillus_senegalensis	gutmock	400.0 (0.18%)
Anaerosporobacter_mobilis	gutmock	306.0 (0.14%)
Anaerostipes_caccae	gutmock	140.0 (0.06%)
Anaerostipes_hadrus	gutmock	4656.0 (2.07%)
Anaerotignum_aminivorans	gutmock	104.0 (0.05%)
Anaerotignum_faecicola	gutmock	640.0 (0.28%)
Anaerotignum_lactatifermentans	gutmock	819.0 (0.36%)
Bacilliculturomica_massiliensis	gutmock	18.0 (0.01%)
Bacteroidales_oral	gutmock	52.0 (0.02%)
Bacteroides_caccae	gutmock	2661.0 (1.18%)
Bacteroides_caccae-stercoris:Phocaeicola_dorei	gutmock	653.0 (0.29%)
Bacteroides_clarus	gutmock	240.0 (0.11%)
Bacteroides_eggerthii	gutmock	398.0 (0.18%)
Bacteroides_finegoldii	gutmock	145.0 (0.06%)
Bacteroides_intestinalis	gutmock	142.0 (0.06%)
Bacteroides_kribbi	gutmock	869.0 (0.39%)
Bacteroides_kribbi-xylanisolvens	gutmock	352.0 (0.16%)
Bacteroides_rodentium	gutmock	905.0 (0.40%)
Bacteroides_salyersiae	gutmock	332.0 (0.15%)
Bacteroides_stercoris	gutmock	2073.0 (0.92%)
Bacteroides_xylanisolvens	gutmock	24.0 (0.01%)
Barnesiella_intestinihominis	gutmock	1295.0 (0.58%)
Bifidobacterium_bifidum	gutmock	142.0 (0.06%)
Bifidobacterium_dentium	gutmock	889.0 (0.40%)
Bilophila_wadsworthia	gutmock	1295.0 (0.58%)
Blautia_caecimuris	gutmock	278.0 (0.12%)
Blautia_caecimuris-glucerasea-producta	gutmock	133.0 (0.06%)
Blautia_caecimuris-producta	gutmock	146.0 (0.06%)
Blautia_coccoides	gutmock	29.0 (0.01%)
Blautia_coccoides-producta	gutmock	8.0 (0.00%)
Blautia_faecis	gutmock	2388.0 (1.06%)
Blautia_glucerasea	gutmock	72.0 (0.03%)
Blautia_hydrogenotrophica	gutmock	82.0 (0.04%)
Blautia_luti	gutmock	1557.0 (0.69%)
Blautia_luti-obeum-wexlerae	gutmock	5109.0 (2.27%)

Continued on next page

Supplementary Table 1: Abundance and Relative abundance of the artificial mock communities at the species level.

Species	mockname	**Abundance**
Blautia_obeum	gutmock	2173.0 (0.97%)
Blautia_producta	gutmock	78.0 (0.03%)
Blautia_schinkii	gutmock	1293.0 (0.58%)
Blautia_wexlerae	gutmock	4814.0 (2.14%)
Bulleidia_p-1630-c5	gutmock	18.0 (0.01%)
Butyricicoccus_porcorum	gutmock	64.0 (0.03%)
Butyricicoccus_pullicaecorum	gutmock	160.0 (0.07%)
Butyricimonas_faecihominis	gutmock	150.0 (0.07%)
Butyricimonas_faecihominis-paravirosa	gutmock	77.0 (0.03%)
Butyricimonas_paravirosa	gutmock	72.0 (0.03%)
Butyricimonas_virosa	gutmock	158.0 (0.07%)
Butyrivibrio_crossotus	gutmock	140.0 (0.06%)
Caecibacterium_sporoformans	gutmock	48.0 (0.02%)
Campylobacter_concisus	gutmock	29.0 (0.01%)
Campylobacter_gracilis	gutmock	20.0 (0.01%)
Campylobacter_hominis	gutmock	555.0 (0.25%)
Campylobacter_ureolyticus	gutmock	94.0 (0.04%)
Caproicibacter_fermentans	gutmock	35.0 (0.02%)
Caproiciproducens_galactitolivorans	gutmock	636.0 (0.28%)
Catenibacterium_mitsuokai	gutmock	299.0 (0.13%)
Cellulosilyticum_ruminicola	gutmock	15.0 (0.01%)
Cloacibacillus_porcorum	gutmock	70.0 (0.03%)
Clostridium_aminobutyricum	gutmock	48.0 (0.02%)
Clostridium_paraputrificum	gutmock	129.0 (0.06%)
Clostridium_tarantellae	gutmock	167.0 (0.07%)
Colidextribacter_massiliensis	gutmock	105.0 (0.05%)
Collinsella_aerofaciens	gutmock	85.0 (0.04%)
Conservatibacter_flavescens	gutmock	858.0 (0.38%)
Coprobacillus_cateniformis	gutmock	286.0 (0.13%)
Coprobacter_fastidiosus	gutmock	318.0 (0.14%)
Coprobacter_secundus	gutmock	118.0 (0.05%)
Coprococcus_catus	gutmock	1026.0 (0.46%)
Coprococcus_comes	gutmock	2191.0 (0.97%)
Coprococcus_eutactus	gutmock	2173.0 (0.97%)
Corynebacterium_pyruviciproducens	gutmock	20.0 (0.01%)
Corynebacterium_tuberculostearicum	gutmock	20.0 (0.01%)
Cuneatibacter_caecimuris	gutmock	224.0 (0.10%)
Desulfovibrio_D168	gutmock	379.0 (0.17%)
Desulfovibrio_fairfieldensis	gutmock	50.0 (0.02%)
Desulfovibrio_piger	gutmock	355.0 (0.16%)
Dialister_invisus	gutmock	836.0 (0.37%)
Dialister_micraerophilus	gutmock	15.0 (0.01%)
Dialister_pneumosintes	gutmock	120.0 (0.05%)
Dialister_propionicifaciens	gutmock	200.0 (0.09%)
Dialister_propionicifaciens-succinatiphilus	gutmock	137.0 (0.06%)
Dialister_succinatiphilus	gutmock	249.0 (0.11%)
Dielma_fastidiosa	gutmock	196.0 (0.09%)
Dorea_formicigenerans	gutmock	41.0 (0.02%)
Dorea_longicatena	gutmock	251.0 (0.11%)
Dorea_longicatena:[Ruminococcus]_gnavus:human_intestinal	gutmock	222.0 (0.10%)
Duodenibacillus_massiliensis	gutmock	316.0 (0.14%)
Dysosmobacter_welbionis	gutmock	1700.0 (0.76%)
Eisenbergiella_massiliensis	gutmock	64.0 (0.03%)
Eisenbergiella_massiliensis-tayi	gutmock	76.0 (0.03%)
Eisenbergiella_tayi	gutmock	218.0 (0.10%)
Erysipelatoclostridium_ramosum	gutmock	1896.0 (0.84%)
Erysipelatoclostridium_ramosum:[Clostridium]_saccharogumia	gutmock	247.0 (0.11%)
Ethanoligenens_harbinense	gutmock	90.0 (0.04%)
Eubacterium_coprostanoligenes	gutmock	2472.0 (1.10%)
Eubacterium_limosum	gutmock	27.0 (0.01%)
Eubacterium_ramulus	gutmock	675.0 (0.30%)
Eubacterium_ruminantium	gutmock	490.0 (0.22%)
Facklamia_hominis	gutmock	12.0 (0.01%)

Continued on next page

Supplementary Table 1: Abundance and Relative abundance of the artificial mock communities at the species level.

Species	mockname	**Abundance**
Faecalibacterium_prausnitzii	gutmock	12792.0 (5.69%)
Faecalibacterium_prausnitzii:Gemmiger_formicilis:Subdoligranulum_variabile	gutmock	571.0 (0.25%)
Faecalibacterium_prausnitzii:bacterium_ic1379	gutmock	19.0 (0.01%)
Faecalicatena_contorta	gutmock	18.0 (0.01%)
Faecalicoccus_acidiformans	gutmock	78.0 (0.03%)
Faecalimonas_umbilicata	gutmock	469.0 (0.21%)
Faecalitalea_cylindroides	gutmock	33.0 (0.01%)
Faecalitalea_cylindroides:Longicatena_caecimuris	gutmock	93.0 (0.04%)
Fastidiosipila_sanguinis	gutmock	25.0 (0.01%)
Fenollaria_massiliensis	gutmock	355.0 (0.16%)
Filifactor_villosus	gutmock	41.0 (0.02%)
Finegoldia_magna	gutmock	458.0 (0.20%)
Firmicutes_oral	gutmock	150.0 (0.07%)
Flavonifractor_plautii	gutmock	1840.0 (0.82%)
Flintibacter_butyricus	gutmock	944.0 (0.42%)
Fournierella_massiliensis	gutmock	450.0 (0.20%)
Frisingicoccus_caecimuris	gutmock	345.0 (0.15%)
Fusicatenibacter_saccharivorans	gutmock	2520.0 (1.12%)
Fusobacterium_gonidiaformans	gutmock	21.0 (0.01%)
Fusobacterium_gonidiaformans-necrophorum	gutmock	18.0 (0.01%)
Fusobacterium_naviforme	gutmock	90.0 (0.04%)
Fusobacterium_naviforme-simiae	gutmock	40.0 (0.02%)
Fusobacterium_necrophorum	gutmock	63.0 (0.03%)
Fusobacterium_periodonticum	gutmock	33.0 (0.01%)
Fusobacterium_simiae	gutmock	12.0 (0.01%)
Fusobacterium_ulcerans	gutmock	20.0 (0.01%)
Gemmiger_formicilis	gutmock	1048.0 (0.47%)
Gemmiger_formicilis:Subdoligranulum_variabile	gutmock	526.0 (0.23%)
Harryflintia_acetispora	gutmock	20.0 (0.01%)
Herbinix_luporum	gutmock	20.0 (0.01%)
Holdemanella_biformis	gutmock	939.0 (0.42%)
Holdemanella_biformis:[Eubacterium]_biforme	gutmock	258.0 (0.11%)
Holdemania_filiformis	gutmock	104.0 (0.05%)
Holdemania_massiliensis	gutmock	134.0 (0.06%)
Howardella_ureilytica	gutmock	250.0 (0.11%)
Hungatella_hathewayi	gutmock	694.0 (0.31%)
Hydrogeniiclostridium_mannosilyticum	gutmock	64.0 (0.03%)
Ihubacter_massiliensis	gutmock	825.0 (0.37%)
Intestinibacter_bartlettii	gutmock	1032.0 (0.46%)
Intestinimonas_massiliensis	gutmock	117.0 (0.05%)
Intestinimonas_timonensis	gutmock	9.0 (0.00%)
Lachnoclostridium_phocaeense	gutmock	90.0 (0.04%)
Lacticaseibacillus_chiayiensis	gutmock	66.0 (0.03%)
Lactobacillus_equicursoris	gutmock	16.0 (0.01%)
Lactobacillus_gasseri	gutmock	54.0 (0.02%)
Lactobacillus_iners	gutmock	9.0 (0.00%)
Lawsonella_clevelandensis	gutmock	75.0 (0.03%)
Lawsonibacter_asaccharolyticus	gutmock	680.0 (0.30%)
Ligilactobacillus_ruminis	gutmock	45.0 (0.02%)
Limosilactobacillus_fermentum	gutmock	39.0 (0.02%)
Limosilactobacillus_fermentum-mucosae	gutmock	40.0 (0.02%)
Limosilactobacillus_mucosae	gutmock	157.0 (0.07%)
Longibaculum_muris	gutmock	16.0 (0.01%)
Longicatena_caecimuris	gutmock	258.0 (0.11%)
Mailhella_massiliensis	gutmock	25.0 (0.01%)
Marseillibacter_massiliensis	gutmock	1185.0 (0.53%)
Massilimicrobiota_timonensis	gutmock	137.0 (0.06%)
Massiliprevotella_massiliensis	gutmock	237.0 (0.11%)
Mediterranea_massiliensis	gutmock	25.0 (0.01%)
Mediterraneibacter_faecis	gutmock	618.0 (0.27%)
Mediterraneibacter_faecis:[Ruminococcus]_torques	gutmock	331.0 (0.15%)
Megamonas_funiformis	gutmock	195.0 (0.09%)
Megasphaera_elsdenii	gutmock	111.0 (0.05%)

Continued on next page

Supplementary Table 1: Abundance and Relative abundance of the artificial mock communities at the species level.

Species	mockname	**Abundance**
Megasphaera_massiliensis	gutmock	164.0 (0.07%)
Megasphaera_micronuciformis	gutmock	89.0 (0.04%)
Merdimonas_faecis	gutmock	177.0 (0.08%)
Metaprevotella_massiliensis	gutmock	44.0 (0.02%)
Methanobrevibacter_smithii	gutmock	159.0 (0.07%)
Mitsuokella_jalaludinii	gutmock	40.0 (0.02%)
Mobiluncus_curtisii	gutmock	53.0 (0.02%)
Monoglobus_pectinilyticus	gutmock	1460.0 (0.65%)
Morganella_morganii	gutmock	65.0 (0.03%)
Murdochiella_asaccharolytica	gutmock	90.0 (0.04%)
Muribaculum_intestinale	gutmock	44.0 (0.02%)
Murimonas_intestini	gutmock	6.0 (0.00%)
Negativibacillus_massiliensis	gutmock	595.0 (0.26%)
Neglectibacter_timonensis	gutmock	928.0 (0.41%)
Odoribacter_laneus	gutmock	132.0 (0.06%)
Odoribacter_splanchnicus	gutmock	721.0 (0.32%)
Oscillibacter_ruminantium	gutmock	860.0 (0.38%)
Oxalobacter_formigenes	gutmock	12.0 (0.01%)
Papillibacter_cinnamivorans	gutmock	264.0 (0.12%)
Parabacteroides_chongii	gutmock	45.0 (0.02%)
Parabacteroides_distasonis	gutmock	4653.0 (2.07%)
Parabacteroides_goldsteinii	gutmock	1199.0 (0.53%)
Parabacteroides_gordonii	gutmock	77.0 (0.03%)
Parabacteroides_johnsonii	gutmock	367.0 (0.16%)
Parabacteroides_merdae	gutmock	3507.0 (1.56%)
Parafannyhessea_umbonata	gutmock	15.0 (0.01%)
Paraprevotella_clara	gutmock	1501.0 (0.67%)
Paraprevotella_xylaniphila	gutmock	41.0 (0.02%)
Parasutterella_excrementihominis	gutmock	985.0 (0.44%)
Parasutterella_secunda	gutmock	121.0 (0.05%)
Parvimonas_micra	gutmock	25.0 (0.01%)
Peptococcus_niger	gutmock	96.0 (0.04%)
Peptococcus_niger-simiae	gutmock	28.0 (0.01%)
Peptococcus_simiae	gutmock	12.0 (0.01%)
Peptoniphilus_catoniae	gutmock	12.0 (0.01%)
Peptoniphilus_coxii	gutmock	25.0 (0.01%)
Peptoniphilus_coxii-urinimassiliensis	gutmock	45.0 (0.02%)
Peptoniphilus_duerdenii	gutmock	40.0 (0.02%)
Peptoniphilus_lacrimalis	gutmock	230.0 (0.10%)
Peptoniphilus_obesi	gutmock	60.0 (0.03%)
Peptoniphilus_urinimassiliensis	gutmock	164.0 (0.07%)
Peptostreptococcus_anaerobius	gutmock	53.0 (0.02%)
Petroclostridium_xylanilyticum	gutmock	52.0 (0.02%)
Phascolarctobacterium_faecium	gutmock	1024.0 (0.46%)
Phascolarctobacterium_succinatutens	gutmock	64.0 (0.03%)
Phocaeicola_coprocola	gutmock	462.0 (0.21%)
Phocaeicola_coprophilus	gutmock	53.0 (0.02%)
Phocaeicola_dorei	gutmock	3001.0 (1.34%)
Phocaeicola_dorei-vulgatus	gutmock	5082.0 (2.26%)
Phocaeicola_massiliensis	gutmock	1008.0 (0.45%)
Phocaeicola_plebeius	gutmock	1162.0 (0.52%)
Phocaeicola_vulgatus	gutmock	26237.0 (11.67%)
Porphyromonas_asaccharolytica	gutmock	162.0 (0.07%)
Porphyromonas_asaccharolytica-uenonis	gutmock	60.0 (0.03%)
Porphyromonas_bennonis	gutmock	301.0 (0.13%)
Porphyromonas_somerae	gutmock	24.0 (0.01%)
Porphyromonas_uenonis	gutmock	18.0 (0.01%)
Prevotella_bivia	gutmock	460.0 (0.20%)
Prevotella_brunnea	gutmock	20.0 (0.01%)
Prevotella_buccalis	gutmock	502.0 (0.22%)
Prevotella_colorans	gutmock	88.0 (0.04%)
Prevotella_copri	gutmock	5742.0 (2.55%)
Prevotella_corporis	gutmock	1992.0 (0.89%)

Continued on next page

Supplementary Table 1: Abundance and Relative abundance of the artificial mock communities at the species level.

Species	mockname	Abundance
Prevotella_disiens	gutmock	98.0 (0.04%)
Prevotella_paludivivens	gutmock	34.0 (0.02%)
Prevotella_rara	gutmock	304.0 (0.14%)
Prevotella_stercorea	gutmock	553.0 (0.25%)
Prevotella_timonensis	gutmock	373.0 (0.17%)
Prevotellamassilia_timonensis	gutmock	460.0 (0.20%)
Pseudoflavonifractor_capillosus	gutmock	90.0 (0.04%)
Pyramidobacter_piscolens	gutmock	85.0 (0.04%)
Raoultibacter_massiliensis	gutmock	27.0 (0.01%)
Raoultibacter_massiliensis-timonensis	gutmock	54.0 (0.02%)
Raoultibacter_timonensis	gutmock	54.0 (0.02%)
Rarimicrobium_hominis	gutmock	44.0 (0.02%)
Robinsoniella_peoriensis	gutmock	48.0 (0.02%)
Romboutsia_timonensis	gutmock	20.0 (0.01%)
Roseburia_hominis	gutmock	414.0 (0.18%)
Roseburia_hominis-intestinalis	gutmock	2509.0 (1.12%)
Roseburia_intestinalis	gutmock	3537.0 (1.57%)
Roseburia_inulinivorans	gutmock	1503.0 (0.67%)
Rothia_mucilaginosa	gutmock	12.0 (0.01%)
Ruminococcus_bicirculans	gutmock	2925.0 (1.30%)
Ruminococcus_bromii	gutmock	6264.0 (2.79%)
Ruminococcus_callidus	gutmock	839.0 (0.37%)
Ruminococcus_champanellensis	gutmock	559.0 (0.25%)
Ruminococcus_champanellensis-flavefaciens	gutmock	157.0 (0.07%)
Ruminococcus_flavefaciens	gutmock	24.0 (0.01%)
Ruminococcus_lactaris	gutmock	720.0 (0.32%)
Ruminococcus_torques	gutmock	1098.0 (0.49%)
Ruminococcus_torques:[Ruminococcus]_torques	gutmock	732.0 (0.33%)
Ruthenibacterium_lactatiformans	gutmock	1310.0 (0.58%)
Sanguibacteroides_justesenii	gutmock	20.0 (0.01%)
Sarcina_ventriculi	gutmock	8.0 (0.00%)
Schaalia_odontolytica	gutmock	39.0 (0.02%)
Schaalia_turicensis	gutmock	20.0 (0.01%)
Sellimonas_intestinalis	gutmock	648.0 (0.29%)
Senegalimassilia_anaerobia	gutmock	24.0 (0.01%)
Slackia_isoflavoniconvertens	gutmock	86.0 (0.04%)
Sporobacter_termitidis	gutmock	2859.0 (1.27%)
Subdoligranulum_variabile	gutmock	81.0 (0.04%)
Succiniclasticum_ruminis	gutmock	74.0 (0.03%)
Sutterella_wadsworthensis	gutmock	329.0 (0.15%)
Turicibacter_sanguinis	gutmock	168.0 (0.07%)
Tyzzerella_nexilis	gutmock	546.0 (0.24%)
Unknown	gutmock	6.0 (0.00%)
Veillonella_denticariosi	gutmock	60.0 (0.03%)
Veillonella_denticariosi-rodentium	gutmock	31.0 (0.01%)
Veillonella_infantium	gutmock	430.0 (0.19%)
Veillonella_rodentium	gutmock	18.0 (0.01%)
Victivallis_vadensis	gutmock	260.0 (0.12%)
Winkia_neuii	gutmock	9.0 (0.00%)
[Clostridium]_citroniae	gutmock	12.0 (0.01%)
[Clostridium]_colinum	gutmock	150.0 (0.07%)
[Clostridium]_innocuum	gutmock	867.0 (0.39%)
[Clostridium]_methylpentosum	gutmock	40.0 (0.02%)
[Clostridium]_polysaccharolyticum	gutmock	12.0 (0.01%)
[Clostridium]_saccharogumia	gutmock	72.0 (0.03%)
[Clostridium]_scindens	gutmock	78.0 (0.03%)
[Clostridium]_scindens:[Ruminococcus]_gnavus	gutmock	126.0 (0.06%)
[Clostridium]_spiroforme	gutmock	287.0 (0.13%)
[Clostridium]_symbiosum	gutmock	975.0 (0.43%)
[Eubacterium]_biforme	gutmock	786.0 (0.35%)
[Eubacterium]_biforme:Holdemanella_biformis:Faecalitalea_cylindroides:Faecalicoccus_acidiformans	gutmock	508.0 (0.23%)
[Eubacterium]_eligens	gutmock	4064.0 (1.81%)
[Eubacterium]_hallii	gutmock	3292.0 (1.46%)

Continued on next page

Supplementary Table 1: Abundance and Relative abundance of the artificial mock communities at the species level.

Species	mockname	**Abundance**
[Eubacterium]_rectale	gutmock	6518.0 (2.90%)
[Eubacterium]_siraeum	gutmock	984.0 (0.44%)
[Ruminococcus]_gnavus	gutmock	1868.0 (0.83%)
anaerobic_digester	gutmock	575.0 (0.26%)
bacterium_ic1379	gutmock	8.0 (0.00%)
human_intestinal	gutmock	8.0 (0.00%)
Acidobacterium_capsulatum	mockrobiota	6.0 (0.15%)
Acinetobacter_baumannii	mockrobiota	27.0 (0.68%)
Actinomyces_odontolyticus	mockrobiota	15.0 (0.38%)
Akkermansia_muciniphila	mockrobiota	23.0 (0.58%)
Archaeoglobus_fulgidus	mockrobiota	10.0 (0.25%)
Bacillus_cereus	mockrobiota	107.0 (2.68%)
Bacillus_subtilis	mockrobiota	15.0 (0.38%)
Bacteroides_cellulosilyticus	mockrobiota	28.0 (0.70%)
Bacteroides_eggerthii	mockrobiota	9.0 (0.23%)
Bacteroides_fragilis	mockrobiota	6.0 (0.15%)
Bacteroides_ovatus	mockrobiota	106.0 (2.65%)
Bacteroides_thetaiotaomicron	mockrobiota	33.0 (0.83%)
Bacteroides_uniformis	mockrobiota	111.0 (2.78%)
Barnesiella_intestinihominis	mockrobiota	16.0 (0.40%)
Bordetella_bronchiseptica	mockrobiota	6.0 (0.15%)
Burkholderia-Caballeronia-Paraburkholderia_xenovorans	mockrobiota	12.0 (0.30%)
Caldicellulosiruptor_bescii	mockrobiota	6.0 (0.15%)
Cereibacter_sphaeroides	mockrobiota	71.0 (1.78%)
Chloroflexus_aurantiacus	mockrobiota	26.0 (0.65%)
Clostridium_beijerinckii	mockrobiota	150.0 (3.75%)
Clostridium_beijerinckii-sensu	mockrobiota	226.0 (5.65%)
Clostridium_sensu_stricto_11_acetobutylicum	mockrobiota	21.0 (0.53%)
Clostridium_sensu_stricto_1_beijerinckii	mockrobiota	110.0 (2.75%)
Clostridium_sensu_stricto_1_celatum	mockrobiota	12.0 (0.30%)
Coprococcus_comes	mockrobiota	20.0 (0.50%)
Cutibacterium_acnes	mockrobiota	71.0 (1.78%)
Deinococcus_grandis	mockrobiota	12.0 (0.30%)
Deinococcus_radiodurans	mockrobiota	95.0 (2.38%)
Desulfitobacterium_hafniense	mockrobiota	12.0 (0.30%)
Desulfovibrio_piger	mockrobiota	50.0 (1.25%)
Desulfovibrio_vulgaris	mockrobiota	12.0 (0.30%)
Dickeya_dadantii	mockrobiota	92.0 (2.30%)
Dictyoglomus_turgidum	mockrobiota	6.0 (0.15%)
Enterococcus_faecalis	mockrobiota	130.0 (3.25%)
Escherichia-Shigella_coli	mockrobiota	44.0 (1.10%)
Escherichia-Shigella_coli:Escherichia_coli	mockrobiota	68.0 (1.70%)
Escherichia_coli	mockrobiota	105.0 (2.63%)
Fusobacterium_nucleatum	mockrobiota	79.0 (1.98%)
Gemmatimonas_aurantiaca	mockrobiota	21.0 (0.53%)
Helicobacter_pylori	mockrobiota	22.0 (0.55%)
Herpetosiphon_aurantiacus	mockrobiota	32.0 (0.80%)
Howardella_ureilytica	mockrobiota	12.0 (0.30%)
Ignicoccus_hospitalis	mockrobiota	6.0 (0.15%)
Lactobacillus_gasseri	mockrobiota	24.0 (0.60%)
Leptothrix_cholodnii	mockrobiota	6.0 (0.15%)
Listeria_monocytogenes	mockrobiota	119.0 (2.98%)
Methanobrevibacter_smithii	mockrobiota	20.0 (0.50%)
Methanocaldococcus_jannaschii	mockrobiota	9.0 (0.23%)
Methanococcus_maripaludis	mockrobiota	14.0 (0.35%)
Microlunatus_phosphovorus	mockrobiota	9.0 (0.23%)
Nanoarchaeum_equitans	mockrobiota	2.0 (0.05%)
Neisseria_meningitidis	mockrobiota	22.0 (0.55%)
Nitrobacter_winogradskyi	mockrobiota	9.0 (0.23%)
Nitrosomonas_europaea	mockrobiota	21.0 (0.53%)
Parabacteroides_distasonis	mockrobiota	24.0 (0.60%)
Parabacteroides_merdae	mockrobiota	85.0 (2.13%)
Paraprevotella_clara	mockrobiota	10.0 (0.25%)

Continued on next page

Supplementary Table 1: Abundance and Relative abundance of the artificial mock communities at the species level.

Species	mockname	**Abundance**
Pelodictyon_phaeoclathratiforme	mockrobiota	43.0 (1.08%)
Pelolinea_thermophila	mockrobiota	12.0 (0.30%)
Persephonella_marina	mockrobiota	4.0 (0.10%)
Phocaeicola_massiliensis	mockrobiota	12.0 (0.30%)
Phocaeicola_vulgatus	mockrobiota	295.0 (7.38%)
Porphyromonas_gingivalis	mockrobiota	90.0 (2.25%)
Prevotella_buccalis	mockrobiota	6.0 (0.15%)
Prevotella_copri	mockrobiota	37.0 (0.93%)
Pseudomonas_aeruginosa	mockrobiota	76.0 (1.90%)
Pseudomonas_putida	mockrobiota	12.0 (0.30%)
Pyrobaculum_aerophilum	mockrobiota	3.0 (0.08%)
Pyrobaculum_calidifontis	mockrobiota	6.0 (0.15%)
Pyrococcus_horikoshii	mockrobiota	8.0 (0.20%)
Rhodopirellula_baltica	mockrobiota	8.0 (0.20%)
Roseburia_inulinivorans	mockrobiota	6.0 (0.15%)
Ruegeria_pomeroyi	mockrobiota	12.0 (0.30%)
Salinispora_arenicola	mockrobiota	9.0 (0.23%)
Salinispora_arenicola-tropica	mockrobiota	2.0 (0.05%)
Salinispora_tropica	mockrobiota	12.0 (0.30%)
Shewanella_baltica	mockrobiota	290.0 (7.25%)
Staphylococcus_aureus	mockrobiota	65.0 (1.63%)
Staphylococcus_aureus-epidermidis	mockrobiota	118.0 (2.95%)
Staphylococcus_epidermidis	mockrobiota	102.0 (2.55%)
Streptococcus_agalactiae	mockrobiota	18.0 (0.45%)
Streptococcus_mutans	mockrobiota	73.0 (1.83%)
Streptococcus_pneumoniae	mockrobiota	23.0 (0.58%)
Sulfurihydrogenibium_yellowstonense	mockrobiota	29.0 (0.73%)
Sulfurisphaera_tokodaii	mockrobiota	4.0 (0.10%)
Thermotoga_neapolitana	mockrobiota	16.0 (0.40%)
Thermotoga_neapolitana-petrophila	mockrobiota	6.0 (0.15%)
Thermotoga_petrophila	mockrobiota	12.0 (0.30%)
Thermus_thermophilus	mockrobiota	6.0 (0.15%)
Treponema_bryantii	mockrobiota	30.0 (0.75%)
Treponema_denticola	mockrobiota	39.0 (0.98%)
Treponema_vincentii	mockrobiota	6.0 (0.15%)
Zymomonas_mobilis	mockrobiota	12.0 (0.30%)
[Clostridium]_cocleatum	mockrobiota	6.0 (0.15%)
[Clostridium]_methylpentosum	mockrobiota	14.0 (0.35%)
[Clostridium]_methylpentosum_group_methylpentosum	mockrobiota	22.0 (0.55%)
[Ruminococcus]_gnavus	mockrobiota	16.0 (0.40%)
[Ruminococcus]_gnavus_group_gnavus	mockrobiota	24.0 (0.60%)
Aerococcus_christensenii	vagimock	74.0 (0.03%)
Alloscardovia_omnicolens	vagimock	450.0 (0.19%)
Anaerococcus_obesiensis	vagimock	4.0 (0.00%)
Anaerococcus_prevotii	vagimock	25.0 (0.01%)
Anaeroglobus_geminatus	vagimock	124.0 (0.05%)
Bacteroides_uniformis	vagimock	21.0 (0.01%)
Bacteroides_uniformis:Phocaeicola_vulgatus	vagimock	11.0 (0.00%)
Bifidobacterium_bifidum	vagimock	6.0 (0.00%)
Bifidobacterium_dentium	vagimock	1276.0 (0.55%)
Blautia_faecis	vagimock	4.0 (0.00%)
Blautia_obeum	vagimock	6.0 (0.00%)
Clostridium_perfringens	vagimock	33.0 (0.01%)
Dialister_micraerophilus	vagimock	106.0 (0.05%)
Dialister_succinatiphilus	vagimock	28.0 (0.01%)
Eubacterium_coprostanoligenes	vagimock	28.0 (0.01%)
Faecalibacterium_prausnitzii	vagimock	66.0 (0.03%)
Fannyhessea_vaginae	vagimock	1954.0 (0.84%)
Fenollaria_massiliensis	vagimock	8.0 (0.00%)
Finegoldia_magna	vagimock	46.0 (0.02%)
Fusicatenibacter_saccharivorans	vagimock	14.0 (0.01%)
Gardnerella_vaginalis	vagimock	13085.0 (5.60%)
Howardella_ureilytica	vagimock	610.0 (0.26%)

Continued on next page

Supplementary Table 1: Abundance and Relative abundance of the artificial mock communities at the species level.

Species	mockname	**Abundance**
Lactobacillus_crispatus	vagimock	57030.0 (24.39%)
Lactobacillus_crispatus-delbrueckii	vagimock	984.0 (0.42%)
Lactobacillus_delbrueckii	vagimock	54.0 (0.02%)
Lactobacillus_gasseri	vagimock	7381.0 (3.16%)
Lactobacillus_iners	vagimock	143924.0 (61.56%)
Lactobacillus_jensenii	vagimock	4419.0 (1.89%)
Limosilactobacillus_coleohominis	vagimock	6.0 (0.00%)
Limosilactobacillus_pontis	vagimock	27.0 (0.01%)
Limosilactobacillus_vaginalis	vagimock	81.0 (0.03%)
Mediterraneibacter_faecis	vagimock	6.0 (0.00%)
Megasphaera_stantonii	vagimock	294.0 (0.13%)
Metamycoplasma_hominis	vagimock	20.0 (0.01%)
Monoglobus_pectinilyticus	vagimock	8.0 (0.00%)
Parabacteroides_distasonis	vagimock	9.0 (0.00%)
Peptoniphilus_duerdenii	vagimock	6.0 (0.00%)
Phocaeicola_vulgatus	vagimock	42.0 (0.02%)
Porphyromonas_uenonis	vagimock	16.0 (0.01%)
Prevotella_amnii	vagimock	9.0 (0.00%)
Prevotella_bivia	vagimock	606.0 (0.26%)
Prevotella_buccalis	vagimock	12.0 (0.01%)
Prevotella_colorans	vagimock	28.0 (0.01%)
Prevotella_copri	vagimock	242.0 (0.10%)
Prevotella_timonensis	vagimock	334.0 (0.14%)
Roseburia_faecis	vagimock	6.0 (0.00%)
Ruminococcus_bicirculans	vagimock	10.0 (0.00%)
Ruminococcus_bromii	vagimock	9.0 (0.00%)
Sporobacter_termitidis	vagimock	31.0 (0.01%)
Streptococcus_agalactiae	vagimock	80.0 (0.03%)
Ureaplasma_urealyticum	vagimock	90.0 (0.04%)
Winkia_neuii	vagimock	6.0 (0.00%)
[Eubacterium]_hallii	vagimock	12.0 (0.01%)
anaerobic_digester	vagimock	25.0 (0.01%)

Supplementary Table 2: N-gram-range and confidence threshold benchmarking at family level. F1-score, precision and recall are shown for each database, evaluated region and validation datasets. The values are the average of the five metagenomic samples. Bold represents the highest value.

Validation Dataset	Region	Database	Metrics	Conf. thres. N-gram	**0.5**	**0.7**	**0.9**	**0.98**	**disable**
	V1-V3	GSR	F1-score	[6,6]	**1.00**	0.99	0.99	0.99	**1.00**
				[7,7]	**1.00**	0.99	0.99	0.99	**1.00**
			Precision	[6,6]	**1.00**	**1.00**	**1.00**	**1.00**	**1.00**
				[7,7]	**1.00**	**1.00**	**1.00**	**1.00**	**1.00**
			Recall	[6,6]	**1.00**	0.99	0.99	0.99	**1.00**
				[7,7]	**1.00**	0.99	0.99	0.99	**1.00**
		GTDB	F1-score	[6,6]	0.98	0.96	0.95	0.95	0.98
				[7,7]	0.98	0.97	0.96	0.95	0.98
			Precision	[6,6]	0.99	0.98	0.99	0.99	0.98
				[7,7]	0.99	0.99	0.99	0.98	0.98
			Recall	[6,6]	0.98	0.95	0.93	0.92	0.98
				[7,7]	0.98	0.95	0.93	0.92	0.98
		Greengenes	F1-score	[6,6]	0.96	0.96	0.95	0.95	0.96
				[7,7]	0.96	0.96	0.96	0.95	0.96
			Precision	[6,6]	0.97	0.97	0.96	0.97	0.97
				[7,7]	0.97	0.97	0.97	0.97	0.97
			Recall	[6,6]	0.95	0.95	0.95	0.93	0.95
				[7,7]	0.96	0.95	0.95	0.95	0.95
		ITGDB	F1-score	[6,6]	0.95	0.89	0.83	0.77	0.98
				[7,7]	0.95	0.87	0.83	0.78	0.98
			Precision	[6,6]	0.97	0.97	0.97	0.97	0.98
				[7,7]	0.97	0.97	0.97	0.97	0.98
			Recall	[6,6]	0.95	0.85	0.75	0.68	0.98
				[7,7]	0.94	0.81	0.76	0.69	0.98
		RDP	F1-score	[6,6]	0.97	0.97	0.96	0.96	0.97
				[7,7]	0.97	0.97	0.97	0.97	0.97
			Precision	[6,6]	0.97	0.97	0.97	0.97	0.97
				[7,7]	0.97	0.97	0.97	0.97	0.97
			Recall	[6,6]	0.97	0.97	0.97	0.96	0.97
				[7,7]	0.97	0.97	0.97	0.97	0.97
		SILVA	F1-score	[6,6]	0.99	0.99	0.99	0.99	0.99
				[7,7]	0.99	0.99	0.99	0.99	0.99
			Precision	[6,6]	0.99	0.99	0.99	0.99	0.99
				[7,7]	0.99	0.99	0.99	0.99	0.99
			Recall	[6,6]	0.99	0.99	0.99	0.98	0.99
				[7,7]	0.99	0.99	0.99	0.99	0.99
	V3-V4	GSR	F1-score	[6,6]	**1.00**	**1.00**	**1.00**	0.99	**1.00**
				[7,7]	**1.00**	**1.00**	**1.00**	0.99	**1.00**
			Precision	[6,6]	**1.00**	**1.00**	**1.00**	**1.00**	**1.00**
				[7,7]	**1.00**	**1.00**	**1.00**	**1.00**	**1.00**
			Recall	[6,6]	0.99	0.99	0.99	0.99	0.99
				[7,7]	0.99	0.99	0.99	0.99	0.99
		GTDB	F1-score	[6,6]	0.95	0.93	0.93	0.89	0.96
				[7,7]	0.94	0.93	0.92	0.89	0.96
			Precision	[6,6]	0.98	0.98	0.98	0.97	0.98
				[7,7]	0.98	0.98	0.98	0.97	0.98
			Recall	[6,6]	0.94	0.91	0.91	0.84	0.96
				[7,7]	0.92	0.91	0.90	0.85	0.96
		Greengenes	F1-score	[6,6]	0.95	0.94	0.94	0.93	0.95
				[7,7]	0.95	0.95	0.94	0.94	0.95
			Precision	[6,6]	0.96	0.95	0.95	0.95	0.96
				[7,7]	0.96	0.96	0.95	0.95	0.96
			Recall	[6,6]	0.95	0.94	0.94	0.93	0.95
				[7,7]	0.95	0.95	0.94	0.94	0.95
		ITGDB	F1-score	[6,6]	0.93	0.80	0.68	0.58	0.98
				[7,7]	0.93	0.79	0.68	0.60	0.98
			Precision	[6,6]	0.97	0.95	0.96	0.92	0.99
				[7,7]	0.98	0.96	0.96	0.93	0.99
			Recall	[6,6]	0.90	0.73	0.58	0.49	0.98
				[7,7]	0.89	0.71	0.58	0.50	0.98

Continued on next page

Supplementary Table 2: N-gram-range and confidence threshold benchmarking at family level. F1-score, precision and recall are shown for each database, evaluated region and validation datasets. The values are the average of the five metagenomic samples. Bold represents the highest value.

Validation Dataset	Region	Database	Metrics	Conf. thres. N-gram	**0.5**	**0.7**	**0.9**	**0.98**	**disable**
		RDP	F1-score	[6,6]	0.98	0.98	0.98	0.98	0.98
				[7,7]	0.98	0.98	0.98	0.98	0.98
			Precision	[6,6]	0.99	0.99	0.99	0.99	0.99
				[7,7]	0.99	0.99	0.99	0.99	0.99
			Recall	[6,6]	0.98	0.98	0.98	0.98	0.98
				[7,7]	0.98	0.98	0.98	0.98	0.98
		SILVA	F1-score	[6,6]	0.99	0.98	0.97	0.95	0.99
				[7,7]	0.99	0.99	0.98	0.98	0.99
			Precision	[6,6]	**1.00**	0.99	0.98	0.96	**1.00**
				[7,7]	**1.00**	**1.00**	0.99	0.98	**1.00**
			Recall	[6,6]	0.99	0.98	0.97	0.96	0.99
				[7,7]	0.98	0.99	0.98	0.98	0.98
	V3-V5	GSR	F1-score	[6,6]	**1.00**	0.99	0.99	0.98	**1.00**
				[7,7]	**1.00**	**1.00**	**1.00**	0.98	**1.00**
			Precision	[6,6]	**1.00**	0.99	0.99	0.99	**1.00**
				[7,7]	**1.00**	**1.00**	**1.00**	0.99	**1.00**
			Recall	[6,6]	**1.00**	0.99	0.99	0.98	**1.00**
				[7,7]	**1.00**	**1.00**	**1.00**	0.98	**1.00**
		GTDB	F1-score	[6,6]	0.96	0.95	0.93	0.90	0.97
				[7,7]	0.95	0.94	0.93	0.89	0.96
			Precision	[6,6]	0.98	0.98	0.98	0.96	0.98
				[7,7]	0.98	0.98	0.98	0.98	0.97
			Recall	[6,6]	0.95	0.93	0.90	0.86	0.97
				[7,7]	0.94	0.92	0.90	0.84	0.96
		Greengenes	F1-score	[6,6]	0.95	0.95	0.94	0.94	0.95
				[7,7]	0.95	0.95	0.95	0.94	0.95
			Precision	[6,6]	0.95	0.96	0.95	0.94	0.95
				[7,7]	0.96	0.95	0.96	0.95	0.95
			Recall	[6,6]	0.95	0.95	0.94	0.94	0.95
				[7,7]	0.95	0.95	0.95	0.94	0.94
		ITGDB	F1-score	[6,6]	0.94	0.85	0.77	0.63	0.99
				[7,7]	0.94	0.83	0.77	0.64	0.99
			Precision	[6,6]	0.96	0.97	0.97	0.96	0.99
				[7,7]	0.96	0.97	0.97	0.97	0.99
			Recall	[6,6]	0.93	0.79	0.68	0.51	0.99
				[7,7]	0.92	0.75	0.67	0.52	0.99
		RDP	F1-score	[6,6]	0.99	0.99	0.97	0.97	0.98
				[7,7]	0.98	0.99	0.99	0.97	0.98
			Precision	[6,6]	0.99	0.99	0.98	0.97	0.99
				[7,7]	0.99	0.99	0.99	0.98	0.99
			Recall	[6,6]	0.98	0.98	0.98	0.97	0.98
				[7,7]	0.98	0.99	0.99	0.97	0.98
		SILVA	F1-score	[6,6]	0.98	0.98	0.97	0.96	0.98
				[7,7]	0.98	0.98	0.98	0.97	0.98
			Precision	[6,6]	0.98	0.98	0.97	0.98	0.98
				[7,7]	0.98	0.98	0.98	0.97	0.98
			Recall	[6,6]	0.98	0.98	0.97	0.97	0.98
				[7,7]	0.98	0.98	0.98	0.98	0.97
		GSR	F1-score	[6,6]	**1.00**	**1.00**	0.99	0.98	**1.00**
				[7,7]	**1.00**	**1.00**	0.99	0.99	**1.00**
			Precision	[6,6]	**1.00**	**1.00**	**1.00**	**1.00**	**1.00**
				[7,7]	**1.00**	**1.00**	**1.00**	**1.00**	**1.00**
			Recall	[6,6]	**1.00**	**1.00**	0.99	0.97	**1.00**
				[7,7]	**1.00**	**1.00**	0.99	0.98	**1.00**
		GTDB	F1-score	[6,6]	0.92	0.92	0.88	0.73	0.95
				[7,7]	0.92	0.91	0.89	0.77	0.95
			Precision	[6,6]	0.98	0.98	0.96	0.93	0.98
				[7,7]	0.97	0.97	0.96	0.94	0.97
			Recall	[6,6]	0.89	0.88	0.84	0.63	0.94
				[7,7]	0.89	0.88	0.85	0.67	0.93
		Greengenes	F1-score	[6,6]	0.95	0.94	0.95	0.92	0.95

Continued on next page

Supplementary Table 2: N-gram-range and confidence threshold benchmarking at family level. F1-score, precision and recall are shown for each database, evaluated region and validation datasets. The values are the average of the five metagenomic samples. Bold represents the highest value.

Validation Dataset	Region	Database	Metrics	Conf. thres. / N-gram	**0.5**	**0.7**	**0.9**	**0.98**	**disable**
				[7,7]	0.95	0.95	0.94	0.94	0.95
			Precision	[6,6]	0.96	0.96	0.96	0.95	0.96
				[7,7]	0.96	0.96	0.96	0.95	0.96
			Recall	[6,6]	0.95	0.94	0.94	0.92	0.95
				[7,7]	0.94	0.95	0.94	0.94	0.95
		ITGDB	F1-score	[6,6]	0.93	0.76	0.63	0.47	0.99
				[7,7]	0.94	0.76	0.63	0.50	0.99
			Precision	[6,6]	0.98	0.97	0.94	0.93	0.99
				[7,7]	0.98	0.97	0.94	0.93	0.99
			Recall	[6,6]	0.91	0.67	0.52	0.35	0.98
				[7,7]	0.91	0.68	0.52	0.37	0.98
		RDP	F1-score	[6,6]	0.98	0.98	0.98	0.97	0.98
				[7,7]	0.98	0.98	0.98	0.98	0.98
			Precision	[6,6]	0.99	0.99	0.99	0.99	0.99
				[7,7]	0.99	0.99	0.99	0.99	0.99
			Recall	[6,6]	0.98	0.98	0.98	0.96	0.98
				[7,7]	0.98	0.98	0.98	0.98	0.98
		SILVA	F1-score	[6,6]	0.99	0.99	0.98	0.93	0.99
				[7,7]	**1.00**	0.99	0.98	0.96	0.99
			Precision	[6,6]	0.99	0.99	0.99	0.98	**1.00**
				[7,7]	**1.00**	**1.00**	0.99	0.98	**1.00**
			Recall	[6,6]	0.99	0.99	0.98	0.91	0.99
				[7,7]	0.99	0.99	0.98	0.95	0.98
	full-16S	GSR	F1-score	[6,6]	**1.00**	**1.00**	**1.00**	0.99	**1.00**
				[7,7]	**1.00**	**1.00**	**1.00**	**1.00**	**1.00**
			Precision	[6,6]	**1.00**	**1.00**	**1.00**	0.99	**1.00**
				[7,7]	**1.00**	**1.00**	**1.00**	**1.00**	**1.00**
			Recall	[6,6]	**1.00**	**1.00**	**1.00**	0.98	**1.00**
				[7,7]	**1.00**	**1.00**	**1.00**	**1.00**	**1.00**
		GTDB	F1-score	[6,6]	0.92	0.85	0.82	0.78	0.92
				[7,7]	0.93	0.92	0.91	0.91	0.93
			Precision	[6,6]	0.93	0.96	0.94	0.94	0.93
				[7,7]	0.95	0.95	0.95	0.96	0.95
			Recall	[6,6]	0.91	0.81	0.78	0.73	0.92
				[7,7]	0.92	0.91	0.89	0.88	0.92
		Greengenes	F1-score	[6,6]	0.94	0.94	0.94	0.93	0.94
				[7,7]	0.95	0.95	0.94	0.94	0.95
			Precision	[6,6]	0.96	0.95	0.96	0.96	0.96
				[7,7]	0.96	0.96	0.96	0.96	0.96
			Recall	[6,6]	0.94	0.93	0.94	0.91	0.94
				[7,7]	0.94	0.94	0.94	0.94	0.94
		ITGDB	F1-score	[6,6]	0.97	0.91	0.83	0.71	0.99
				[7,7]	0.97	0.92	0.83	0.74	0.99
			Precision	[6,6]	0.98	0.98	0.98	0.97	0.99
				[7,7]	0.98	0.99	0.99	0.98	0.99
			Recall	[6,6]	0.96	0.86	0.75	0.60	0.99
				[7,7]	0.96	0.87	0.74	0.63	0.99
		RDP	F1-score	[6,6]	0.99	0.99	0.99	0.99	0.99
				[7,7]	0.99	0.99	0.99	0.99	0.99
			Precision	[6,6]	0.99	0.99	0.99	0.99	0.99
				[7,7]	0.99	0.99	0.99	0.99	0.99
			Recall	[6,6]	0.99	0.99	0.99	0.99	0.99
				[7,7]	0.99	0.99	0.99	0.99	0.99
		SILVA	F1-score	[6,6]	0.98	0.98	0.98	0.96	0.99
				[7,7]	0.99	0.99	0.99	0.99	0.99
			Precision	[6,6]	0.99	0.99	0.99	0.97	**1.00**
				[7,7]	**1.00**	**1.00**	**1.00**	**1.00**	**1.00**
			Recall	[6,6]	0.98	0.98	0.98	0.96	0.98
				[7,7]	0.99	0.99	0.99	0.99	0.98
		GSR	F1-score	[6,6]	0.95	0.95	0.95	0.95	0.97
				[7,7]	0.96	0.95	0.95	0.95	0.96

Continued on next page

Supplementary Table 2: N-gram-range and confidence threshold benchmarking at family level. F1-score, precision and recall are shown for each database, evaluated region and validation datasets. The values are the average of the five metagenomic samples. Bold represents the highest value.

Validation Dataset	Region	Database	Metrics	Conf. thres. / N-gram	**0.5**	**0.7**	**0.9**	**0.98**	**disable**
			Precision	[6,6]	0.99	0.99	0.99	0.99	0.99
				[7,7]	0.99	0.99	0.99	0.99	0.99
			Recall	[6,6]	0.93	0.93	0.93	0.93	0.95
				[7,7]	0.95	0.94	0.93	0.93	0.95
		GTDB	F1-score	[6,6]	0.92	0.91	0.90	0.90	0.96
				[7,7]	0.93	0.92	0.90	0.90	0.95
			Precision	[6,6]	0.97	0.96	0.95	0.96	0.97
				[7,7]	0.97	0.97	0.96	0.95	0.97
			Recall	[6,6]	0.90	0.89	0.88	0.88	0.95
				[7,7]	0.91	0.90	0.88	0.88	0.94
		Greengenes	F1-score	[6,6]	0.94	0.94	0.92	0.91	0.94
				[7,7]	0.94	0.94	0.93	0.92	0.94
			Precision	[6,6]	0.98	0.98	0.95	0.93	0.98
				[7,7]	0.98	0.98	0.96	0.95	0.98
			Recall	[6,6]	0.93	0.92	0.90	0.90	0.92
				[7,7]	0.92	0.92	0.91	0.90	0.92
		ITGDB	F1-score	[6,6]	0.91	0.85	0.74	0.54	0.97
				[7,7]	0.93	0.85	0.74	0.56	0.96
			Precision	[6,6]	0.95	0.94	0.90	0.65	0.99
				[7,7]	0.97	0.94	0.86	0.68	0.99
			Recall	[6,6]	0.89	0.81	0.68	0.49	0.96
				[7,7]	0.91	0.81	0.69	0.51	0.95
		RDP	F1-score	[6,6]	0.82	0.82	0.82	0.81	0.82
				[7,7]	0.85	0.84	0.82	0.82	0.84
			Precision	[6,6]	0.83	0.86	0.86	0.85	0.83
				[7,7]	0.87	0.90	0.86	0.86	0.87
			Recall	[6,6]	0.81	0.81	0.81	0.80	0.82
				[7,7]	0.84	0.83	0.81	0.81	0.84
		SILVA	F1-score	[6,6]	0.95	0.95	0.95	0.93	0.95
				[7,7]	0.95	0.95	0.95	0.94	0.95
			Precision	[6,6]	0.99	0.99	0.99	0.99	0.99
				[7,7]	0.99	0.99	0.99	0.99	0.99
			Recall	[6,6]	0.93	0.93	0.93	0.91	0.93
				[7,7]	0.94	0.94	0.93	0.92	0.94
	V3-V4	GSR	F1-score	[6,6]	0.99	0.99	0.99	0.99	0.99
				[7,7]	0.99	0.99	0.99	0.99	0.99
			Precision	[6,6]	0.99	0.99	0.99	0.99	0.99
				[7,7]	0.99	0.99	0.99	0.99	0.99
			Recall	[6,6]	0.99	0.99	0.99	0.99	0.99
				[7,7]	0.99	0.99	0.99	0.99	0.99
		GTDB	F1-score	[6,6]	0.93	0.93	0.89	0.89	0.98
				[7,7]	0.93	0.93	0.89	0.89	0.98
			Precision	[6,6]	0.92	0.92	0.90	0.90	0.98
				[7,7]	0.92	0.92	0.90	0.90	0.98
			Recall	[6,6]	0.94	0.94	0.89	0.89	0.99
				[7,7]	0.94	0.94	0.89	0.89	0.99
		Greengenes	F1-score	[6,6]	0.98	0.98	0.94	0.94	0.99
				[7,7]	0.98	0.98	0.94	0.94	0.99
			Precision	[6,6]	0.98	0.98	0.94	0.94	0.99
				[7,7]	0.98	0.98	0.94	0.94	0.99
			Recall	[6,6]	0.99	0.99	0.94	0.94	0.99
				[7,7]	0.99	0.99	0.94	0.94	0.99
		ITGDB	F1-score	[6,6]	0.98	0.86	0.64	0.59	0.99
				[7,7]	0.98	0.86	0.64	0.53	0.99
			Precision	[6,6]	0.98	0.90	0.68	0.62	0.99
				[7,7]	0.98	0.90	0.68	0.57	0.99
			Recall	[6,6]	0.98	0.84	0.62	0.56	0.99
				[7,7]	0.98	0.84	0.62	0.51	0.99
		RDP	F1-score	[6,6]	0.80	0.80	0.80	0.80	0.80
				[7,7]	0.80	0.80	0.80	0.80	0.80
			Precision	[6,6]	0.80	0.80	0.80	0.80	0.80

Continued on next page

Supplementary Table 2: N-gram-range and confidence threshold benchmarking at family level. F1-score, precision and recall are shown for each database, evaluated region and validation datasets. The values are the average of the five metagenomic samples. Bold represents the highest value.

Validation Dataset	Region	Database	Metrics	Conf. thres. / N-gram	**0.5**	**0.7**	**0.9**	**0.98**	**disable**
				[7,7]	0.80	0.80	0.80	0.80	0.80
			Recall	[6,6]	0.80	0.80	0.80	0.80	0.80
				[7,7]	0.80	0.80	0.80	0.80	0.80
		SILVA	F1-score	[6,6]	0.99	0.99	0.99	**1.00**	0.99
				[7,7]	0.99	0.99	0.99	0.99	0.99
			Precision	[6,6]	0.99	0.99	0.99	**1.00**	0.99
				[7,7]	0.99	0.99	0.99	0.99	0.99
			Recall	[6,6]	0.99	0.99	0.99	**1.00**	0.99
				[7,7]	0.99	0.99	0.99	0.99	0.99
	V3-V5	GSR	F1-score	[6,6]	0.97	0.97	0.96	0.96	0.98
				[7,7]	0.97	0.97	0.96	0.96	0.98
			Precision	[6,6]	0.99	0.99	0.99	0.98	0.99
				[7,7]	0.99	0.99	0.99	0.98	0.99
			Recall	[6,6]	0.96	0.96	0.95	0.94	0.97
				[7,7]	0.96	0.96	0.95	0.94	0.98
		GTDB	F1-score	[6,6]	0.94	0.91	0.91	0.89	0.97
				[7,7]	0.93	0.92	0.91	0.90	0.96
			Precision	[6,6]	0.97	0.96	0.96	0.95	0.97
				[7,7]	0.97	0.96	0.95	0.95	0.97
			Recall	[6,6]	0.93	0.90	0.90	0.88	0.97
				[7,7]	0.92	0.91	0.90	0.89	0.95
		Greengenes	F1-score	[6,6]	0.96	0.95	0.93	0.88	0.96
				[7,7]	0.97	0.96	0.94	0.90	0.96
			Precision	[6,6]	0.97	0.97	0.95	0.89	0.98
				[7,7]	0.98	0.97	0.96	0.93	0.98
			Recall	[6,6]	0.95	0.93	0.92	0.87	0.94
				[7,7]	0.96	0.95	0.93	0.89	0.95
		ITGDB	F1-score	[6,6]	0.93	0.82	0.58	0.48	0.98
				[7,7]	0.93	0.80	0.59	0.48	0.99
			Precision	[6,6]	0.96	0.90	0.66	0.54	0.99
				[7,7]	0.96	0.88	0.66	0.55	0.99
			Recall	[6,6]	0.92	0.77	0.55	0.45	0.97
				[7,7]	0.91	0.76	0.55	0.46	0.99
		RDP	F1-score	[6,6]	0.85	0.85	0.85	0.85	0.87
				[7,7]	0.86	0.85	0.85	0.85	0.88
			Precision	[6,6]	0.87	0.87	0.87	0.87	0.94
				[7,7]	0.87	0.87	0.87	0.87	0.95
			Recall	[6,6]	0.84	0.84	0.83	0.83	0.86
				[7,7]	0.85	0.84	0.84	0.83	0.87
		SILVA	F1-score	[6,6]	0.97	0.96	0.96	0.96	0.97
				[7,7]	0.98	0.97	0.96	0.96	0.98
			Precision	[6,6]	0.99	0.99	0.99	0.99	0.99
				[7,7]	0.99	0.99	0.99	0.99	0.99
			Recall	[6,6]	0.95	0.95	0.95	0.95	0.95
				[7,7]	0.98	0.96	0.95	0.95	0.97
	V4	GSR	F1-score	[6,6]	0.99	0.99	0.99	0.98	0.99
				[7,7]	0.99	0.99	0.99	0.98	0.99
			Precision	[6,6]	0.99	0.99	0.99	0.98	0.99
				[7,7]	0.99	0.99	0.99	0.98	0.99
			Recall	[6,6]	0.99	0.99	0.99	0.98	0.99
				[7,7]	0.99	0.99	0.99	0.98	0.99
		GTDB	F1-score	[6,6]	0.88	0.87	0.83	0.75	0.90
				[7,7]	0.88	0.87	0.83	0.73	0.90
			Precision	[6,6]	0.90	0.89	0.84	0.79	0.92
				[7,7]	0.90	0.89	0.84	0.79	0.92
			Recall	[6,6]	0.87	0.86	0.83	0.74	0.90
				[7,7]	0.87	0.86	0.83	0.72	0.90
		Greengenes	F1-score	[6,6]	0.96	0.94	0.89	0.86	0.98
				[7,7]	0.96	0.94	0.89	0.86	0.98
			Precision	[6,6]	0.96	0.93	0.88	0.86	0.98
				[7,7]	0.96	0.93	0.88	0.86	0.98

Continued on next page

Supplementary Table 2: N-gram-range and confidence threshold benchmarking at family level. F1-score, precision and recall are shown for each database, evaluated region and validation datasets. The values are the average of the five metagenomic samples. Bold represents the highest value.

Validation Dataset	Region	Database	Metrics	Conf. thres. N-gram	**0.5**	**0.7**	**0.9**	**0.98**	**disable**
			Recall	[6,6]	0.97	0.96	0.90	0.88	0.98
				[7,7]	0.97	0.96	0.90	0.88	0.98
		ITGDB	F1-score	[6,6]	0.99	0.83	0.55	0.39	0.99
				[7,7]	0.99	0.83	0.55	0.39	0.99
			Precision	[6,6]	0.99	0.85	0.60	0.39	0.99
				[7,7]	0.99	0.83	0.60	0.39	0.99
			Recall	[6,6]	0.99	0.82	0.53	0.39	0.99
				[7,7]	0.99	0.83	0.53	0.39	0.99
		RDP	F1-score	[6,6]	0.84	0.84	0.84	0.80	0.84
				[7,7]	0.84	0.84	0.84	0.84	0.84
			Precision	[6,6]	0.84	0.84	0.84	0.80	0.84
				[7,7]	0.84	0.84	0.84	0.84	0.84
			Recall	[6,6]	0.84	0.84	0.84	0.80	0.84
				[7,7]	0.84	0.84	0.84	0.84	0.84
		SILVA	F1-score	[6,6]	**1.00**	**1.00**	**1.00**	0.95	0.99
				[7,7]	**1.00**	**1.00**	**1.00**	0.95	0.99
			Precision	[6,6]	**1.00**	**1.00**	**1.00**	0.95	0.99
				[7,7]	**1.00**	**1.00**	**1.00**	0.95	0.99
			Recall	[6,6]	**1.00**	**1.00**	**1.00**	0.95	0.99
				[7,7]	**1.00**	**1.00**	**1.00**	0.95	0.99
	full-16S	GSR	F1-score	[6,6]	0.97	0.97	0.97	0.97	0.97
				[7,7]	0.97	0.97	0.97	0.97	0.98
			Precision	[6,6]	0.99	0.99	0.99	0.99	0.99
				[7,7]	0.99	0.99	0.99	0.99	0.99
			Recall	[6,6]	0.95	0.95	0.95	0.95	0.96
				[7,7]	0.96	0.96	0.95	0.95	0.98
		GTDB	F1-score	[6,6]	0.93	0.92	0.90	0.86	0.96
				[7,7]	0.93	0.93	0.92	0.90	0.96
			Precision	[6,6]	0.96	0.93	0.92	0.86	0.97
				[7,7]	0.93	0.93	0.93	0.92	0.96
			Recall	[6,6]	0.93	0.91	0.90	0.87	0.96
				[7,7]	0.94	0.93	0.91	0.90	0.97
		Greengenes	F1-score	[6,6]	0.96	0.96	0.95	0.91	0.96
				[7,7]	0.96	0.96	0.96	0.95	0.96
			Precision	[6,6]	0.98	0.98	0.97	0.92	0.98
				[7,7]	0.98	0.98	0.98	0.97	0.98
			Recall	[6,6]	0.94	0.94	0.94	0.91	0.94
				[7,7]	0.94	0.94	0.94	0.93	0.94
		ITGDB	F1-score	[6,6]	0.97	0.89	0.69	0.51	0.98
				[7,7]	0.97	0.91	0.74	0.59	**1.00**
			Precision	[6,6]	0.99	0.96	0.79	0.64	**1.00**
				[7,7]	0.99	0.97	0.90	0.69	**1.00**
			Recall	[6,6]	0.95	0.85	0.65	0.47	0.97
				[7,7]	0.96	0.87	0.68	0.55	0.99
		RDP	F1-score	[6,6]	0.85	0.85	0.85	0.85	0.85
				[7,7]	0.86	0.85	0.85	0.85	0.86
			Precision	[6,6]	0.88	0.87	0.88	0.88	0.88
				[7,7]	0.88	0.88	0.88	0.87	0.87
			Recall	[6,6]	0.84	0.84	0.84	0.84	0.84
				[7,7]	0.85	0.84	0.84	0.84	0.86
		SILVA	F1-score	[6,6]	0.97	0.97	0.96	0.96	0.97
				[7,7]	0.97	0.97	0.97	0.97	0.97
			Precision	[6,6]	0.99	0.99	0.99	0.98	0.99
				[7,7]	0.99	0.99	0.99	0.99	0.99
			Recall	[6,6]	0.95	0.95	0.95	0.95	0.95
				[7,7]	0.96	0.96	0.96	0.95	0.96
		GSR	F1-score	[6,6]	**1.00**	**1.00**	**1.00**	**1.00**	**1.00**
				[7,7]	**1.00**	**1.00**	**1.00**	**1.00**	**1.00**
			Precision	[6,6]	**1.00**	**1.00**	**1.00**	**1.00**	**1.00**
				[7,7]	**1.00**	**1.00**	**1.00**	**1.00**	**1.00**
			Recall	[6,6]	**1.00**	**1.00**	**1.00**	**1.00**	**1.00**

Continued on next page

Supplementary Table 2: N-gram-range and confidence threshold benchmarking at family level. F1-score, precision and recall are shown for each database, evaluated region and validation datasets. The values are the average of the five metagenomic samples. Bold represents the highest value.

Validation Dataset	Region	Database	Metrics	Conf. thres. / N-gram	**0.5**	**0.7**	**0.9**	**0.98**	**disable**
				[7,7]	**1.00**	**1.00**	**1.00**	**1.00**	**1.00**
		GTDB	F1-score	[6,6]	**1.00**	**1.00**	**1.00**	**1.00**	**1.00**
				[7,7]	**1.00**	**1.00**	**1.00**	**1.00**	**1.00**
			Precision	[6,6]	**1.00**	**1.00**	**1.00**	**1.00**	**1.00**
				[7,7]	**1.00**	**1.00**	**1.00**	**1.00**	**1.00**
			Recall	[6,6]	**1.00**	**1.00**	**1.00**	**1.00**	**1.00**
				[7,7]	**1.00**	**1.00**	**1.00**	**1.00**	**1.00**
		Greengenes	F1-score	[6,6]	**1.00**	**1.00**	**1.00**	**1.00**	**1.00**
				[7,7]	**1.00**	**1.00**	**1.00**	**1.00**	**1.00**
			Precision	[6,6]	**1.00**	**1.00**	**1.00**	**1.00**	**1.00**
				[7,7]	**1.00**	**1.00**	**1.00**	**1.00**	**1.00**
			Recall	[6,6]	**1.00**	**1.00**	**1.00**	**1.00**	**1.00**
				[7,7]	**1.00**	**1.00**	**1.00**	**1.00**	**1.00**
		ITGDB	F1-score	[6,6]	**1.00**	0.99	0.98	0.98	**1.00**
				[7,7]	**1.00**	**1.00**	0.98	0.98	**1.00**
			Precision	[6,6]	**1.00**	**1.00**	**1.00**	**1.00**	**1.00**
				[7,7]	**1.00**	**1.00**	**1.00**	**1.00**	**1.00**
			Recall	[6,6]	**1.00**	0.98	0.98	0.98	**1.00**
				[7,7]	**1.00**	**1.00**	0.98	0.98	**1.00**
		RDP	F1-score	[6,6]	0.99	0.99	0.99	0.99	0.99
				[7,7]	0.99	0.99	0.99	0.99	0.99
			Precision	[6,6]	0.99	0.99	0.99	0.99	0.99
				[7,7]	0.99	0.99	0.99	0.99	0.99
			Recall	[6,6]	0.99	0.99	0.99	0.99	0.99
				[7,7]	0.99	0.99	0.99	0.99	0.99
		SILVA	F1-score	[6,6]	**1.00**	**1.00**	**1.00**	**1.00**	**1.00**
				[7,7]	**1.00**	**1.00**	**1.00**	**1.00**	**1.00**
			Precision	[6,6]	**1.00**	**1.00**	**1.00**	**1.00**	**1.00**
				[7,7]	**1.00**	**1.00**	**1.00**	**1.00**	**1.00**
			Recall	[6,6]	**1.00**	**1.00**	**1.00**	**1.00**	**1.00**
				[7,7]	**1.00**	**1.00**	**1.00**	**1.00**	**1.00**
	V3-V4	GSR	F1-score	[6,6]	**1.00**	**1.00**	**1.00**	**1.00**	**1.00**
				[7,7]	**1.00**	**1.00**	**1.00**	**1.00**	**1.00**
			Precision	[6,6]	**1.00**	**1.00**	**1.00**	**1.00**	**1.00**
				[7,7]	**1.00**	**1.00**	**1.00**	**1.00**	**1.00**
			Recall	[6,6]	**1.00**	**1.00**	**1.00**	**1.00**	**1.00**
				[7,7]	**1.00**	**1.00**	**1.00**	**1.00**	**1.00**
		GTDB	F1-score	[6,6]	**1.00**	**1.00**	0.97	0.93	**1.00**
				[7,7]	**1.00**	**1.00**	**1.00**	**1.00**	**1.00**
			Precision	[6,6]	**1.00**	**1.00**	**1.00**	**1.00**	**1.00**
				[7,7]	**1.00**	**1.00**	**1.00**	**1.00**	**1.00**
			Recall	[6,6]	**1.00**	**1.00**	0.94	0.88	**1.00**
				[7,7]	**1.00**	**1.00**	**1.00**	**1.00**	**1.00**
		Greengenes	F1-score	[6,6]	**1.00**	**1.00**	**1.00**	**1.00**	**1.00**
				[7,7]	**1.00**	**1.00**	**1.00**	**1.00**	**1.00**
			Precision	[6,6]	**1.00**	**1.00**	**1.00**	**1.00**	**1.00**
				[7,7]	**1.00**	**1.00**	**1.00**	**1.00**	**1.00**
			Recall	[6,6]	**1.00**	**1.00**	**1.00**	**1.00**	**1.00**
				[7,7]	**1.00**	**1.00**	**1.00**	**1.00**	**1.00**
		ITGDB	F1-score	[6,6]	0.99	0.99	0.99	0.98	**1.00**
				[7,7]	0.99	0.99	0.99	0.99	**1.00**
			Precision	[6,6]	**1.00**	**1.00**	0.99	0.99	**1.00**
				[7,7]	**1.00**	**1.00**	0.99	0.99	**1.00**
			Recall	[6,6]	0.99	0.99	0.99	0.97	**1.00**
				[7,7]	0.99	0.99	0.99	0.99	**1.00**
		RDP	F1-score	[6,6]	**1.00**	**1.00**	**1.00**	**1.00**	**1.00**
				[7,7]	**1.00**	**1.00**	**1.00**	**1.00**	**1.00**
			Precision	[6,6]	**1.00**	**1.00**	**1.00**	**1.00**	**1.00**
				[7,7]	**1.00**	**1.00**	**1.00**	**1.00**	**1.00**
			Recall	[6,6]	**1.00**	**1.00**	**1.00**	**1.00**	**1.00**
				[7,7]	**1.00**	**1.00**	**1.00**	**1.00**	**1.00**

Continued on next page

Supplementary Table 2: N-gram-range and confidence threshold benchmarking at family level. F1-score, precision and recall are shown for each database, evaluated region and validation datasets. The values are the average of the five metagenomic samples. Bold represents the highest value.

Validation Dataset	Region	Database	Metrics	Conf. thres. / N-gram	**0.5**	**0.7**	**0.9**	**0.98**	**disable**
		SILVA	F1-score	[6,6]	**1.00**	0.99	0.99	0.99	**1.00**
				[7,7]	**1.00**	**1.00**	0.99	0.99	**1.00**
			Precision	[6,6]	**1.00**	0.99	0.99	0.99	**1.00**
				[7,7]	**1.00**	**1.00**	0.99	0.99	**1.00**
			Recall	[6,6]	**1.00**	0.99	0.99	0.99	**1.00**
				[7,7]	**1.00**	**1.00**	0.99	0.99	**1.00**
	V3-V5	GSR	F1-score	[6,6]	**1.00**	**1.00**	**1.00**	**1.00**	**1.00**
				[7,7]	**1.00**	**1.00**	**1.00**	**1.00**	**1.00**
			Precision	[6,6]	**1.00**	**1.00**	**1.00**	**1.00**	**1.00**
				[7,7]	**1.00**	**1.00**	**1.00**	**1.00**	**1.00**
			Recall	[6,6]	**1.00**	**1.00**	**1.00**	**1.00**	**1.00**
				[7,7]	**1.00**	**1.00**	**1.00**	**1.00**	**1.00**
		GTDB	F1-score	[6,6]	**1.00**	**1.00**	**1.00**	0.97	**1.00**
				[7,7]	**1.00**	**1.00**	**1.00**	**1.00**	**1.00**
			Precision	[6,6]	**1.00**	**1.00**	**1.00**	**1.00**	**1.00**
				[7,7]	**1.00**	**1.00**	**1.00**	**1.00**	**1.00**
			Recall	[6,6]	**1.00**	**1.00**	**1.00**	0.95	**1.00**
				[7,7]	**1.00**	**1.00**	**1.00**	**1.00**	**1.00**
		Greengenes	F1-score	[6,6]	**1.00**	**1.00**	**1.00**	**1.00**	**1.00**
				[7,7]	**1.00**	**1.00**	**1.00**	**1.00**	**1.00**
			Precision	[6,6]	**1.00**	**1.00**	**1.00**	**1.00**	**1.00**
				[7,7]	**1.00**	**1.00**	**1.00**	**1.00**	**1.00**
			Recall	[6,6]	**1.00**	**1.00**	**1.00**	**1.00**	**1.00**
				[7,7]	**1.00**	**1.00**	**1.00**	**1.00**	**1.00**
		ITGDB	F1-score	[6,6]	0.99	0.99	0.97	0.97	**1.00**
				[7,7]	0.99	0.99	0.97	0.97	**1.00**
			Precision	[6,6]	**1.00**	**1.00**	**1.00**	**1.00**	**1.00**
				[7,7]	**1.00**	**1.00**	**1.00**	**1.00**	**1.00**
			Recall	[6,6]	0.99	0.98	0.96	0.96	**1.00**
				[7,7]	0.99	0.99	0.96	0.96	**1.00**
		RDP	F1-score	[6,6]	**1.00**	**1.00**	**1.00**	**1.00**	**1.00**
				[7,7]	**1.00**	**1.00**	**1.00**	**1.00**	**1.00**
			Precision	[6,6]	**1.00**	**1.00**	**1.00**	**1.00**	**1.00**
				[7,7]	**1.00**	**1.00**	**1.00**	**1.00**	**1.00**
			Recall	[6,6]	**1.00**	**1.00**	**1.00**	**1.00**	**1.00**
				[7,7]	**1.00**	**1.00**	**1.00**	**1.00**	**1.00**
		SILVA	F1-score	[6,6]	**1.00**	**1.00**	0.99	0.99	**1.00**
				[7,7]	**1.00**	**1.00**	**1.00**	0.99	**1.00**
			Precision	[6,6]	**1.00**	**1.00**	0.99	0.99	**1.00**
				[7,7]	**1.00**	**1.00**	**1.00**	0.99	**1.00**
			Recall	[6,6]	**1.00**	0.99	0.99	0.99	**1.00**
				[7,7]	**1.00**	**1.00**	0.99	0.99	**1.00**
	V4	GSR	F1-score	[6,6]	**1.00**	**1.00**	**1.00**	0.99	**1.00**
				[7,7]	**1.00**	**1.00**	**1.00**	0.99	**1.00**
			Precision	[6,6]	**1.00**	**1.00**	**1.00**	**1.00**	**1.00**
				[7,7]	**1.00**	**1.00**	**1.00**	**1.00**	**1.00**
			Recall	[6,6]	**1.00**	**1.00**	**1.00**	0.99	**1.00**
				[7,7]	**1.00**	**1.00**	**1.00**	0.99	**1.00**
		GTDB	F1-score	[6,6]	0.94	0.94	0.91	0.91	0.94
				[7,7]	**1.00**	**1.00**	0.94	0.91	**1.00**
			Precision	[6,6]	**1.00**	**1.00**	**1.00**	**1.00**	**1.00**
				[7,7]	**1.00**	**1.00**	**1.00**	**1.00**	**1.00**
			Recall	[6,6]	0.90	0.90	0.85	0.85	0.90
				[7,7]	**1.00**	**1.00**	0.90	0.85	**1.00**
		Greengenes	F1-score	[6,6]	**1.00**	**1.00**	0.99	0.95	**1.00**
				[7,7]	**1.00**	**1.00**	**1.00**	0.96	**1.00**
			Precision	[6,6]	**1.00**	**1.00**	**1.00**	**1.00**	**1.00**
				[7,7]	**1.00**	**1.00**	**1.00**	**1.00**	**1.00**
			Recall	[6,6]	**1.00**	**1.00**	0.97	0.91	**1.00**
				[7,7]	**1.00**	**1.00**	**1.00**	0.92	**1.00**
		ITGDB	F1-score	[6,6]	**1.00**	0.96	0.96	0.95	**1.00**

Continued on next page

Supplementary Table 2: N-gram-range and confidence threshold benchmarking at family level. F1-score, precision and recall are shown for each database, evaluated region and validation datasets. The values are the average of the five metagenomic samples. Bold represents the highest value.

Validation Dataset	Region	Database	Metrics	Conf. thres. / N-gram	**0.5**	**0.7**	**0.9**	**0.98**	**disable**
				[7,7]	**1.00**	0.96	0.96	0.96	**1.00**
			Precision	[6,6]	**1.00**	0.99	0.99	0.99	**1.00**
				[7,7]	**1.00**	0.99	0.99	0.99	**1.00**
			Recall	[6,6]	0.99	0.95	0.94	0.94	**1.00**
				[7,7]	0.99	0.95	0.94	0.94	**1.00**
		RDP	F1-score	[6,6]	**1.00**	**1.00**	0.97	0.95	**1.00**
				[7,7]	**1.00**	**1.00**	**1.00**	0.95	**1.00**
			Precision	[6,6]	**1.00**	**1.00**	**1.00**	**1.00**	**1.00**
				[7,7]	**1.00**	**1.00**	**1.00**	**1.00**	**1.00**
			Recall	[6,6]	**1.00**	**1.00**	0.95	0.90	**1.00**
				[7,7]	**1.00**	**1.00**	**1.00**	0.90	**1.00**
		SILVA	F1-score	[6,6]	0.99	0.99	0.99	0.96	**1.00**
				[7,7]	0.99	0.99	0.99	0.94	**1.00**
			Precision	[6,6]	0.99	0.99	0.99	0.99	**1.00**
				[7,7]	0.99	0.99	0.99	0.99	**1.00**
			Recall	[6,6]	0.99	0.99	0.99	0.94	**1.00**
				[7,7]	0.99	0.99	0.99	0.89	**1.00**
	full-16S	GSR	F1-score	[6,6]	**1.00**	**1.00**	**1.00**	**1.00**	**1.00**
				[7,7]	**1.00**	**1.00**	**1.00**	**1.00**	**1.00**
			Precision	[6,6]	**1.00**	**1.00**	**1.00**	**1.00**	**1.00**
				[7,7]	**1.00**	**1.00**	**1.00**	**1.00**	**1.00**
			Recall	[6,6]	**1.00**	**1.00**	**1.00**	**1.00**	**1.00**
				[7,7]	**1.00**	**1.00**	**1.00**	**1.00**	**1.00**
		GTDB	F1-score	[6,6]	0.97	0.97	0.95	0.95	0.97
				[7,7]	0.99	0.99	0.99	0.99	0.99
			Precision	[6,6]	0.99	0.99	0.99	0.99	0.99
				[7,7]	0.99	0.99	0.99	0.99	0.99
			Recall	[6,6]	0.95	0.95	0.92	0.92	0.95
				[7,7]	0.99	0.99	0.99	0.99	0.99
		Greengenes	F1-score	[6,6]	0.99	0.99	0.99	0.98	0.99
				[7,7]	**1.00**	**1.00**	**1.00**	**1.00**	**1.00**
			Precision	[6,6]	**1.00**	**1.00**	**1.00**	**1.00**	**1.00**
				[7,7]	**1.00**	**1.00**	**1.00**	**1.00**	**1.00**
			Recall	[6,6]	0.98	0.98	0.98	0.96	0.98
				[7,7]	**1.00**	**1.00**	**1.00**	**1.00**	**1.00**
		ITGDB	F1-score	[6,6]	**1.00**	0.99	0.98	0.97	**1.00**
				[7,7]	**1.00**	0.99	0.98	0.98	**1.00**
			Precision	[6,6]	**1.00**	**1.00**	**1.00**	**1.00**	**1.00**
				[7,7]	**1.00**	**1.00**	**1.00**	**1.00**	**1.00**
			Recall	[6,6]	**1.00**	0.99	0.97	0.96	**1.00**
				[7,7]	**1.00**	0.99	0.97	0.97	**1.00**
		RDP	F1-score	[6,6]	**1.00**	**1.00**	**1.00**	**1.00**	**1.00**
				[7,7]	**1.00**	**1.00**	**1.00**	**1.00**	**1.00**
			Precision	[6,6]	**1.00**	**1.00**	**1.00**	**1.00**	**1.00**
				[7,7]	**1.00**	**1.00**	**1.00**	**1.00**	**1.00**
			Recall	[6,6]	**1.00**	**1.00**	**1.00**	**1.00**	**1.00**
				[7,7]	**1.00**	**1.00**	**1.00**	**1.00**	**1.00**
		SILVA	F1-score	[6,6]	**1.00**	**1.00**	**1.00**	**1.00**	**1.00**
				[7,7]	**1.00**	**1.00**	**1.00**	**1.00**	**1.00**
			Precision	[6,6]	**1.00**	**1.00**	**1.00**	**1.00**	**1.00**
				[7,7]	**1.00**	**1.00**	**1.00**	**1.00**	**1.00**
			Recall	[6,6]	**1.00**	**1.00**	**1.00**	**1.00**	**1.00**
				[7,7]	**1.00**	**1.00**	**1.00**	**1.00**	**1.00**

Supplementary Table 3: N-gram-range and confidence threshold benchmarking at genus level. F1-score, precision and recall are shown for each database, evaluated region and validation datasets. The values are the average of the five metagenomic samples. Bold represents the highest value.

Validation Dataset	Region	Database	Metrics	Conf. thres. / N-gram	**0.5**	**0.7**	**0.9**	**0.98**	**disable**
	V1-V3	GSR	F1-score	[6,6]	0.98	0.98	0.97	0.96	0.98
				[7,7]	0.98	0.98	0.97	0.97	0.98
			Precision	[6,6]	0.99	0.99	0.98	0.98	0.99
				[7,7]	0.99	0.99	0.98	0.98	0.99
			Recall	[6,6]	0.98	0.98	0.97	0.96	0.98
				[7,7]	0.98	0.98	0.97	0.96	0.98
		GTDB	F1-score	[6,6]	0.85	0.82	0.81	0.79	0.85
				[7,7]	0.84	0.83	0.81	0.78	0.84
			Precision	[6,6]	0.87	0.86	0.85	0.85	0.87
				[7,7]	0.87	0.86	0.85	0.85	0.87
			Recall	[6,6]	0.85	0.80	0.79	0.76	0.85
				[7,7]	0.83	0.81	0.79	0.76	0.83
		Greengenes	F1-score	[6,6]	0.67	0.68	0.68	0.65	0.67
				[7,7]	0.67	0.68	0.68	0.68	0.67
			Precision	[6,6]	0.76	0.77	0.77	0.78	0.76
				[7,7]	0.77	0.78	0.78	0.77	0.77
			Recall	[6,6]	0.68	0.70	0.69	0.66	0.69
				[7,7]	0.69	0.70	0.70	0.69	0.69
		ITGDB	F1-score	[6,6]	0.85	0.80	0.73	0.67	0.89
				[7,7]	0.85	0.78	0.74	0.68	0.89
			Precision	[6,6]	0.86	0.86	0.84	0.80	0.90
				[7,7]	0.86	0.85	0.84	0.86	0.90
			Recall	[6,6]	0.86	0.77	0.69	0.62	0.90
				[7,7]	0.85	0.74	0.69	0.63	0.90
		RDP	F1-score	[6,6]	0.72	0.70	0.70	0.69	0.73
				[7,7]	0.71	0.71	0.70	0.70	0.71
			Precision	[6,6]	0.75	0.74	0.74	0.74	0.76
				[7,7]	0.75	0.76	0.74	0.74	0.75
			Recall	[6,6]	0.72	0.70	0.70	0.70	0.73
				[7,7]	0.71	0.71	0.70	0.70	0.71
		SILVA	F1-score	[6,6]	0.90	0.89	0.82	0.77	0.91
				[7,7]	0.92	0.92	0.87	0.85	0.93
			Precision	[6,6]	0.93	0.92	0.89	0.87	0.94
				[7,7]	0.93	0.93	0.89	0.89	0.94
			Recall	[6,6]	0.90	0.88	0.80	0.75	0.91
				[7,7]	0.92	0.92	0.87	0.84	0.92
	V3-V4	GSR	F1-score	[6,6]	0.96	0.98	0.97	0.94	0.96
				[7,7]	0.99	0.99	0.99	0.95	0.99
			Precision	[6,6]	0.96	0.99	0.99	0.95	0.97
				[7,7]	**1.00**	0.99	0.99	0.97	**1.00**
			Recall	[6,6]	0.97	0.98	0.96	0.93	0.97
				[7,7]	0.99	0.99	0.98	0.94	0.99
		GTDB	F1-score	[6,6]	0.83	0.80	0.75	0.62	0.86
				[7,7]	0.82	0.81	0.75	0.62	0.86
			Precision	[6,6]	0.87	0.87	0.83	0.73	0.87
				[7,7]	0.87	0.87	0.84	0.73	0.87
			Recall	[6,6]	0.82	0.78	0.72	0.57	0.86
				[7,7]	0.81	0.79	0.72	0.57	0.86
		Greengenes	F1-score	[6,6]	0.69	0.67	0.66	0.63	0.69
				[7,7]	0.70	0.70	0.69	0.65	0.70
			Precision	[6,6]	0.76	0.75	0.73	0.71	0.76
				[7,7]	0.77	0.76	0.75	0.72	0.77
			Recall	[6,6]	0.71	0.69	0.68	0.63	0.71
				[7,7]	0.71	0.71	0.70	0.66	0.72
		ITGDB	F1-score	[6,6]	0.78	0.72	0.58	0.49	0.90
				[7,7]	0.82	0.70	0.59	0.52	0.92
			Precision	[6,6]	0.80	0.82	0.79	0.70	0.91
				[7,7]	0.84	0.83	0.79	0.72	0.93
			Recall	[6,6]	0.77	0.68	0.53	0.46	0.90
				[7,7]	0.80	0.66	0.54	0.47	0.92

Continued on next page

Supplementary Table 3: N-gram-range and confidence threshold benchmarking at genus level. F1-score, precision and recall are shown for each database, evaluated region and validation datasets. The values are the average of the five metagenomic samples. Bold represents the highest value.

Validation Dataset	Region	Database	Metrics	Conf. thres. / N-gram	**0.5**	**0.7**	**0.9**	**0.98**	**disable**
		RDP	F1-score	[6,6]	0.91	0.84	0.75	0.73	0.91
				[7,7]	0.89	0.84	0.81	0.74	0.91
			Precision	[6,6]	0.92	0.92	0.92	0.89	0.92
				[7,7]	0.92	0.92	0.92	0.91	0.92
			Recall	[6,6]	0.90	0.81	0.73	0.70	0.90
				[7,7]	0.88	0.81	0.79	0.72	0.90
		SILVA	F1-score	[6,6]	0.89	0.86	0.83	0.70	0.89
				[7,7]	0.88	0.87	0.87	0.86	0.89
			Precision	[6,6]	0.91	0.89	0.87	0.82	0.91
				[7,7]	0.91	0.90	0.90	0.90	0.92
			Recall	[6,6]	0.87	0.85	0.81	0.66	0.88
				[7,7]	0.87	0.86	0.85	0.84	0.88
	V3-V5	GSR	F1-score	[6,6]	0.98	0.97	0.95	0.92	0.98
				[7,7]	0.98	0.98	0.97	0.94	0.98
			Precision	[6,6]	0.99	0.97	0.95	0.94	0.99
				[7,7]	0.99	0.99	0.98	0.95	0.99
			Recall	[6,6]	0.98	0.97	0.95	0.92	0.98
				[7,7]	0.98	0.98	0.97	0.94	0.98
		GTDB	F1-score	[6,6]	0.85	0.77	0.70	0.63	0.87
				[7,7]	0.84	0.78	0.71	0.64	0.85
			Precision	[6,6]	0.88	0.87	0.84	0.80	0.88
				[7,7]	0.87	0.87	0.86	0.84	0.87
			Recall	[6,6]	0.84	0.75	0.68	0.61	0.87
				[7,7]	0.82	0.75	0.69	0.60	0.85
		Greengenes	F1-score	[6,6]	0.68	0.68	0.67	0.66	0.68
				[7,7]	0.70	0.69	0.69	0.69	0.70
			Precision	[6,6]	0.77	0.76	0.76	0.76	0.77
				[7,7]	0.78	0.77	0.77	0.78	0.77
			Recall	[6,6]	0.71	0.70	0.69	0.67	0.71
				[7,7]	0.72	0.72	0.71	0.71	0.72
		ITGDB	F1-score	[6,6]	0.82	0.75	0.67	0.53	0.91
				[7,7]	0.82	0.74	0.67	0.55	0.91
			Precision	[6,6]	0.84	0.83	0.82	0.80	0.92
				[7,7]	0.84	0.85	0.84	0.83	0.92
			Recall	[6,6]	0.83	0.72	0.61	0.47	0.91
				[7,7]	0.82	0.69	0.61	0.48	0.91
		RDP	F1-score	[6,6]	0.87	0.86	0.75	0.72	0.89
				[7,7]	0.88	0.86	0.77	0.75	0.90
			Precision	[6,6]	0.91	0.92	0.90	0.88	0.92
				[7,7]	0.91	0.91	0.91	0.90	0.92
			Recall	[6,6]	0.85	0.83	0.73	0.71	0.89
				[7,7]	0.86	0.84	0.76	0.74	0.90
		SILVA	F1-score	[6,6]	0.89	0.86	0.82	0.72	0.91
				[7,7]	0.91	0.90	0.86	0.83	0.91
			Precision	[6,6]	0.92	0.87	0.86	0.84	0.93
				[7,7]	0.93	0.93	0.89	0.87	0.93
			Recall	[6,6]	0.89	0.86	0.80	0.69	0.91
				[7,7]	0.91	0.90	0.85	0.82	0.91
		GSR	F1-score	[6,6]	0.95	0.94	0.92	0.86	0.96
				[7,7]	0.96	0.97	0.94	0.88	0.96
			Precision	[6,6]	0.95	0.94	0.94	0.93	0.96
				[7,7]	0.96	0.97	0.96	0.94	0.96
			Recall	[6,6]	0.96	0.94	0.91	0.83	0.97
				[7,7]	0.97	0.96	0.93	0.86	0.97
		GTDB	F1-score	[6,6]	0.78	0.72	0.63	0.45	0.84
				[7,7]	0.75	0.72	0.64	0.48	0.82
			Precision	[6,6]	0.85	0.81	0.78	0.67	0.87
				[7,7]	0.82	0.80	0.81	0.72	0.84
			Recall	[6,6]	0.76	0.69	0.60	0.39	0.83
				[7,7]	0.73	0.69	0.61	0.42	0.81
		Greengenes	F1-score	[6,6]	0.71	0.70	0.65	0.63	0.72

Continued on next page

Supplementary Table 3: N-gram-range and confidence threshold benchmarking at genus level. F1-score, precision and recall are shown for each database, evaluated region and validation datasets. The values are the average of the five metagenomic samples. Bold represents the highest value.

Validation Dataset	Region	Database	Metrics	Conf. thres. / N-gram	**0.5**	**0.7**	**0.9**	**0.98**	**disable**
				[7,7]	0.71	0.71	0.67	0.66	0.72
			Precision	[6,6]	0.79	0.79	0.76	0.76	0.80
				[7,7]	0.80	0.79	0.77	0.77	0.80
			Recall	[6,6]	0.72	0.71	0.66	0.63	0.73
				[7,7]	0.72	0.72	0.68	0.66	0.73
		ITGDB	F1-score	[6,6]	0.78	0.64	0.53	0.35	0.90
				[7,7]	0.78	0.67	0.53	0.43	0.89
			Precision	[6,6]	0.80	0.78	0.76	0.72	0.91
				[7,7]	0.81	0.81	0.76	0.75	0.91
			Recall	[6,6]	0.77	0.60	0.48	0.30	0.90
				[7,7]	0.76	0.63	0.48	0.35	0.90
		RDP	F1-score	[6,6]	0.89	0.89	0.71	0.69	0.90
				[7,7]	0.89	0.89	0.74	0.71	0.91
			Precision	[6,6]	0.90	0.90	0.89	0.88	0.91
				[7,7]	0.91	0.90	0.89	0.89	0.92
			Recall	[6,6]	0.88	0.88	0.69	0.67	0.90
				[7,7]	0.89	0.88	0.72	0.69	0.90
		SILVA	F1-score	[6,6]	0.87	0.85	0.71	0.58	0.88
				[7,7]	0.87	0.87	0.83	0.59	0.88
			Precision	[6,6]	0.90	0.89	0.87	0.81	0.90
				[7,7]	0.90	0.90	0.89	0.86	0.91
			Recall	[6,6]	0.86	0.84	0.68	0.56	0.87
				[7,7]	0.86	0.85	0.80	0.57	0.86
	full-16S	GSR	F1-score	[6,6]	**1.00**	0.99	0.98	0.95	**1.00**
				[7,7]	**1.00**	**1.00**	0.99	0.98	**1.00**
			Precision	[6,6]	**1.00**	0.99	0.99	0.96	**1.00**
				[7,7]	**1.00**	**1.00**	0.99	0.99	**1.00**
			Recall	[6,6]	**1.00**	0.99	0.98	0.95	**1.00**
				[7,7]	**1.00**	**1.00**	0.99	0.98	**1.00**
		GTDB	F1-score	[6,6]	0.75	0.61	0.54	0.49	0.80
				[7,7]	0.75	0.72	0.64	0.61	0.75
			Precision	[6,6]	0.83	0.81	0.79	0.75	0.84
				[7,7]	0.79	0.79	0.75	0.76	0.79
			Recall	[6,6]	0.72	0.60	0.54	0.48	0.78
				[7,7]	0.75	0.70	0.62	0.59	0.75
		Greengenes	F1-score	[6,6]	0.71	0.70	0.69	0.66	0.71
				[7,7]	0.72	0.72	0.71	0.70	0.72
			Precision	[6,6]	0.79	0.79	0.78	0.79	0.80
				[7,7]	0.80	0.80	0.79	0.79	0.80
			Recall	[6,6]	0.73	0.72	0.70	0.66	0.73
				[7,7]	0.74	0.73	0.73	0.72	0.74
		ITGDB	F1-score	[6,6]	0.87	0.83	0.75	0.61	0.92
				[7,7]	0.87	0.84	0.75	0.66	0.93
			Precision	[6,6]	0.87	0.89	0.88	0.80	0.93
				[7,7]	0.88	0.89	0.89	0.84	0.94
			Recall	[6,6]	0.88	0.80	0.70	0.55	0.92
				[7,7]	0.88	0.81	0.69	0.59	0.93
		RDP	F1-score	[6,6]	0.91	0.91	0.89	0.88	0.91
				[7,7]	0.91	0.91	0.91	0.89	0.91
			Precision	[6,6]	0.92	0.92	0.92	0.91	0.93
				[7,7]	0.92	0.93	0.92	0.92	0.92
			Recall	[6,6]	0.90	0.90	0.88	0.86	0.90
				[7,7]	0.91	0.90	0.90	0.89	0.91
		SILVA	F1-score	[6,6]	0.92	0.92	0.89	0.82	0.93
				[7,7]	0.94	0.94	0.93	0.92	0.94
			Precision	[6,6]	0.94	0.93	0.91	0.87	0.95
				[7,7]	0.96	0.95	0.94	0.94	0.95
			Recall	[6,6]	0.92	0.92	0.88	0.79	0.93
				[7,7]	0.94	0.94	0.93	0.92	0.94
		GSR	F1-score	[6,6]	0.89	0.88	0.86	0.86	0.91
				[7,7]	0.89	0.88	0.88	0.87	0.90

Continued on next page

Supplementary Table 3: N-gram-range and confidence threshold benchmarking at genus level. F1-score, precision and recall are shown for each database, evaluated region and validation datasets. The values are the average of the five metagenomic samples. Bold represents the highest value.

Validation Dataset	Region	Database	Metrics	Conf. thres. N-gram	**0.5**	**0.7**	**0.9**	**0.98**	**disable**
			Precision	[6,6]	0.91	0.88	0.87	0.87	0.94
				[7,7]	0.91	0.88	0.88	0.87	0.93
			Recall	[6,6]	0.89	0.88	0.86	0.86	0.90
				[7,7]	0.89	0.88	0.87	0.86	0.90
		GTDB	F1-score	[6,6]	0.85	0.83	0.82	0.81	0.85
				[7,7]	0.84	0.84	0.82	0.82	0.85
			Precision	[6,6]	0.90	0.89	0.86	0.85	0.90
				[7,7]	0.90	0.90	0.87	0.86	0.89
			Recall	[6,6]	0.83	0.82	0.81	0.79	0.84
				[7,7]	0.82	0.82	0.81	0.80	0.84
		Greengenes	F1-score	[6,6]	0.81	0.81	0.77	0.70	0.81
				[7,7]	0.82	0.81	0.79	0.76	0.82
			Precision	[6,6]	0.84	0.84	0.79	0.73	0.84
				[7,7]	0.85	0.85	0.83	0.78	0.85
			Recall	[6,6]	0.81	0.81	0.77	0.70	0.81
				[7,7]	0.82	0.81	0.79	0.76	0.82
		ITGDB	F1-score	[6,6]	0.78	0.73	0.62	0.42	0.83
				[7,7]	0.79	0.73	0.63	0.44	0.83
			Precision	[6,6]	0.83	0.81	0.77	0.52	0.86
				[7,7]	0.83	0.81	0.73	0.54	0.86
			Recall	[6,6]	0.77	0.69	0.58	0.39	0.83
				[7,7]	0.78	0.70	0.59	0.41	0.83
		RDP	F1-score	[6,6]	0.79	0.79	0.77	0.77	0.80
				[7,7]	0.79	0.79	0.79	0.77	0.80
			Precision	[6,6]	0.83	0.83	0.82	0.81	0.83
				[7,7]	0.83	0.83	0.83	0.81	0.83
			Recall	[6,6]	0.78	0.78	0.76	0.76	0.79
				[7,7]	0.78	0.78	0.78	0.76	0.79
		SILVA	F1-score	[6,6]	0.92	0.92	0.83	0.78	0.91
				[7,7]	0.92	0.92	0.89	0.83	0.92
			Precision	[6,6]	0.96	0.96	0.88	0.84	0.95
				[7,7]	0.96	0.96	0.95	0.88	0.96
			Recall	[6,6]	0.91	0.91	0.83	0.76	0.90
				[7,7]	0.92	0.92	0.87	0.81	0.92
	V3-V4	GSR	F1-score	[6,6]	0.93	0.93	0.91	0.91	0.99
				[7,7]	0.96	0.93	0.93	0.91	0.99
			Precision	[6,6]	0.93	0.93	0.91	0.91	0.99
				[7,7]	0.96	0.93	0.93	0.91	0.99
			Recall	[6,6]	0.93	0.93	0.91	0.91	0.99
				[7,7]	0.96	0.93	0.93	0.91	0.99
		GTDB	F1-score	[6,6]	0.89	0.89	0.84	0.79	0.98
				[7,7]	0.89	0.89	0.82	0.79	0.98
			Precision	[6,6]	0.89	0.89	0.84	0.79	0.98
				[7,7]	0.89	0.89	0.82	0.79	0.98
			Recall	[6,6]	0.89	0.89	0.84	0.79	0.98
				[7,7]	0.89	0.89	0.82	0.79	0.98
		Greengenes	F1-score	[6,6]	0.92	0.88	0.77	0.64	0.92
				[7,7]	0.92	0.88	0.83	0.75	0.92
			Precision	[6,6]	0.92	0.87	0.77	0.64	0.92
				[7,7]	0.92	0.87	0.82	0.74	0.92
			Recall	[6,6]	0.93	0.89	0.78	0.66	0.93
				[7,7]	0.93	0.89	0.84	0.76	0.93
		ITGDB	F1-score	[6,6]	0.96	0.82	0.60	0.54	0.99
				[7,7]	0.96	0.82	0.60	0.49	0.99
			Precision	[6,6]	0.96	0.82	0.60	0.54	0.99
				[7,7]	0.96	0.82	0.60	0.49	0.99
			Recall	[6,6]	0.96	0.82	0.60	0.54	0.99
				[7,7]	0.96	0.82	0.60	0.49	0.99
		RDP	F1-score	[6,6]	0.80	0.71	0.64	0.64	0.80
				[7,7]	0.71	0.71	0.65	0.64	0.80
			Precision	[6,6]	0.80	0.71	0.64	0.64	0.80

Continued on next page

Supplementary Table 3: N-gram-range and confidence threshold benchmarking at genus level. F1-score, precision and recall are shown for each database, evaluated region and validation datasets. The values are the average of the five metagenomic samples. Bold represents the highest value.

Validation Dataset	Region	Database	Metrics	Conf. thres. N-gram	**0.5**	**0.7**	**0.9**	**0.98**	**disable**
				[7,7]	0.71	0.71	0.65	0.64	0.80
			Recall	[6,6]	0.80	0.71	0.64	0.64	0.80
				[7,7]	0.71	0.71	0.65	0.64	0.80
		SILVA	F1-score	[6,6]	0.99	0.97	0.96	0.85	0.99
				[7,7]	0.99	0.99	0.97	0.96	0.99
			Precision	[6,6]	0.99	0.97	0.96	0.84	0.99
				[7,7]	0.99	0.99	0.97	0.96	0.99
			Recall	[6,6]	0.99	0.97	0.96	0.88	0.99
				[7,7]	0.99	0.99	0.97	0.96	0.99
	V3-V5	GSR	F1-score	[6,6]	0.84	0.82	0.80	0.78	0.86
				[7,7]	0.84	0.83	0.81	0.79	0.86
			Precision	[6,6]	0.83	0.82	0.80	0.78	0.88
				[7,7]	0.85	0.82	0.81	0.78	0.88
			Recall	[6,6]	0.86	0.84	0.82	0.80	0.88
				[7,7]	0.86	0.84	0.82	0.80	0.88
		GTDB	F1-score	[6,6]	0.79	0.79	0.76	0.71	0.83
				[7,7]	0.79	0.79	0.75	0.71	0.83
			Precision	[6,6]	0.84	0.84	0.81	0.76	0.85
				[7,7]	0.84	0.84	0.80	0.77	0.85
			Recall	[6,6]	0.80	0.80	0.77	0.71	0.84
				[7,7]	0.80	0.80	0.75	0.72	0.84
		Greengenes	F1-score	[6,6]	0.77	0.75	0.69	0.60	0.77
				[7,7]	0.77	0.75	0.72	0.64	0.77
			Precision	[6,6]	0.78	0.75	0.70	0.61	0.78
				[7,7]	0.79	0.75	0.73	0.67	0.78
			Recall	[6,6]	0.79	0.77	0.71	0.62	0.79
				[7,7]	0.80	0.77	0.74	0.66	0.80
		ITGDB	F1-score	[6,6]	0.83	0.70	0.45	0.36	0.89
				[7,7]	0.83	0.69	0.46	0.37	0.89
			Precision	[6,6]	0.85	0.78	0.50	0.37	0.90
				[7,7]	0.86	0.77	0.53	0.38	0.90
			Recall	[6,6]	0.84	0.68	0.46	0.37	0.90
				[7,7]	0.84	0.68	0.46	0.38	0.90
		RDP	F1-score	[6,6]	0.80	0.79	0.70	0.70	0.83
				[7,7]	0.80	0.79	0.72	0.70	0.83
			Precision	[6,6]	0.85	0.84	0.71	0.71	0.85
				[7,7]	0.85	0.85	0.72	0.71	0.85
			Recall	[6,6]	0.79	0.77	0.70	0.69	0.82
				[7,7]	0.79	0.78	0.71	0.70	0.82
		SILVA	F1-score	[6,6]	0.86	0.85	0.83	0.80	0.86
				[7,7]	0.87	0.87	0.86	0.84	0.86
			Precision	[6,6]	0.92	0.89	0.85	0.83	0.92
				[7,7]	0.92	0.93	0.92	0.86	0.92
			Recall	[6,6]	0.87	0.85	0.84	0.80	0.87
				[7,7]	0.87	0.88	0.87	0.85	0.87
	V4	GSR	F1-score	[6,6]	0.96	0.90	0.86	0.81	0.96
				[7,7]	0.96	0.91	0.88	0.81	0.96
			Precision	[6,6]	0.96	0.90	0.86	0.81	0.96
				[7,7]	0.96	0.91	0.88	0.81	0.96
			Recall	[6,6]	0.96	0.90	0.86	0.81	0.96
				[7,7]	0.96	0.91	0.88	0.81	0.96
		GTDB	F1-score	[6,6]	0.80	0.80	0.72	0.58	0.88
				[7,7]	0.80	0.77	0.72	0.58	0.88
			Precision	[6,6]	0.81	0.81	0.72	0.59	0.89
				[7,7]	0.81	0.78	0.72	0.59	0.89
			Recall	[6,6]	0.79	0.79	0.71	0.58	0.88
				[7,7]	0.79	0.77	0.71	0.58	0.88
		Greengenes	F1-score	[6,6]	0.85	0.80	0.66	0.66	0.89
				[7,7]	0.85	0.81	0.71	0.67	0.89
			Precision	[6,6]	0.85	0.80	0.65	0.65	0.88
				[7,7]	0.85	0.81	0.70	0.66	0.88

Continued on next page

Supplementary Table 3: N-gram-range and confidence threshold benchmarking at genus level. F1-score, precision and recall are shown for each database, evaluated region and validation datasets. The values are the average of the five metagenomic samples. Bold represents the highest value.

Validation Dataset	Region	Database	Metrics	Conf. thres. N-gram	**0.5**	**0.7**	**0.9**	**0.98**	**disable**
			Recall	[6,6]	0.86	0.81	0.67	0.67	0.90
				[7,7]	0.86	0.83	0.72	0.68	0.90
		ITGDB	F1-score	[6,6]	0.96	0.76	0.50	0.22	0.97
				[7,7]	0.95	0.76	0.50	0.37	0.99
			Precision	[6,6]	0.96	0.77	0.50	0.22	0.97
				[7,7]	0.95	0.76	0.50	0.37	0.99
			Recall	[6,6]	0.96	0.75	0.50	0.22	0.97
				[7,7]	0.95	0.76	0.50	0.37	0.99
		RDP	F1-score	[6,6]	0.75	0.72	0.63	0.60	0.79
				[7,7]	0.75	0.72	0.62	0.62	0.79
			Precision	[6,6]	0.75	0.72	0.63	0.60	0.79
				[7,7]	0.75	0.72	0.62	0.62	0.79
			Recall	[6,6]	0.75	0.72	0.63	0.60	0.79
				[7,7]	0.75	0.72	0.62	0.62	0.79
		SILVA	F1-score	[6,6]	0.95	0.92	0.80	0.78	0.98
				[7,7]	0.95	0.92	0.83	0.78	0.98
			Precision	[6,6]	0.95	0.92	0.79	0.77	0.98
				[7,7]	0.95	0.92	0.82	0.77	0.98
			Recall	[6,6]	0.95	0.92	0.83	0.81	0.98
				[7,7]	0.95	0.92	0.85	0.81	0.98
	full-16S	GSR	F1-score	[6,6]	0.84	0.83	0.83	0.81	0.85
				[7,7]	0.84	0.83	0.83	0.82	0.86
			Precision	[6,6]	0.85	0.82	0.82	0.81	0.87
				[7,7]	0.84	0.82	0.82	0.82	0.87
			Recall	[6,6]	0.85	0.84	0.84	0.83	0.87
				[7,7]	0.86	0.84	0.84	0.84	0.87
		GTDB	F1-score	[6,6]	0.75	0.73	0.69	0.62	0.79
				[7,7]	0.76	0.73	0.73	0.68	0.79
			Precision	[6,6]	0.80	0.76	0.72	0.63	0.82
				[7,7]	0.78	0.76	0.76	0.72	0.82
			Recall	[6,6]	0.76	0.74	0.70	0.63	0.80
				[7,7]	0.77	0.74	0.74	0.69	0.80
		Greengenes	F1-score	[6,6]	0.77	0.75	0.73	0.66	0.77
				[7,7]	0.79	0.78	0.76	0.75	0.79
			Precision	[6,6]	0.79	0.76	0.74	0.68	0.78
				[7,7]	0.80	0.80	0.76	0.76	0.80
			Recall	[6,6]	0.79	0.78	0.74	0.66	0.79
				[7,7]	0.81	0.80	0.78	0.77	0.81
		ITGDB	F1-score	[6,6]	0.88	0.80	0.58	0.40	0.89
				[7,7]	0.87	0.82	0.62	0.48	0.89
			Precision	[6,6]	0.90	0.87	0.68	0.48	0.91
				[7,7]	0.90	0.87	0.76	0.58	0.90
			Recall	[6,6]	0.89	0.79	0.57	0.39	0.90
				[7,7]	0.88	0.80	0.59	0.47	0.90
		RDP	F1-score	[6,6]	0.83	0.82	0.79	0.76	0.83
				[7,7]	0.83	0.83	0.82	0.79	0.83
			Precision	[6,6]	0.86	0.85	0.82	0.78	0.86
				[7,7]	0.86	0.86	0.86	0.80	0.85
			Recall	[6,6]	0.82	0.81	0.78	0.75	0.82
				[7,7]	0.82	0.82	0.80	0.78	0.83
		SILVA	F1-score	[6,6]	0.87	0.90	0.88	0.84	0.86
				[7,7]	0.87	0.87	0.93	0.89	0.87
			Precision	[6,6]	0.93	0.95	0.89	0.86	0.92
				[7,7]	0.93	0.93	0.95	0.93	0.92
			Recall	[6,6]	0.88	0.90	0.89	0.85	0.87
				[7,7]	0.88	0.88	0.92	0.89	0.88
		GSR	F1-score	[6,6]	0.98	0.98	0.98	0.98	0.98
				[7,7]	0.98	0.98	0.98	0.98	0.98
			Precision	[6,6]	**1.00**	**1.00**	**1.00**	**1.00**	**1.00**
				[7,7]	**1.00**	**1.00**	**1.00**	**1.00**	**1.00**
			Recall	[6,6]	0.95	0.95	0.95	0.95	0.95

Continued on next page

Supplementary Table 3: N-gram-range and confidence threshold benchmarking at genus level. F1-score, precision and recall are shown for each database, evaluated region and validation datasets. The values are the average of the five metagenomic samples. Bold represents the highest value.

Validation Dataset	Region	Database	Metrics	Conf. thres. N-gram	**0.5**	**0.7**	**0.9**	**0.98**	**disable**
				[7,7]	0.95	0.95	0.95	0.95	0.95
		GTDB	F1-score	[6,6]	0.89	0.89	0.89	0.89	0.89
				[7,7]	0.89	0.89	0.89	0.89	0.89
			Precision	[6,6]	0.93	0.93	0.93	0.93	0.93
				[7,7]	0.93	0.93	0.93	0.93	0.93
			Recall	[6,6]	0.85	0.85	0.85	0.85	0.85
				[7,7]	0.85	0.85	0.85	0.85	0.85
		Greengenes	F1-score	[6,6]	0.94	0.94	0.94	0.94	0.94
				[7,7]	0.95	0.94	0.94	0.94	0.95
			Precision	[6,6]	0.97	0.97	0.97	0.97	0.97
				[7,7]	**1.00**	0.97	0.97	0.97	**1.00**
			Recall	[6,6]	0.92	0.92	0.92	0.92	0.92
				[7,7]	0.93	0.92	0.92	0.92	0.93
		ITGDB	F1-score	[6,6]	0.95	0.94	0.93	0.93	0.95
				[7,7]	0.95	0.95	0.93	0.93	0.95
			Precision	[6,6]	**1.00**	**1.00**	**1.00**	**1.00**	**1.00**
				[7,7]	**1.00**	**1.00**	**1.00**	**1.00**	**1.00**
			Recall	[6,6]	0.90	0.89	0.88	0.88	0.91
				[7,7]	0.90	0.90	0.88	0.88	0.91
		RDP	F1-score	[6,6]	0.94	0.94	0.94	0.94	0.94
				[7,7]	0.94	0.94	0.94	0.94	0.94
			Precision	[6,6]	0.99	0.99	0.99	0.99	0.99
				[7,7]	0.99	0.99	0.99	0.99	0.99
			Recall	[6,6]	0.90	0.90	0.90	0.90	0.90
				[7,7]	0.90	0.90	0.90	0.90	0.90
		SILVA	F1-score	[6,6]	0.95	0.95	0.95	**1.00**	0.95
				[7,7]	0.95	0.95	0.95	0.95	0.95
			Precision	[6,6]	**1.00**	**1.00**	**1.00**	**1.00**	**1.00**
				[7,7]	**1.00**	**1.00**	**1.00**	**1.00**	**1.00**
			Recall	[6,6]	0.90	0.90	0.90	0.99	0.90
				[7,7]	0.90	0.90	0.90	0.90	0.90
		GSR	F1-score	[6,6]	0.97	0.97	0.97	0.97	0.97
				[7,7]	0.99	0.99	0.97	0.97	0.99
			Precision	[6,6]	**1.00**	**1.00**	**1.00**	**1.00**	**1.00**
				[7,7]	**1.00**	**1.00**	**1.00**	**1.00**	**1.00**
			Recall	[6,6]	0.94	0.94	0.94	0.94	0.94
				[7,7]	0.97	0.97	0.94	0.94	0.97
		GTDB	F1-score	[6,6]	0.92	0.89	0.89	0.84	0.92
				[7,7]	0.92	0.92	0.92	0.89	0.92
			Precision	[6,6]	0.95	0.95	0.95	0.95	0.95
				[7,7]	0.95	0.95	0.95	0.95	0.95
			Recall	[6,6]	0.90	0.84	0.84	0.76	0.90
				[7,7]	0.90	0.90	0.90	0.84	0.90
		Greengenes	F1-score	[6,6]	0.96	0.96	0.94	0.94	0.96
				[7,7]	0.96	0.96	0.96	0.96	0.96
			Precision	[6,6]	0.97	0.97	0.97	0.97	0.97
				[7,7]	0.97	0.97	0.97	0.97	0.97
			Recall	[6,6]	0.95	0.95	0.92	0.92	0.95
				[7,7]	0.95	0.95	0.95	0.95	0.95
	V3-V4	ITGDB	F1-score	[6,6]	0.96	0.96	0.96	0.95	0.97
				[7,7]	0.96	0.96	0.96	0.96	0.97
			Precision	[6,6]	**1.00**	0.99	0.99	0.98	**1.00**
				[7,7]	**1.00**	0.99	0.99	0.99	**1.00**
			Recall	[6,6]	0.93	0.93	0.93	0.91	0.94
				[7,7]	0.93	0.93	0.93	0.93	0.94
		RDP	F1-score	[6,6]	0.97	0.97	0.97	0.97	0.97
				[7,7]	0.97	0.97	0.97	0.97	0.97
			Precision	[6,6]	**1.00**	**1.00**	**1.00**	**1.00**	**1.00**
				[7,7]	**1.00**	**1.00**	**1.00**	**1.00**	**1.00**
			Recall	[6,6]	0.94	0.94	0.94	0.94	0.94
				[7,7]	0.94	0.94	0.94	0.94	0.94

Continued on next page

Supplementary Table 3: N-gram-range and confidence threshold benchmarking at genus level. F1-score, precision and recall are shown for each database, evaluated region and validation datasets. The values are the average of the five metagenomic samples. Bold represents the highest value.

Validation Dataset	Region	Database	Metrics	Conf. thres. / N-gram	**0.5**	**0.7**	**0.9**	**0.98**	**disable**
		SILVA	F1-score	[6,6]	**1.00**	0.99	0.99	0.99	0.97
				[7,7]	0.97	**1.00**	0.99	0.99	0.97
			Precision	[6,6]	**1.00**	0.99	0.99	0.99	**1.00**
				[7,7]	**1.00**	**1.00**	0.99	0.99	**1.00**
			Recall	[6,6]	**1.00**	0.99	0.99	0.99	0.94
				[7,7]	0.94	**1.00**	0.99	0.99	0.94
	V3-V5	GSR	F1-score	[6,6]	0.99	0.98	0.98	0.98	0.99
				[7,7]	0.99	0.99	0.98	0.98	0.99
			Precision	[6,6]	**1.00**	**1.00**	**1.00**	**1.00**	**1.00**
				[7,7]	**1.00**	**1.00**	**1.00**	**1.00**	**1.00**
			Recall	[6,6]	0.98	0.96	0.96	0.96	0.98
				[7,7]	0.98	0.98	0.96	0.96	0.98
		GTDB	F1-score	[6,6]	0.91	0.91	0.88	0.86	0.91
				[7,7]	0.91	0.91	0.91	0.91	0.91
			Precision	[6,6]	0.93	0.93	0.93	0.93	0.93
				[7,7]	0.93	0.93	0.93	0.93	0.93
			Recall	[6,6]	0.88	0.88	0.84	0.79	0.88
				[7,7]	0.88	0.88	0.88	0.88	0.88
		Greengenes	F1-score	[6,6]	0.95	0.95	0.95	0.94	0.95
				[7,7]	0.95	0.95	0.95	0.95	0.95
			Precision	[6,6]	0.97	0.97	0.97	0.97	0.97
				[7,7]	0.97	0.97	0.97	0.97	0.97
			Recall	[6,6]	0.94	0.94	0.94	0.92	0.94
				[7,7]	0.94	0.94	0.94	0.94	0.94
		ITGDB	F1-score	[6,6]	0.97	0.96	0.95	0.94	0.98
				[7,7]	0.97	0.97	0.95	0.94	0.98
			Precision	[6,6]	**1.00**	**1.00**	**1.00**	**1.00**	**1.00**
				[7,7]	**1.00**	**1.00**	**1.00**	**1.00**	**1.00**
			Recall	[6,6]	0.95	0.94	0.91	0.91	0.96
				[7,7]	0.94	0.94	0.91	0.91	0.96
		RDP	F1-score	[6,6]	0.97	0.97	0.97	0.97	0.97
				[7,7]	0.97	0.97	0.97	0.97	0.97
			Precision	[6,6]	**1.00**	**1.00**	**1.00**	**1.00**	**1.00**
				[7,7]	**1.00**	**1.00**	**1.00**	**1.00**	**1.00**
			Recall	[6,6]	0.94	0.94	0.94	0.94	0.94
				[7,7]	0.94	0.94	0.94	0.94	0.94
		SILVA	F1-score	[6,6]	**1.00**	0.99	0.99	0.99	**1.00**
				[7,7]	0.98	**1.00**	0.99	0.99	0.98
			Precision	[6,6]	**1.00**	**1.00**	0.99	0.99	**1.00**
				[7,7]	**1.00**	**1.00**	**1.00**	0.99	**1.00**
			Recall	[6,6]	**1.00**	0.99	0.99	0.99	**1.00**
				[7,7]	0.95	**1.00**	0.99	0.99	0.95
	V4	GSR	F1-score	[6,6]	0.96	0.96	0.96	0.96	0.96
				[7,7]	0.98	0.96	0.96	0.96	0.98
			Precision	[6,6]	**1.00**	**1.00**	**1.00**	**1.00**	**1.00**
				[7,7]	**1.00**	**1.00**	**1.00**	**1.00**	**1.00**
			Recall	[6,6]	0.93	0.93	0.93	0.93	0.93
				[7,7]	0.95	0.93	0.93	0.93	0.95
		GTDB	F1-score	[6,6]	0.84	0.80	0.80	0.79	0.84
				[7,7]	0.87	0.84	0.79	0.79	0.90
			Precision	[6,6]	0.93	0.93	0.93	0.93	0.93
				[7,7]	0.93	0.93	0.93	0.93	0.93
			Recall	[6,6]	0.76	0.71	0.71	0.69	0.76
				[7,7]	0.81	0.76	0.69	0.69	0.86
		Greengenes	F1-score	[6,6]	0.93	0.93	0.90	0.89	0.95
				[7,7]	0.93	0.93	0.90	0.90	0.95
			Precision	[6,6]	0.97	0.97	0.97	0.96	0.97
				[7,7]	0.97	0.97	0.97	0.96	0.97
			Recall	[6,6]	0.91	0.91	0.86	0.84	0.93
				[7,7]	0.91	0.91	0.86	0.85	0.93
		ITGDB	F1-score	[6,6]	0.96	0.92	0.93	0.91	0.96

Continued on next page

Supplementary Table 3: N-gram-range and confidence threshold benchmarking at genus level. F1-score, precision and recall are shown for each database, evaluated region and validation datasets. The values are the average of the five metagenomic samples. Bold represents the highest value.

Validation Dataset	Region	Database	Metrics	Conf. thres.<br>N-gram	**0.5**	**0.7**	**0.9**	**0.98**	**disable**
				[7,7]	0.96	0.92	0.93	0.92	0.96
			Precision	[6,6]	**1.00**	0.99	0.99	0.99	**1.00**
				[7,7]	**1.00**	0.99	0.99	0.99	**1.00**
			Recall	[6,6]	0.92	0.87	0.89	0.87	0.93
				[7,7]	0.92	0.87	0.89	0.87	0.93
		RDP	F1-score	[6,6]	0.93	0.93	0.90	0.87	0.96
				[7,7]	0.93	0.93	0.90	0.90	0.96
			Precision	[6,6]	**1.00**	**1.00**	**1.00**	**1.00**	**1.00**
				[7,7]	**1.00**	**1.00**	**1.00**	**1.00**	**1.00**
			Recall	[6,6]	0.88	0.88	0.83	0.77	0.93
				[7,7]	0.88	0.88	0.83	0.83	0.93
		SILVA	F1-score	[6,6]	0.95	0.95	0.99	0.96	0.96
				[7,7]	0.95	0.95	0.99	0.94	0.96
			Precision	[6,6]	0.99	0.99	0.99	0.99	**1.00**
				[7,7]	0.99	0.99	0.99	0.99	**1.00**
			Recall	[6,6]	0.92	0.92	0.99	0.94	0.92
				[7,7]	0.92	0.92	0.99	0.89	0.92
	full-16S	GSR	F1-score	[6,6]	0.98	0.98	0.98	0.98	0.98
				[7,7]	0.98	0.98	0.98	0.98	0.98
			Precision	[6,6]	**1.00**	**1.00**	**1.00**	**1.00**	**1.00**
				[7,7]	**1.00**	**1.00**	**1.00**	**1.00**	**1.00**
			Recall	[6,6]	0.96	0.96	0.96	0.96	0.96
				[7,7]	0.96	0.96	0.96	0.96	0.96
		GTDB	F1-score	[6,6]	0.89	0.89	0.87	0.84	0.89
				[7,7]	0.91	0.91	0.91	0.89	0.91
			Precision	[6,6]	0.92	0.92	0.92	0.92	0.92
				[7,7]	0.92	0.92	0.92	0.92	0.92
			Recall	[6,6]	0.86	0.86	0.82	0.78	0.86
				[7,7]	0.89	0.89	0.89	0.86	0.89
		Greengenes	F1-score	[6,6]	0.96	0.96	0.95	0.94	0.96
				[7,7]	0.96	0.96	0.96	0.96	0.96
			Precision	[6,6]	**1.00**	**1.00**	0.97	0.97	**1.00**
				[7,7]	**1.00**	**1.00**	**1.00**	**1.00**	**1.00**
			Recall	[6,6]	0.94	0.94	0.93	0.91	0.94
				[7,7]	0.96	0.96	0.96	0.96	0.96
		ITGDB	F1-score	[6,6]	0.98	0.98	0.96	0.95	0.98
				[7,7]	0.98	0.98	0.96	0.96	0.98
			Precision	[6,6]	**1.00**	**1.00**	**1.00**	**1.00**	**1.00**
				[7,7]	**1.00**	**1.00**	**1.00**	**1.00**	**1.00**
			Recall	[6,6]	0.96	0.96	0.94	0.92	0.96
				[7,7]	0.96	0.96	0.94	0.93	0.96
		RDP	F1-score	[6,6]	0.98	0.98	0.98	0.98	0.98
				[7,7]	0.98	0.98	0.98	0.98	0.98
			Precision	[6,6]	**1.00**	**1.00**	**1.00**	**1.00**	**1.00**
				[7,7]	**1.00**	**1.00**	**1.00**	**1.00**	**1.00**
			Recall	[6,6]	0.96	0.96	0.96	0.96	0.96
				[7,7]	0.96	0.96	0.96	0.96	0.96
		SILVA	F1-score	[6,6]	0.98	0.98	0.98	0.98	0.98
				[7,7]	0.98	0.98	0.98	0.98	0.98
			Precision	[6,6]	**1.00**	**1.00**	**1.00**	**1.00**	**1.00**
				[7,7]	**1.00**	**1.00**	**1.00**	**1.00**	**1.00**
			Recall	[6,6]	0.96	0.96	0.96	0.96	0.96
				[7,7]	0.96	0.96	0.96	0.96	0.96

Supplementary Table 4: N-gram-range and confidence threshold benchmarking at species level. F1-score, precision and recall are shown for each database, evaluated region and validation datasets. The values are the average of the five metagenomic samples. Bold represents the highest value.

Validation Dataset	Region	Database	Metrics	Conf. thres. / N-gram	**0.5**	**0.7**	**0.9**	**0.98**	**disable**
	V1-V3	GSR	F1-score	[6,6]	0.96	0.95	0.93	0.93	0.96
				[7,7]	0.99	0.98	0.94	0.94	0.99
			Precision	[6,6]	0.96	0.97	0.95	0.94	0.96
				[7,7]	0.99	0.99	0.96	0.95	0.99
			Recall	[6,6]	0.95	0.95	0.93	0.92	0.95
				[7,7]	0.98	0.98	0.94	0.93	0.98
		GTDB	F1-score	[6,6]	0.80	0.74	0.69	0.60	0.81
				[7,7]	0.80	0.77	0.71	0.63	0.80
			Precision	[6,6]	0.85	0.83	0.75	0.71	0.85
				[7,7]	0.85	0.84	0.82	0.74	0.85
			Recall	[6,6]	0.79	0.72	0.66	0.57	0.80
				[7,7]	0.77	0.74	0.68	0.60	0.78
		Greengenes	F1-score	[6,6]	0.56	0.55	0.53	0.51	0.57
				[7,7]	0.57	0.56	0.55	0.53	0.57
			Precision	[6,6]	0.59	0.59	0.58	0.58	0.59
				[7,7]	0.60	0.60	0.59	0.58	0.60
			Recall	[6,6]	0.55	0.53	0.52	0.50	0.55
				[7,7]	0.55	0.54	0.53	0.51	0.56
		ITGDB	F1-score	[6,6]	0.76	0.75	0.70	0.65	0.85
				[7,7]	0.76	0.75	0.71	0.65	0.88
			Precision	[6,6]	0.77	0.76	0.76	0.74	0.86
				[7,7]	0.77	0.77	0.76	0.74	0.88
			Recall	[6,6]	0.77	0.74	0.68	0.62	0.86
				[7,7]	0.77	0.73	0.68	0.62	0.88
		RDP	F1-score	[6,6]	0.60	0.60	0.61	0.61	0.59
				[7,7]	0.59	0.60	0.61	0.61	0.59
			Precision	[6,6]	0.60	0.60	0.61	0.62	0.59
				[7,7]	0.59	0.60	0.61	0.61	0.59
			Recall	[6,6]	0.61	0.61	0.61	0.60	0.61
				[7,7]	0.61	0.61	0.61	0.60	0.61
		SILVA	F1-score	[6,6]	0.65	0.60	0.54	0.44	0.72
				[7,7]	0.71	0.66	0.57	0.54	0.74
			Precision	[6,6]	0.77	0.75	0.66	0.50	0.82
				[7,7]	0.83	0.79	0.72	0.60	0.83
			Recall	[6,6]	0.63	0.58	0.53	0.42	0.70
				[7,7]	0.69	0.64	0.56	0.52	0.71
	V3-V4	GSR	F1-score	[6,6]	0.89	0.85	0.79	0.74	0.93
				[7,7]	0.89	0.86	0.85	0.79	0.99
			Precision	[6,6]	0.90	0.86	0.81	0.76	0.94
				[7,7]	0.89	0.87	0.86	0.81	0.99
			Recall	[6,6]	0.89	0.85	0.79	0.74	0.92
				[7,7]	0.88	0.85	0.85	0.79	0.98
		GTDB	F1-score	[6,6]	0.72	0.61	0.52	0.34	0.76
				[7,7]	0.71	0.64	0.53	0.35	0.75
			Precision	[6,6]	0.74	0.67	0.61	0.39	0.78
				[7,7]	0.75	0.70	0.63	0.37	0.78
			Recall	[6,6]	0.71	0.60	0.50	0.33	0.76
				[7,7]	0.70	0.63	0.52	0.34	0.74
		Greengenes	F1-score	[6,6]	0.52	0.42	0.40	0.31	0.53
				[7,7]	0.54	0.46	0.43	0.35	0.55
			Precision	[6,6]	0.53	0.45	0.43	0.34	0.54
				[7,7]	0.55	0.49	0.47	0.41	0.56
			Recall	[6,6]	0.52	0.42	0.39	0.30	0.52
				[7,7]	0.53	0.45	0.42	0.34	0.54
		ITGDB	F1-score	[6,6]	0.66	0.63	0.50	0.42	0.86
				[7,7]	0.69	0.64	0.51	0.44	0.92
			Precision	[6,6]	0.67	0.65	0.53	0.46	0.87
				[7,7]	0.70	0.66	0.54	0.48	0.92
			Recall	[6,6]	0.65	0.62	0.48	0.41	0.86
				[7,7]	0.69	0.63	0.50	0.43	0.91

Continued on next page

Supplementary Table 4: Supplementary table 4. N-gram-range and confidence threshold benchmarking at species level. F1-score, precision and recall are shown for each database, evaluated region and validation datasets. The values are the average of the five metagenomic samples. Bold represents the highest value.

Validation Dataset	Region	Database	Metrics	Conf. thres. N-gram	**0.5**	**0.7**	**0.9**	**0.98**	**disable**
		RDP	F1-score	[6,6]	0.80	0.73	0.61	0.51	0.88
				[7,7]	0.74	0.70	0.62	0.59	0.82
			Precision	[6,6]	0.88	0.81	0.62	0.53	0.89
				[7,7]	0.82	0.78	0.64	0.60	0.87
			Recall	[6,6]	0.78	0.70	0.60	0.51	0.88
				[7,7]	0.71	0.68	0.62	0.58	0.81
		SILVA	F1-score	[6,6]	0.37	0.34	0.28	0.15	0.47
				[7,7]	0.42	0.38	0.34	0.30	0.50
			Precision	[6,6]	0.40	0.35	0.30	0.20	0.52
				[7,7]	0.47	0.40	0.36	0.32	0.56
			Recall	[6,6]	0.36	0.33	0.27	0.12	0.44
				[7,7]	0.40	0.37	0.34	0.29	0.47
	V3-V5	GSR	F1-score	[6,6]	0.94	0.92	0.89	0.82	0.94
				[7,7]	0.95	0.95	0.93	0.87	0.95
			Precision	[6,6]	0.95	0.94	0.91	0.86	0.95
				[7,7]	0.96	0.96	0.95	0.91	0.96
			Recall	[6,6]	0.94	0.92	0.89	0.80	0.94
				[7,7]	0.95	0.94	0.92	0.85	0.95
		GTDB	F1-score	[6,6]	0.68	0.56	0.47	0.38	0.74
				[7,7]	0.63	0.56	0.51	0.41	0.69
			Precision	[6,6]	0.73	0.66	0.59	0.46	0.78
				[7,7]	0.69	0.67	0.61	0.53	0.72
			Recall	[6,6]	0.66	0.54	0.46	0.36	0.73
				[7,7]	0.61	0.54	0.49	0.39	0.68
		Greengenes	F1-score	[6,6]	0.53	0.48	0.42	0.36	0.54
				[7,7]	0.55	0.52	0.45	0.41	0.56
			Precision	[6,6]	0.55	0.53	0.48	0.45	0.56
				[7,7]	0.57	0.55	0.50	0.48	0.58
			Recall	[6,6]	0.52	0.46	0.40	0.33	0.53
				[7,7]	0.54	0.50	0.44	0.39	0.55
		ITGDB	F1-score	[6,6]	0.72	0.68	0.57	0.48	0.86
				[7,7]	0.73	0.70	0.61	0.51	0.87
			Precision	[6,6]	0.73	0.72	0.63	0.57	0.87
				[7,7]	0.74	0.72	0.70	0.62	0.88
			Recall	[6,6]	0.71	0.66	0.55	0.45	0.86
				[7,7]	0.72	0.68	0.58	0.47	0.87
		RDP	F1-score	[6,6]	0.74	0.73	0.67	0.60	0.77
				[7,7]	0.73	0.74	0.69	0.67	0.77
			Precision	[6,6]	0.84	0.83	0.70	0.62	0.83
				[7,7]	0.82	0.84	0.71	0.70	0.83
			Recall	[6,6]	0.72	0.71	0.66	0.59	0.76
				[7,7]	0.72	0.72	0.68	0.66	0.76
		SILVA	F1-score	[6,6]	0.44	0.37	0.30	0.21	0.56
				[7,7]	0.56	0.47	0.38	0.32	0.62
			Precision	[6,6]	0.59	0.49	0.39	0.28	0.71
				[7,7]	0.71	0.60	0.51	0.39	0.74
			Recall	[6,6]	0.41	0.35	0.29	0.19	0.53
				[7,7]	0.52	0.45	0.36	0.31	0.59
		GSR	F1-score	[6,6]	0.91	0.85	0.76	0.64	0.91
				[7,7]	0.91	0.89	0.79	0.72	0.92
			Precision	[6,6]	0.91	0.86	0.78	0.70	0.91
				[7,7]	0.92	0.90	0.80	0.75	0.93
			Recall	[6,6]	0.91	0.85	0.76	0.61	0.91
				[7,7]	0.91	0.89	0.78	0.70	0.92
		GTDB	F1-score	[6,6]	0.61	0.52	0.33	0.16	0.70
				[7,7]	0.58	0.53	0.35	0.23	0.68
			Precision	[6,6]	0.68	0.61	0.42	0.22	0.72
				[7,7]	0.66	0.62	0.43	0.31	0.70
			Recall	[6,6]	0.59	0.50	0.32	0.14	0.69
				[7,7]	0.56	0.51	0.33	0.21	0.68
		Greengenes	F1-score	[6,6]	0.50	0.44	0.38	0.30	0.53

Continued on next page

Supplementary Table 4: Supplementary table 4. N-gram-range and confidence threshold benchmarking at species level. F1-score, precision and recall are shown for each database, evaluated region and validation datasets. The values are the average of the five metagenomic samples. Bold represents the highest value.

Validation Dataset	Region	Database	Metrics	Conf. thres. N-gram	**0.5**	**0.7**	**0.9**	**0.98**	**disable**
				[7,7]	0.51	0.45	0.42	0.33	0.54
			Precision	[6,6]	0.53	0.48	0.40	0.35	0.55
				[7,7]	0.54	0.49	0.44	0.37	0.56
			Recall	[6,6]	0.49	0.44	0.37	0.28	0.52
				[7,7]	0.51	0.44	0.41	0.32	0.53
		ITGDB	F1-score	[6,6]	0.61	0.57	0.44	0.29	0.84
				[7,7]	0.61	0.57	0.45	0.35	0.84
			Precision	[6,6]	0.62	0.59	0.49	0.41	0.85
				[7,7]	0.61	0.60	0.49	0.46	0.86
			Recall	[6,6]	0.61	0.55	0.43	0.26	0.83
				[7,7]	0.60	0.56	0.43	0.31	0.84
		RDP	F1-score	[6,6]	0.85	0.72	0.56	0.50	0.87
				[7,7]	0.85	0.84	0.58	0.52	0.87
			Precision	[6,6]	0.85	0.79	0.57	0.51	0.88
				[7,7]	0.86	0.85	0.59	0.53	0.88
			Recall	[6,6]	0.84	0.70	0.56	0.49	0.87
				[7,7]	0.85	0.84	0.58	0.51	0.87
		SILVA	F1-score	[6,6]	0.34	0.27	0.13	0.05	0.44
				[7,7]	0.40	0.34	0.23	0.09	0.49
			Precision	[6,6]	0.38	0.29	0.20	0.06	0.50
				[7,7]	0.46	0.37	0.27	0.10	0.57
			Recall	[6,6]	0.32	0.26	0.11	0.05	0.43
				[7,7]	0.38	0.33	0.21	0.08	0.46
	full-16S	GSR	F1-score	[6,6]	0.99	0.99	0.98	0.94	0.99
				[7,7]	**1.00**	**1.00**	0.99	0.98	**1.00**
			Precision	[6,6]	**1.00**	**1.00**	0.98	0.95	0.99
				[7,7]	**1.00**	**1.00**	**1.00**	0.99	**1.00**
			Recall	[6,6]	0.99	0.98	0.97	0.93	0.99
				[7,7]	0.99	0.99	0.99	0.97	0.99
		GTDB	F1-score	[6,6]	0.52	0.36	0.30	0.21	0.58
				[7,7]	0.47	0.44	0.40	0.36	0.47
			Precision	[6,6]	0.59	0.44	0.37	0.29	0.65
				[7,7]	0.55	0.53	0.48	0.45	0.55
			Recall	[6,6]	0.48	0.34	0.29	0.20	0.55
				[7,7]	0.45	0.42	0.38	0.35	0.45
		Greengenes	F1-score	[6,6]	0.55	0.52	0.48	0.43	0.56
				[7,7]	0.56	0.55	0.53	0.50	0.57
			Precision	[6,6]	0.59	0.58	0.56	0.48	0.59
				[7,7]	0.60	0.60	0.59	0.57	0.60
			Recall	[6,6]	0.53	0.50	0.47	0.41	0.54
				[7,7]	0.55	0.53	0.51	0.48	0.55
		ITGDB	F1-score	[6,6]	0.84	0.80	0.70	0.55	0.91
				[7,7]	0.84	0.81	0.72	0.63	0.91
			Precision	[6,6]	0.84	0.84	0.80	0.67	0.91
				[7,7]	0.84	0.84	0.82	0.76	0.91
			Recall	[6,6]	0.84	0.78	0.66	0.51	0.91
				[7,7]	0.84	0.80	0.67	0.58	0.91
		RDP	F1-score	[6,6]	0.87	0.87	0.84	0.75	0.88
				[7,7]	0.88	0.88	0.87	0.84	0.88
			Precision	[6,6]	0.88	0.88	0.88	0.87	0.88
				[7,7]	0.89	0.89	0.88	0.88	0.89
			Recall	[6,6]	0.87	0.87	0.81	0.71	0.88
				[7,7]	0.88	0.88	0.87	0.81	0.88
		SILVA	F1-score	[6,6]	0.71	0.68	0.54	0.35	0.73
				[7,7]	0.76	0.74	0.71	0.66	0.77
			Precision	[6,6]	0.87	0.85	0.73	0.45	0.88
				[7,7]	0.90	0.89	0.88	0.84	0.90
			Recall	[6,6]	0.69	0.66	0.50	0.33	0.71
				[7,7]	0.74	0.72	0.69	0.62	0.74
			F1-score	[6,6]	0.85	0.82	0.76	0.62	0.87
				[7,7]	0.85	0.84	0.79	0.68	0.87
		GSR							

Continued on next page

Supplementary Table 4: Supplementary table 4. N-gram-range and confidence threshold benchmarking at species level. F1-score, precision and recall are shown for each database, evaluated region and validation datasets. The values are the average of the five metagenomic samples. Bold represents the highest value.

Validation Dataset	Region	Database	Metrics	Conf. thres. N-gram	**0.5**	**0.7**	**0.9**	**0.98**	**disable**
			Precision	[6,6]	0.85	0.83	0.79	0.67	0.91
				[7,7]	0.85	0.85	0.81	0.73	0.91
			Recall	[6,6]	0.84	0.81	0.74	0.60	0.86
				[7,7]	0.84	0.84	0.78	0.66	0.86
			F1-score	[6,6]	0.78	0.69	0.58	0.45	0.82
				[7,7]	0.79	0.71	0.62	0.53	0.81
		GTDB	Precision	[6,6]	0.85	0.76	0.66	0.48	0.87
				[7,7]	0.86	0.78	0.69	0.57	0.87
			Recall	[6,6]	0.76	0.67	0.55	0.44	0.81
				[7,7]	0.77	0.69	0.59	0.51	0.79
			F1-score	[6,6]	0.49	0.47	0.42	0.40	0.50
				[7,7]	0.51	0.49	0.44	0.41	0.51
		Greengenes	Precision	[6,6]	0.53	0.52	0.44	0.42	0.54
				[7,7]	0.54	0.53	0.48	0.44	0.54
			Recall	[6,6]	0.48	0.46	0.41	0.39	0.49
				[7,7]	0.50	0.47	0.43	0.41	0.50
			F1-score	[6,6]	0.72	0.66	0.49	0.31	0.78
				[7,7]	0.72	0.67	0.52	0.34	0.78
		ITGDB	Precision	[6,6]	0.73	0.70	0.54	0.33	0.82
				[7,7]	0.73	0.70	0.56	0.38	0.81
			Recall	[6,6]	0.71	0.65	0.46	0.30	0.77
				[7,7]	0.72	0.66	0.50	0.33	0.78
			F1-score	[6,6]	0.65	0.62	0.61	0.48	0.65
				[7,7]	0.65	0.64	0.62	0.52	0.65
		RDP	Precision	[6,6]	0.69	0.68	0.66	0.59	0.69
				[7,7]	0.69	0.69	0.67	0.62	0.69
			Recall	[6,6]	0.63	0.60	0.58	0.45	0.63
				[7,7]	0.63	0.63	0.59	0.49	0.64
			F1-score	[6,6]	0.82	0.76	0.60	0.38	0.85
				[7,7]	0.83	0.80	0.71	0.55	0.85
		SILVA	Precision	[6,6]	0.83	0.78	0.65	0.46	0.89
				[7,7]	0.83	0.81	0.77	0.60	0.88
			Recall	[6,6]	0.81	0.75	0.58	0.36	0.84
				[7,7]	0.82	0.79	0.68	0.53	0.84
			F1-score	[6,6]	0.75	0.71	0.58	0.35	0.89
				[7,7]	0.75	0.71	0.60	0.40	0.84
		GSR	Precision	[6,6]	0.75	0.71	0.58	0.35	0.89
				[7,7]	0.75	0.71	0.60	0.40	0.84
			Recall	[6,6]	0.75	0.71	0.58	0.35	0.89
				[7,7]	0.75	0.71	0.60	0.40	0.84
			F1-score	[6,6]	0.66	0.64	0.24	0.15	0.75
				[7,7]	0.66	0.64	0.43	0.19	0.75
		GTDB	Precision	[6,6]	0.66	0.64	0.24	0.15	0.75
				[7,7]	0.66	0.64	0.43	0.19	0.75
			Recall	[6,6]	0.66	0.64	0.24	0.15	0.75
				[7,7]	0.66	0.64	0.43	0.19	0.75
			F1-score	[6,6]	0.45	0.42	0.27	0.26	0.59
				[7,7]	0.48	0.42	0.31	0.26	0.60
		Greengenes	Precision	[6,6]	0.45	0.42	0.27	0.26	0.59
				[7,7]	0.48	0.42	0.31	0.26	0.60
			Recall	[6,6]	0.45	0.42	0.27	0.26	0.59
	V3-V4			[7,7]	0.48	0.42	0.31	0.26	0.60
			F1-score	[6,6]	0.71	0.56	0.33	0.13	0.94
				[7,7]	0.71	0.60	0.33	0.22	0.86
		ITGDB	Precision	[6,6]	0.71	0.56	0.33	0.13	0.94
				[7,7]	0.71	0.60	0.33	0.22	0.86
			Recall	[6,6]	0.71	0.56	0.33	0.13	0.94
				[7,7]	0.71	0.60	0.33	0.22	0.86
			F1-score	[6,6]	0.43	0.43	0.26	0.17	0.51
				[7,7]	0.43	0.43	0.37	0.22	0.51
		RDP	Precision	[6,6]	0.43	0.43	0.26	0.17	0.51

Continued on next page

Supplementary Table 4: Supplementary table 4. N-gram-range and confidence threshold benchmarking at species level. F1-score, precision and recall are shown for each database, evaluated region and validation datasets. The values are the average of the five metagenomic samples. Bold represents the highest value.

Validation Dataset	Region	Database	Metrics	Conf. thres. N-gram	**0.5**	**0.7**	**0.9**	**0.98**	**disable**
				[7,7]	0.43	0.43	0.37	0.22	0.51
			Recall	[6,6]	0.43	0.43	0.26	0.17	0.51
				[7,7]	0.43	0.43	0.37	0.22	0.51
		SILVA	F1-score	[6,6]	0.68	0.41	0.24	0.07	0.88
				[7,7]	0.68	0.63	0.36	0.21	0.75
			Precision	[6,6]	0.68	0.41	0.24	0.07	0.88
				[7,7]	0.68	0.63	0.36	0.21	0.75
			Recall	[6,6]	0.68	0.41	0.24	0.07	0.88
				[7,7]	0.68	0.63	0.36	0.21	0.75
	V3-V5	GSR	F1-score	[6,6]	0.66	0.59	0.53	0.35	0.77
				[7,7]	0.67	0.64	0.56	0.40	0.78
			Precision	[6,6]	0.66	0.60	0.55	0.37	0.80
				[7,7]	0.67	0.64	0.57	0.42	0.81
			Recall	[6,6]	0.67	0.60	0.52	0.35	0.78
				[7,7]	0.69	0.65	0.57	0.40	0.79
		GTDB	F1-score	[6,6]	0.59	0.47	0.35	0.19	0.66
				[7,7]	0.59	0.49	0.39	0.25	0.65
			Precision	[6,6]	0.61	0.49	0.40	0.20	0.70
				[7,7]	0.62	0.51	0.43	0.26	0.67
			Recall	[6,6]	0.59	0.47	0.35	0.18	0.67
				[7,7]	0.60	0.49	0.38	0.26	0.66
		Greengenes	F1-score	[6,6]	0.37	0.33	0.28	0.19	0.47
				[7,7]	0.41	0.35	0.31	0.26	0.47
			Precision	[6,6]	0.40	0.34	0.30	0.21	0.51
				[7,7]	0.43	0.36	0.32	0.27	0.50
			Recall	[6,6]	0.37	0.34	0.27	0.19	0.47
				[7,7]	0.41	0.35	0.31	0.26	0.47
		ITGDB	F1-score	[6,6]	0.61	0.43	0.27	0.13	0.82
				[7,7]	0.63	0.46	0.34	0.15	0.79
			Precision	[6,6]	0.62	0.45	0.27	0.15	0.84
				[7,7]	0.66	0.46	0.34	0.17	0.83
			Recall	[6,6]	0.62	0.44	0.27	0.13	0.83
				[7,7]	0.64	0.47	0.36	0.14	0.80
		RDP	F1-score	[6,6]	0.53	0.53	0.38	0.29	0.60
				[7,7]	0.57	0.54	0.43	0.34	0.62
			Precision	[6,6]	0.61	0.60	0.39	0.31	0.64
				[7,7]	0.67	0.61	0.45	0.36	0.69
			Recall	[6,6]	0.51	0.50	0.37	0.28	0.58
				[7,7]	0.54	0.51	0.42	0.33	0.59
		SILVA	F1-score	[6,6]	0.53	0.42	0.25	0.16	0.72
				[7,7]	0.61	0.52	0.37	0.23	0.70
			Precision	[6,6]	0.53	0.45	0.27	0.18	0.75
				[7,7]	0.61	0.53	0.43	0.24	0.74
			Recall	[6,6]	0.54	0.40	0.25	0.14	0.73
				[7,7]	0.62	0.52	0.36	0.22	0.70
	V4	GSR	F1-score	[6,6]	0.68	0.50	0.35	0.14	0.78
				[7,7]	0.70	0.57	0.35	0.21	0.78
			Precision	[6,6]	0.68	0.50	0.35	0.14	0.78
				[7,7]	0.70	0.57	0.35	0.21	0.78
			Recall	[6,6]	0.68	0.50	0.35	0.14	0.78
				[7,7]	0.70	0.57	0.35	0.21	0.78
		GTDB	F1-score	[6,6]	0.49	0.45	0.20	0.08	0.54
				[7,7]	0.49	0.48	0.22	0.08	0.54
			Precision	[6,6]	0.49	0.45	0.20	0.08	0.54
				[7,7]	0.49	0.48	0.22	0.08	0.54
			Recall	[6,6]	0.49	0.45	0.20	0.08	0.54
				[7,7]	0.49	0.48	0.22	0.08	0.54
		Greengenes	F1-score	[6,6]	0.37	0.29	0.18	0.09	0.48
				[7,7]	0.37	0.29	0.21	0.11	0.48
			Precision	[6,6]	0.37	0.29	0.18	0.09	0.48
				[7,7]	0.37	0.29	0.21	0.11	0.48

Continued on next page

Supplementary Table 4: Supplementary table 4. N-gram-range and confidence threshold benchmarking at species level. F1-score, precision and recall are shown for each database, evaluated region and validation datasets. The values are the average of the five metagenomic samples. Bold represents the highest value.

Validation Dataset	Region	Database	Metrics	Conf. thres. N-gram	**0.5**	**0.7**	**0.9**	**0.98**	**disable**
			Recall	[6,6]	0.37	0.29	0.18	0.09	0.48
				[7,7]	0.37	0.29	0.21	0.11	0.48
		ITGDB	F1-score	[6,6]	0.42	0.26	0.16	0.01	0.89
				[7,7]	0.56	0.32	0.17	0.01	0.91
			Precision	[6,6]	0.42	0.26	0.16	0.01	0.89
				[7,7]	0.56	0.32	0.17	0.01	0.91
			Recall	[6,6]	0.42	0.26	0.16	0.01	0.89
				[7,7]	0.56	0.32	0.17	0.01	0.91
		RDP	F1-score	[6,6]	0.44	0.27	0.20	0.11	0.56
				[7,7]	0.44	0.40	0.20	0.11	0.56
			Precision	[6,6]	0.44	0.27	0.20	0.11	0.56
				[7,7]	0.44	0.40	0.20	0.11	0.56
			Recall	[6,6]	0.44	0.27	0.20	0.11	0.56
				[7,7]	0.44	0.40	0.20	0.11	0.56
		SILVA	F1-score	[6,6]	0.31	0.20	0.11	0.04	0.78
				[7,7]	0.38	0.25	0.13	0.04	0.79
			Precision	[6,6]	0.31	0.20	0.11	0.04	0.78
				[7,7]	0.38	0.25	0.13	0.04	0.79
			Recall	[6,6]	0.31	0.20	0.11	0.04	0.78
				[7,7]	0.38	0.25	0.13	0.04	0.79
	full-16S	GSR	F1-score	[6,6]	0.75	0.70	0.62	0.44	0.79
				[7,7]	0.74	0.71	0.66	0.60	0.77
			Precision	[6,6]	0.77	0.72	0.66	0.44	0.83
				[7,7]	0.75	0.73	0.66	0.62	0.79
			Recall	[6,6]	0.75	0.70	0.61	0.43	0.79
				[7,7]	0.75	0.71	0.67	0.59	0.77
		GTDB	F1-score	[6,6]	0.30	0.25	0.19	0.13	0.33
				[7,7]	0.32	0.29	0.24	0.20	0.35
			Precision	[6,6]	0.32	0.27	0.21	0.18	0.34
				[7,7]	0.34	0.31	0.25	0.23	0.37
			Recall	[6,6]	0.30	0.24	0.18	0.11	0.33
				[7,7]	0.31	0.28	0.23	0.19	0.34
		Greengenes	F1-score	[6,6]	0.49	0.42	0.36	0.25	0.50
				[7,7]	0.51	0.48	0.40	0.36	0.52
			Precision	[6,6]	0.51	0.45	0.38	0.28	0.53
				[7,7]	0.53	0.52	0.41	0.38	0.55
			Recall	[6,6]	0.49	0.42	0.35	0.25	0.50
				[7,7]	0.51	0.47	0.40	0.36	0.52
		ITGDB	F1-score	[6,6]	0.79	0.66	0.38	0.19	0.85
				[7,7]	0.80	0.71	0.54	0.32	0.84
			Precision	[6,6]	0.79	0.70	0.41	0.26	0.87
				[7,7]	0.82	0.75	0.60	0.36	0.86
			Recall	[6,6]	0.80	0.65	0.37	0.17	0.86
				[7,7]	0.81	0.71	0.52	0.30	0.86
		RDP	F1-score	[6,6]	0.72	0.64	0.54	0.42	0.76
				[7,7]	0.76	0.72	0.63	0.52	0.76
			Precision	[6,6]	0.79	0.69	0.59	0.50	0.79
				[7,7]	0.79	0.75	0.74	0.56	0.79
			Recall	[6,6]	0.69	0.62	0.52	0.40	0.75
				[7,7]	0.75	0.71	0.61	0.50	0.75
		SILVA	F1-score	[6,6]	0.77	0.70	0.49	0.30	0.80
				[7,7]	0.79	0.74	0.70	0.54	0.82
			Precision	[6,6]	0.78	0.73	0.53	0.31	0.82
				[7,7]	0.79	0.76	0.73	0.63	0.84
			Recall	[6,6]	0.77	0.69	0.47	0.29	0.81
				[7,7]	0.80	0.74	0.69	0.51	0.82
		GSR	F1-score	[6,6]	0.83	0.83	0.80	0.77	0.83
				[7,7]	0.83	0.83	0.80	0.77	0.83
			Precision	[6,6]	0.96	0.96	0.96	0.97	0.96
				[7,7]	0.96	0.96	0.96	0.97	0.96
			Recall	[6,6]	0.78	0.78	0.73	0.68	0.78

Continued on next page

Supplementary Table 4: Supplementary table 4. N-gram-range and confidence threshold benchmarking at species level. F1-score, precision and recall are shown for each database, evaluated region and validation datasets. The values are the average of the five metagenomic samples. Bold represents the highest value.

Validation Dataset	Region	Database	Metrics	Conf. thres. N-gram	**0.5**	**0.7**	**0.9**	**0.98**	**disable**
				[7,7]	0.78	0.78	0.73	0.69	0.78
		GTDB	F1-score	[6,6]	0.54	0.51	0.51	0.49	0.55
				[7,7]	0.54	0.52	0.51	0.49	0.55
			Precision	[6,6]	0.89	0.85	0.85	0.87	0.89
				[7,7]	0.89	0.89	0.85	0.87	0.89
			Recall	[6,6]	0.46	0.42	0.42	0.38	0.48
				[7,7]	0.46	0.43	0.42	0.38	0.47
		Greengenes	F1-score	[6,6]	0.53	0.53	0.52	0.52	0.52
				[7,7]	0.54	0.53	0.52	0.52	0.54
			Precision	[6,6]	0.70	0.70	0.70	0.70	0.70
				[7,7]	0.73	0.70	0.70	0.70	0.73
			Recall	[6,6]	0.50	0.49	0.48	0.47	0.50
				[7,7]	0.51	0.49	0.49	0.48	0.51
		ITGDB	F1-score	[6,6]	0.79	0.79	0.79	0.78	0.80
				[7,7]	0.80	0.80	0.79	0.79	0.81
			Precision	[6,6]	0.95	0.97	0.97	0.97	0.95
				[7,7]	0.97	0.97	0.97	0.97	0.97
			Recall	[6,6]	0.72	0.71	0.70	0.69	0.72
				[7,7]	0.72	0.72	0.70	0.69	0.72
		RDP	F1-score	[6,6]	0.30	0.32	0.29	0.29	0.30
				[7,7]	0.30	0.30	0.30	0.29	0.30
			Precision	[6,6]	0.27	0.30	0.31	0.31	0.27
				[7,7]	0.27	0.27	0.30	0.31	0.27
			Recall	[6,6]	0.35	0.35	0.28	0.28	0.35
				[7,7]	0.35	0.35	0.31	0.28	0.35
		SILVA	F1-score	[6,6]	0.33	0.32	0.30	0.30	0.33
				[7,7]	0.33	0.31	0.30	0.29	0.33
			Precision	[6,6]	0.32	0.32	0.31	0.33	0.32
				[7,7]	0.32	0.32	0.31	0.31	0.32
			Recall	[6,6]	0.37	0.36	0.33	0.32	0.37
				[7,7]	0.37	0.35	0.33	0.33	0.37
	V3-V4	GSR	F1-score	[6,6]	0.94	0.88	0.82	0.73	0.94
				[7,7]	0.96	0.92	0.85	0.76	0.96
			Precision	[6,6]	**1.00**	**1.00**	**1.00**	**1.00**	**1.00**
				[7,7]	**1.00**	**1.00**	**1.00**	**1.00**	**1.00**
			Recall	[6,6]	0.89	0.81	0.75	0.68	0.90
				[7,7]	0.92	0.86	0.78	0.70	0.92
		GTDB	F1-score	[6,6]	0.58	0.50	0.50	0.50	0.62
				[7,7]	0.55	0.54	0.50	0.50	0.55
			Precision	[6,6]	0.64	0.64	0.64	0.64	0.66
				[7,7]	0.66	0.64	0.64	0.64	0.66
			Recall	[6,6]	0.53	0.41	0.41	0.41	0.59
				[7,7]	0.48	0.47	0.41	0.41	0.48
		Greengenes	F1-score	[6,6]	0.58	0.57	0.55	0.53	0.64
				[7,7]	0.62	0.55	0.55	0.55	0.67
			Precision	[6,6]	0.66	0.64	0.64	0.63	0.79
				[7,7]	0.78	0.65	0.64	0.64	0.79
			Recall	[6,6]	0.52	0.52	0.49	0.46	0.57
				[7,7]	0.56	0.49	0.49	0.49	0.60
		ITGDB	F1-score	[6,6]	0.92	0.88	0.81	0.71	0.94
				[7,7]	0.94	0.89	0.83	0.73	0.94
			Precision	[6,6]	0.99	0.99	0.98	0.98	**1.00**
				[7,7]	0.99	0.99	0.98	0.98	**1.00**
			Recall	[6,6]	0.87	0.81	0.73	0.65	0.90
				[7,7]	0.89	0.82	0.76	0.67	0.90
		RDP	F1-score	[6,6]	0.63	0.63	0.62	0.58	0.64
				[7,7]	0.64	0.63	0.62	0.59	0.64
			Precision	[6,6]	0.69	0.69	0.68	0.68	0.71
				[7,7]	0.71	0.69	0.68	0.68	0.71
			Recall	[6,6]	0.58	0.58	0.57	0.51	0.59
				[7,7]	0.59	0.58	0.57	0.51	0.59

Continued on next page

Supplementary Table 4: Supplementary table 4. N-gram-range and confidence threshold benchmarking at species level. F1-score, precision and recall are shown for each database, evaluated region and validation datasets. The values are the average of the five metagenomic samples. Bold represents the highest value.

Validation Dataset	Region	Database	Metrics	Conf. thres. / N-gram	**0.5**	**0.7**	**0.9**	**0.98**	**disable**
		SILVA	F1-score	[6,6]	0.85	0.77	0.60	0.47	0.87
				[7,7]	0.87	0.81	0.74	0.60	0.91
			Precision	[6,6]	**1.00**	0.99	0.99	0.94	**1.00**
				[7,7]	**1.00**	**1.00**	0.99	0.99	**1.00**
			Recall	[6,6]	0.74	0.65	0.45	0.33	0.78
				[7,7]	0.78	0.69	0.61	0.48	0.83
	V3-V5	GSR	F1-score	[6,6]	0.97	0.92	0.83	0.76	0.98
				[7,7]	0.98	0.97	0.88	0.79	0.98
			Precision	[6,6]	**1.00**	**1.00**	**1.00**	**1.00**	**1.00**
				[7,7]	**1.00**	**1.00**	**1.00**	**1.00**	**1.00**
			Recall	[6,6]	0.95	0.87	0.77	0.71	0.97
				[7,7]	0.97	0.94	0.82	0.73	0.97
		GTDB	F1-score	[6,6]	0.52	0.51	0.51	0.50	0.62
				[7,7]	0.56	0.51	0.51	0.51	0.56
			Precision	[6,6]	0.67	0.65	0.64	0.64	0.94
				[7,7]	0.67	0.65	0.64	0.64	0.67
			Recall	[6,6]	0.43	0.42	0.42	0.42	0.52
				[7,7]	0.48	0.42	0.42	0.42	0.48
		Greengenes	F1-score	[6,6]	0.57	0.56	0.56	0.54	0.66
				[7,7]	0.61	0.56	0.56	0.56	0.70
			Precision	[6,6]	0.66	0.64	0.64	0.64	0.79
				[7,7]	0.78	0.66	0.64	0.64	0.79
			Recall	[6,6]	0.51	0.50	0.50	0.48	0.58
				[7,7]	0.54	0.51	0.50	0.50	0.64
		ITGDB	F1-score	[6,6]	0.95	0.91	0.79	0.71	0.97
				[7,7]	0.96	0.95	0.84	0.75	0.97
			Precision	[6,6]	0.99	0.99	0.99	0.99	**1.00**
				[7,7]	0.99	0.99	0.99	0.99	**1.00**
			Recall	[6,6]	0.92	0.84	0.71	0.65	0.94
				[7,7]	0.93	0.90	0.76	0.68	0.94
		RDP	F1-score	[6,6]	0.63	0.63	0.60	0.56	0.68
				[7,7]	0.65	0.63	0.63	0.60	0.74
			Precision	[6,6]	0.71	0.71	0.70	0.70	0.73
				[7,7]	0.73	0.71	0.70	0.70	0.97
			Recall	[6,6]	0.58	0.57	0.53	0.48	0.64
				[7,7]	0.59	0.58	0.57	0.53	0.67
		SILVA	F1-score	[6,6]	0.83	0.75	0.58	0.40	0.88
				[7,7]	0.85	0.79	0.66	0.57	0.85
			Precision	[6,6]	**1.00**	**1.00**	0.98	0.92	**1.00**
				[7,7]	**1.00**	**1.00**	**1.00**	0.93	**1.00**
			Recall	[6,6]	0.73	0.60	0.43	0.27	0.80
				[7,7]	0.75	0.66	0.51	0.44	0.75
	V4	GSR	F1-score	[6,6]	0.92	0.89	0.77	0.72	0.94
				[7,7]	0.94	0.90	0.82	0.72	0.95
			Precision	[6,6]	0.98	0.98	0.97	0.95	0.98
				[7,7]	0.98	0.98	0.97	0.95	0.98
			Recall	[6,6]	0.88	0.83	0.71	0.67	0.91
				[7,7]	0.90	0.84	0.76	0.68	0.93
		GTDB	F1-score	[6,6]	0.50	0.50	0.50	0.49	0.57
				[7,7]	0.50	0.50	0.50	0.50	0.56
			Precision	[6,6]	0.63	0.63	0.63	0.62	0.66
				[7,7]	0.63	0.63	0.63	0.63	0.66
			Recall	[6,6]	0.42	0.42	0.42	0.41	0.50
				[7,7]	0.42	0.42	0.42	0.42	0.49
		Greengenes	F1-score	[6,6]	0.55	0.55	0.55	0.54	0.63
				[7,7]	0.57	0.55	0.55	0.55	0.68
			Precision	[6,6]	0.63	0.63	0.63	0.62	0.76
				[7,7]	0.63	0.63	0.63	0.63	0.76
			Recall	[6,6]	0.50	0.50	0.49	0.49	0.57
				[7,7]	0.52	0.50	0.49	0.49	0.62
		ITGDB	F1-score	[6,6]	0.91	0.84	0.72	0.67	0.95

Continued on next page

Supplementary Table 4: Supplementary table 4. N-gram-range and confidence threshold benchmarking at species level. F1-score, precision and recall are shown for each database, evaluated region and validation datasets. The values are the average of the five metagenomic samples. Bold represents the highest value.

Validation Dataset	Region	Database	Metrics	Conf. thres. N-gram	**0.5**	**0.7**	**0.9**	**0.98**	**disable**
				[7,7]	0.91	0.86	0.77	0.68	0.95
			Precision	[6,6]	0.97	0.97	0.95	0.94	0.98
				[7,7]	0.97	0.97	0.95	0.95	0.98
			Recall	[6,6]	0.87	0.77	0.65	0.61	0.92
				[7,7]	0.87	0.79	0.69	0.62	0.92
		RDP	F1-score	[6,6]	0.61	0.61	0.57	0.57	0.67
				[7,7]	0.64	0.61	0.61	0.57	0.67
			Precision	[6,6]	0.70	0.70	0.70	0.69	0.70
				[7,7]	0.70	0.70	0.70	0.69	0.70
			Recall	[6,6]	0.54	0.54	0.49	0.49	0.65
				[7,7]	0.59	0.54	0.54	0.49	0.65
		SILVA	F1-score	[6,6]	0.80	0.66	0.53	0.08	0.82
				[7,7]	0.80	0.70	0.61	0.50	0.86
			Precision	[6,6]	0.97	0.97	0.94	0.33	0.98
				[7,7]	0.97	0.97	0.94	0.94	0.98
			Recall	[6,6]	0.69	0.52	0.39	0.05	0.72
				[7,7]	0.69	0.56	0.46	0.36	0.77
	full-16S	GSR	F1-score	[6,6]	0.98	0.98	0.98	0.93	0.98
				[7,7]	0.98	0.98	0.98	0.97	0.98
			Precision	[6,6]	**1.00**	**1.00**	**1.00**	**1.00**	**1.00**
				[7,7]	**1.00**	**1.00**	**1.00**	**1.00**	**1.00**
			Recall	[6,6]	0.96	0.96	0.95	0.88	0.96
				[7,7]	0.96	0.96	0.96	0.95	0.96
		GTDB	F1-score	[6,6]	0.73	0.65	0.47	0.40	0.78
				[7,7]	0.66	0.62	0.52	0.40	0.67
			Precision	[6,6]	0.88	0.87	0.63	0.62	0.88
				[7,7]	0.88	0.88	0.88	0.62	0.88
			Recall	[6,6]	0.62	0.53	0.37	0.29	0.71
				[7,7]	0.53	0.49	0.38	0.30	0.54
		Greengenes	F1-score	[6,6]	0.72	0.71	0.65	0.59	0.73
				[7,7]	0.71	0.70	0.68	0.66	0.72
			Precision	[6,6]	0.82	0.82	0.78	0.76	0.82
				[7,7]	0.80	0.80	0.81	0.79	0.80
			Recall	[6,6]	0.66	0.64	0.57	0.52	0.68
				[7,7]	0.65	0.64	0.61	0.59	0.66
		ITGDB	F1-score	[6,6]	0.98	0.97	0.96	0.89	0.98
				[7,7]	0.98	0.97	0.96	0.95	0.98
			Precision	[6,6]	0.99	0.99	0.99	0.99	**1.00**
				[7,7]	0.99	0.99	0.99	0.99	**1.00**
			Recall	[6,6]	0.96	0.95	0.92	0.83	0.96
				[7,7]	0.96	0.96	0.93	0.91	0.96
		RDP	F1-score	[6,6]	0.93	0.93	0.88	0.64	0.93
				[7,7]	0.96	0.95	0.94	0.88	0.96
			Precision	[6,6]	0.99	**1.00**	**1.00**	0.96	0.99
				[7,7]	**1.00**	**1.00**	**1.00**	**1.00**	**1.00**
			Recall	[6,6]	0.89	0.88	0.80	0.54	0.89
				[7,7]	0.92	0.91	0.89	0.79	0.92
		SILVA	F1-score	[6,6]	0.89	0.86	0.76	0.58	0.92
				[7,7]	0.96	0.96	0.91	0.87	0.96
			Precision	[6,6]	**1.00**	**1.00**	**1.00**	0.99	**1.00**
				[7,7]	**1.00**	**1.00**	**1.00**	**1.00**	**1.00**
			Recall	[6,6]	0.82	0.78	0.65	0.43	0.85
				[7,7]	0.93	0.92	0.84	0.78	0.93

Supplementary Table 5: Database benchmarking at family levels using validation metrics. F1-score, precision and recall are shown for each database in all the evaluated regions and datasets. Values are the average of the five metagenomic samples. Bold values are the highest and underlined values are the second highest.

Validation Dataset	Region	Metrics	**Greengenes**	**Greengenes2**	**GSR**	**GTDB**	**ITGDB**	**RDP**	**SILVA**
		F1-score	0.35	0.79	0.99	0.80	0.88	0.59	0.74
	V1-V3	Precision	0.36	0.84	0.99	0.85	0.88	0.59	0.83
		Recall	0.35	0.77	0.98	0.78	0.88	0.61	0.71
		F1-score	0.28	0.82	0.98	0.75	0.92	0.82	0.50
	V3-V4	Precision	0.28	0.84	0.99	0.78	0.92	0.87	0.56
		Recall	0.28	0.81	0.98	0.74	0.91	0.81	0.47
		F1-score	0.29	0.82	0.95	0.69	0.87	0.77	0.62
gutmock	V3-V5	Precision	0.30	0.87	0.96	0.72	0.88	0.83	0.74
		Recall	0.30	0.80	0.95	0.68	0.87	0.76	0.59
		F1-score	0.30	0.77	0.93	0.68	0.84	0.87	0.49
	V4	Precision	0.30	0.81	0.94	0.70	0.86	0.88	0.57
		Recall	0.30	0.76	0.92	0.68	0.84	0.87	0.46
		F1-score	0.30	0.83	**1.00**	0.47	0.91	0.88	0.77
	full-16S	Precision	0.30	0.89	**1.00**	0.55	0.91	0.89	0.90
		Recall	0.30	0.80	0.99	0.45	0.91	0.88	0.74
		F1-score	0.27	0.76	0.88	0.81	0.78	0.65	0.85
	V1-V3	Precision	0.30	0.79	0.92	0.87	0.81	0.69	0.88
		Recall	0.26	0.75	0.87	0.79	0.78	0.64	0.84
		F1-score	0.33	0.86	0.90	0.75	0.86	0.51	0.75
	V3-V4	Precision	0.33	0.86	0.90	0.75	0.86	0.51	0.75
		Recall	0.33	0.86	0.90	0.75	0.86	0.51	0.75
		F1-score	0.28	0.67	0.84	0.65	0.79	0.62	0.70
mockrobiota	V3-V5	Precision	0.30	0.70	0.86	0.67	0.83	0.69	0.74
		Recall	0.27	0.67	0.85	0.66	0.80	0.59	0.70
		F1-score	0.21	0.76	0.87	0.54	0.91	0.56	0.79
	V4	Precision	0.21	0.76	0.87	0.54	0.91	0.56	0.79
		Recall	0.21	0.76	0.87	0.54	0.91	0.56	0.79
		F1-score	0.33	0.72	0.77	0.35	0.84	0.76	0.82
	full-16S	Precision	0.36	0.74	0.79	0.37	0.86	0.79	0.84
		Recall	0.32	0.72	0.77	0.34	0.86	0.75	0.82
		F1-score	0.51	0.56	0.87	0.55	0.81	0.30	0.33
	V1-V3	Precision	0.52	0.93	0.96	0.89	0.97	0.27	0.32
		Recall	0.54	0.47	0.83	0.47	0.72	0.35	0.37
		F1-score	0.60	0.74	0.97	0.55	0.94	0.64	0.91
	V3-V4	Precision	0.63	0.96	**1.00**	0.66	**1.00**	0.71	**1.00**
		Recall	0.57	0.62	0.95	0.48	0.90	0.59	0.83
		F1-score	0.61	0.80	0.99	0.56	0.97	0.74	0.85
vagimock	V3-V5	Precision	0.63	0.94	**1.00**	0.67	**1.00**	0.97	**1.00**
		Recall	0.59	0.70	0.99	0.48	0.94	0.67	0.75
		F1-score	0.59	0.77	0.96	0.56	0.95	0.67	0.86
	V4	Precision	0.62	0.89	0.98	0.66	0.98	0.70	0.98
		Recall	0.57	0.68	0.95	0.49	0.92	0.65	0.77
		F1-score	0.61	0.82	0.98	0.67	0.98	0.96	0.96
	full-16S	Precision	0.63	0.98	**1.00**	0.88	**1.00**	**1.00**	**1.00**
		Recall	0.59	0.73	0.96	0.54	0.96	0.92	0.93

Supplementary Table 6: Database benchmarking at family levels using validation metrics. F1-score, precision and recall are shown for each database in all the evaluated regions and datasets. Values are the average of the five metagenomic samples. Bold values are the highest and underlined values are the second highest.

Validation Dataset	Region	Metrics	**Greengenes**	**Greengenes2**	**GSR**	**GTDB**	**ITGDB**	**RDP**	**SILVA**
gutmock	V1-V3	F1-score	0.95	0.97	**1.00**	0.98	0.98	0.97	0.99
		Precision	0.95	0.98	**1.00**	0.98	0.98	0.97	0.99
		Recall	0.95	0.96	**1.00**	0.98	0.98	0.97	0.99
	V3-V4	F1-score	0.92	0.98	**1.00**	0.96	0.98	0.98	0.99
		Precision	0.93	0.99	**1.00**	0.98	0.99	0.99	**1.00**
		Recall	0.92	0.97	0.99	0.96	0.98	0.98	0.98
	V3-V5	F1-score	0.91	0.98	**1.00**	0.96	0.99	0.98	0.98
		Precision	0.92	0.98	**1.00**	0.97	0.99	0.99	0.98
		Recall	0.91	0.98	**1.00**	0.96	0.99	0.98	0.97
	V4	F1-score	0.92	0.98	**1.00**	0.95	0.99	0.98	0.99
		Precision	0.93	0.99	**1.00**	0.97	0.99	0.99	**1.00**
		Recall	0.92	0.97	**1.00**	0.93	0.98	0.98	0.98
	full-16S	F1-score	0.92	0.98	**1.00**	0.93	0.99	0.99	0.99
		Precision	0.94	0.98	**1.00**	0.95	0.99	0.99	**1.00**
		Recall	0.92	0.97	**1.00**	0.92	0.99	0.99	0.98
mockrobiota	V1-V3	F1-score	0.93	0.95	0.96	0.95	0.96	0.84	0.95
		Precision	0.98	0.99	0.99	0.97	0.99	0.87	0.99
		Recall	0.92	0.93	0.95	0.94	0.95	0.84	0.94
	V3-V4	F1-score	0.98	0.99	0.99	0.98	0.99	0.80	0.99
		Precision	0.98	0.99	0.99	0.98	0.99	0.80	0.99
		Recall	0.98	0.99	0.99	0.99	0.99	0.80	0.99
	V3-V5	F1-score	0.95	0.97	0.98	0.96	0.99	0.88	0.98
		Precision	0.97	0.99	0.99	0.97	0.99	0.95	0.99
		Recall	0.94	0.96	0.98	0.95	0.99	0.87	0.97
	V4	F1-score	0.98	0.99	0.99	0.90	0.99	0.84	0.99
		Precision	0.98	0.99	0.99	0.92	0.99	0.84	0.99
		Recall	0.98	0.99	0.99	0.90	0.99	0.84	0.99
	full-16S	F1-score	0.95	0.96	0.98	0.96	**1.00**	0.86	0.97
		Precision	0.98	0.99	0.99	0.96	**1.00**	0.87	0.99
		Recall	0.94	0.95	0.98	0.97	0.99	0.86	0.96
vagimock	V1-V3	F1-score	**1.00**	**1.00**	**1.00**	**1.00**	**1.00**	0.99	**1.00**
		Precision	**1.00**	**1.00**	**1.00**	**1.00**	**1.00**	0.99	**1.00**
		Recall	**1.00**	**1.00**	**1.00**	**1.00**	**1.00**	0.99	**1.00**
	V3-V4	F1-score	**1.00**	**1.00**	**1.00**	**1.00**	**1.00**	**1.00**	**1.00**
		Precision	**1.00**	**1.00**	**1.00**	**1.00**	**1.00**	**1.00**	**1.00**
		Recall	**1.00**	**1.00**	**1.00**	**1.00**	**1.00**	**1.00**	**1.00**
	V3-V5	F1-score	**1.00**	**1.00**	**1.00**	**1.00**	**1.00**	**1.00**	**1.00**
		Precision	**1.00**	**1.00**	**1.00**	**1.00**	**1.00**	**1.00**	**1.00**
		Recall	**1.00**	**1.00**	**1.00**	**1.00**	**1.00**	**1.00**	**1.00**
	V4	F1-score	**1.00**	**1.00**	**1.00**	**1.00**	**1.00**	**1.00**	**1.00**
		Precision	**1.00**	**1.00**	**1.00**	**1.00**	**1.00**	**1.00**	**1.00**
		Recall	**1.00**	**1.00**	**1.00**	**1.00**	**1.00**	**1.00**	**1.00**
	full-16S	F1-score	**1.00**	**1.00**	**1.00**	0.99	**1.00**	**1.00**	**1.00**
		Precision	**1.00**	**1.00**	**1.00**	0.99	**1.00**	**1.00**	**1.00**
		Recall	**1.00**	**1.00**	**1.00**	0.99	**1.00**	**1.00**	**1.00**

Supplementary Table 7: Database benchmarking at genus level using validation metrics. F1-score, precision and recall are shown for each database in all the evaluated regions and datasets. Values are the average of the five metagenomic samples. Bold values are the highest and underlined values are the second highest.

Validation Dataset	Region	Metrics	**Greengenes**	**Greengenes2**	**GSR**	**GTDB**	**ITGDB**	**RDP**	**SILVA**
gutmock	V1-V3	F1-score	0.49	0.85	0.98	0.84	0.89	0.71	0.93
		Precision	0.66	0.87	0.99	0.87	0.90	0.75	0.94
		Recall	0.53	0.85	0.98	0.83	0.90	0.71	0.92
	V3-V4	F1-score	0.51	0.89	0.99	0.86	0.92	0.91	0.89
		Precision	0.62	0.91	**1.00**	0.87	0.93	0.92	0.92
		Recall	0.54	0.89	0.99	0.86	0.92	0.90	0.88
	V3-V5	F1-score	0.52	0.88	0.98	0.85	0.91	0.90	0.91
		Precision	0.65	0.90	0.99	0.87	0.92	0.92	0.93
		Recall	0.56	0.88	0.98	0.85	0.91	0.90	0.91
	V4	F1-score	0.55	0.89	0.96	0.82	0.89	0.91	0.88
		Precision	0.70	0.90	0.96	0.84	0.91	0.92	0.91
		Recall	0.58	0.88	0.97	0.81	0.90	0.90	0.86
	full-16S	F1-score	0.56	0.87	**1.00**	0.75	0.93	0.91	0.94
		Precision	0.70	0.89	**1.00**	0.79	0.94	0.92	0.95
		Recall	0.60	0.87	**1.00**	0.75	0.93	0.91	0.94
mockrobiota	V1-V3	F1-score	0.79	0.86	0.90	0.85	0.83	0.80	0.92
		Precision	0.81	0.90	0.93	0.89	0.86	0.83	0.96
		Recall	0.80	0.85	0.90	0.84	0.83	0.79	0.92
	V3-V4	F1-score	0.86	0.98	0.99	0.98	0.99	0.80	0.99
		Precision	0.85	0.98	0.99	0.98	0.99	0.80	0.99
		Recall	0.89	0.98	0.99	0.98	0.99	0.80	0.99
	V3-V5	F1-score	0.71	0.83	0.87	0.83	0.89	0.83	0.86
		Precision	0.72	0.85	0.88	0.85	0.90	0.85	0.92
		Recall	0.75	0.85	0.88	0.84	0.90	0.82	0.87
	V4	F1-score	0.82	0.95	0.96	0.88	0.99	0.79	0.98
		Precision	0.81	0.95	0.96	0.89	0.99	0.79	0.98
		Recall	0.85	0.95	0.96	0.88	0.99	0.79	0.98
	full-16S	F1-score	0.75	0.83	0.86	0.79	0.89	0.83	0.87
		Precision	0.75	0.84	0.87	0.82	0.90	0.85	0.92
		Recall	0.78	0.84	0.87	0.80	0.90	0.83	0.88
vagimock	V1-V3	F1-score	**1.00**	0.91	**1.00**	0.89	0.95	0.94	0.95
		Precision	**1.00**	0.99	**1.00**	0.93	**1.00**	0.99	**1.00**
		Recall	**1.00**	0.86	**1.00**	0.85	0.91	0.90	0.90
	V3-V4	F1-score	**1.00**	0.92	**1.00**	0.92	0.97	0.97	0.97
		Precision	0.99	0.95	**1.00**	0.95	**1.00**	**1.00**	**1.00**
		Recall	**1.00**	0.90	**1.00**	0.90	0.94	0.94	0.94
	V3-V5	F1-score	**1.00**	0.91	**1.00**	0.91	0.98	0.97	0.98
		Precision	**1.00**	0.94	**1.00**	0.93	**1.00**	**1.00**	**1.00**
		Recall	**1.00**	0.89	**1.00**	0.88	0.96	0.94	0.95
	V4	F1-score	**1.00**	0.90	0.99	0.90	0.96	0.96	0.96
		Precision	**1.00**	0.94	**1.00**	0.93	**1.00**	**1.00**	**1.00**
		Recall	**1.00**	0.87	0.98	0.86	0.93	0.93	0.92
	full-16S	F1-score	**1.00**	0.93	0.98	0.91	0.98	0.98	0.98
		Precision	**1.00**	0.99	**1.00**	0.92	**1.00**	**1.00**	**1.00**
		Recall	**1.00**	0.91	0.96	0.89	0.96	0.96	0.96

Supplementary Table 8: Database benchmarking using Bray-Curtis distances between the expected and observed composition at family, genus and species level. Values are the average of the five metagenomic samples. Bold values are the highest

Validation Dataset	Region	Level	**Greengenes**	**Greengenes2**	**GSR**	**GTDB**	**ITGDB**	**RDP**	**SILVA**
gutmock	V1-V3	Family	0.03	0.04	**0.00**	0.02	0.01	0.03	0.01
		Genus	0.43	0.14	0.02	0.15	0.09	0.27	0.08
		Species	0.65	0.23	0.02	0.22	0.11	0.39	0.29
	V3-V4	Family	0.06	0.02	0.01	0.04	0.01	0.02	0.02
		Genus	0.44	0.11	0.01	0.14	0.08	0.10	0.12
		Species	0.72	0.19	0.02	0.26	0.09	0.19	0.53
	V3-V5	Family	0.06	0.02	**0.00**	0.03	0.01	0.01	0.02
		Genus	0.41	0.11	0.02	0.12	0.08	0.10	0.09
		Species	0.70	0.20	0.05	0.32	0.13	0.24	0.41
	V4	Family	0.07	0.03	**0.00**	0.06	0.01	0.02	0.02
		Genus	0.41	0.11	0.03	0.18	0.10	0.10	0.13
		Species	0.70	0.24	0.08	0.32	0.16	0.13	0.54
	full-16S	Family	0.06	0.03	**0.00**	0.06	0.01	0.01	0.02
		Genus	0.38	0.12	**0.00**	0.22	0.06	0.09	0.06
		Species	0.70	0.20	0.01	0.54	0.09	0.12	0.26
mockrobiota	V1-V3	Family	0.08	0.07	0.05	0.04	0.05	0.14	0.06
		Genus	0.20	0.15	0.10	0.16	0.16	0.21	0.08
		Species	0.74	0.25	0.13	0.21	0.22	0.36	0.16
	V3-V4	Family	0.02	0.01	0.01	0.01	0.01	0.20	0.01
		Genus	0.11	0.02	0.01	0.02	0.01	0.20	0.01
		Species	0.67	0.14	0.10	0.25	0.14	0.49	0.25
	V3-V5	Family	0.06	0.04	0.02	0.05	0.01	0.13	0.03
		Genus	0.25	0.15	0.12	0.16	0.08	0.18	0.13
		Species	0.73	0.33	0.15	0.34	0.20	0.41	0.30
	V4	Family	0.02	0.01	0.01	0.09	0.01	0.16	0.01
		Genus	0.15	0.05	0.04	0.12	0.01	0.21	0.02
		Species	0.79	0.24	0.13	0.46	0.09	0.44	0.21
	full-16S	Family	0.06	0.05	0.02	0.03	0.01	0.14	0.04
		Genus	0.22	0.16	0.13	0.20	0.09	0.17	0.12
		Species	0.68	0.28	0.23	0.66	0.15	0.25	0.18
vagimock	V1-V3	Family	**0.00**	**0.00**	**0.00**	**0.00**	**0.00**	0.01	**0.00**
		Genus	**0.00**	0.14	**0.00**	0.15	0.09	0.10	0.10
		Species	0.41	0.50	0.17	0.53	0.28	0.65	0.63
	V3-V4	Family	**0.00**	**0.00**	**0.00**	**0.00**	**0.00**	**0.00**	**0.00**
		Genus	**0.00**	0.10	**0.00**	0.10	0.06	0.06	0.06
		Species	0.43	0.38	0.05	0.52	0.10	0.41	0.17
	V3-V5	Family	**0.00**	**0.00**	**0.00**	**0.00**	**0.00**	**0.00**	**0.00**
		Genus	**0.00**	0.11	**0.00**	0.12	0.04	0.06	0.05
		Species	0.41	0.30	0.01	0.52	0.06	0.33	0.25
	V4	Family	**0.00**	**0.00**	**0.00**	**0.00**	**0.00**	**0.00**	**0.00**
		Genus	**0.00**	0.13	0.02	0.14	0.07	0.07	0.08
		Species	0.43	0.32	0.05	0.51	0.08	0.35	0.23
	full-16S	Family	**0.00**	**0.00**	**0.00**	0.01	**0.00**	**0.00**	**0.00**
		Genus	**0.00**	0.09	0.04	0.11	0.04	0.04	0.04
		Species	0.41	0.25	0.04	0.46	0.04	0.08	0.07
